# Intrinsic Force–Temperature Self-Decoupling Enables Human-like Tactile Sensing in a Soft Ionic Skin

**DOI:** 10.1021/acsnano.6c09063

**Published:** 2026-07-25

**Authors:** Yu Feng, Jiankun Li, Cong Wu, Senlin Hou, Yehui Liu, Hui Sun, Meng Chen, Ziyi Li, Zhi Li, Guanglie Zhang, Steven Wang, Vellaisamy A. L. Roy, Xinge Yu, Wen Jung Li

**Affiliations:** † Department of Mechanical Engineering, 53025 City University of Hong Kong, Kowloon, Hong Kong SAR 999077, China; ‡ CAS-CityU Joint Laboratory for Robotic Research, City University of Hong Kong, Kowloon, Hong Kong SAR 999077, China; § College of Computer Science and Software Engineering, 47890 Shenzhen University, Shenzhen 518000, China; ∥ School of Science and Technology, 66386 Hong Kong Metropolitan University, Kowloon, Hong Kong SAR 999077, China; ⊥ Department of Biomedical Engineering, 53025 City University of Hong Kong, Kowloon, Hong Kong SAR 999077, China; # School of Engineering and Technology, China University of Geosciences (Beijing), Beijing 100083, China

**Keywords:** flexible sensor, bioinspired sensor, multimodal tactile sensor, tactile sensing, robotic sensing, robotic manipulation, ionic gel

## Abstract

Soft dual-modal tactile sensors capable of simultaneously sensing force and temperature are essential for enhancing human-like perception and interaction in robots, particularly in the functional sense of concurrent mechanical and thermal perception. However, achieving self-decoupled and high-fidelity dual-modal sensing remains a significant challenge due to intrinsic signal crosstalk, structural complexity, and limited flexibility in existing designs. Here, we present a soft robotic tactile (RoboTac) skin that intrinsically decouples force and temperature using an ionic conductive film within a minimalist architecture, featuring an ultralight weight and an ultralow cost. Ionic conductivity enables independent readouts without algorithmic compensation by allowing thickness compression to modulate capacitance (force) and lateral ionic transport under thermal stimuli to modulate resistance (temperature). Moreover, the RoboTac skin demonstrates its practical utility for robots in object perception, specialized tasks, and human–robot interaction. This work establishes a general principle for intrinsically self-decoupling modalities in tactile sensors, advancing multimodal sensing, intelligent perception, and embodied robotics.

## Graphical abstract

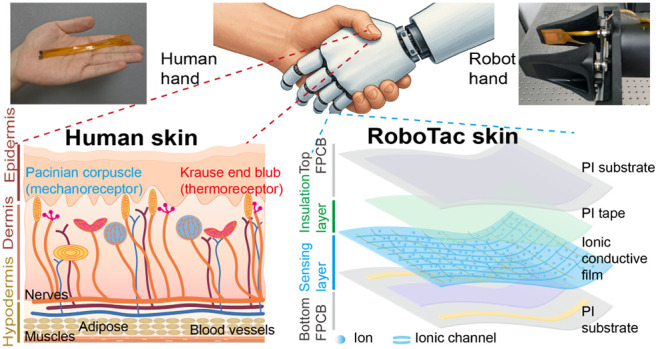

## Introduction

With the rise of embodied intelligence, empowering robots with cognitive capabilities through algorithmic frameworks such as large language models has become increasingly feasible.^1–4^ However, achieving natural and flexible interaction between robots and the physical world requires not only computational intelligence but also the support of sensing functions, such as visual, auditory, olfactory, and tactile perception. Among these, tactile sensing plays a critical role in enabling robots to perceive and interact with their surroundings, serving as a foundation for high-quality robot–world interaction. In human perception, tactile feedback is indispensable, accounting for approximately 20% of the interactive information exchanged between humans and the environment.^5–7^ It provides rich physical insights such as the shape, texture, stiffness, material type, and temperature of contacted objects, primarily through mechanoreceptors and thermoreceptors embedded in the skin.^8–11^ From a physical quantity perspective, tactile perception fundamentally involves sensing force and temperature. Therefore, the development of tactile sensors capable of simultaneously detecting force and temperature is essential for robots, according to the human somatosensory system.

Meanwhile, to enable conformal integration on robot surfaces, these sensors need to be mechanically soft. Advances in materials science and nanotechnology have spurred the emergence of numerous soft tactile sensors that utilize functional materials such as conductive polymers,^12–14^ liquid metals,^15–17^ and two-dimensional materials,^18–20^ along with sophisticated structural designs including microstructured arrays,^21–23^ multilayered architectures,^24–26^ and bioinspired geometries.^27,28^ Among them, soft force sensors based on piezoresistive,^29,30^ piezoelectric,^31,32^ optical,^33–35^ and Hall effect^36–38^ principles have demonstrated strong capabilities in force sensing. However, these sensors typically lack a temperature-sensing function and are likely prone to signal drift under ambient temperature fluctuations. Conversely, soft temperature sensors based on thermoelectric^39,40^ or thermosensitive^41^ mechanisms can accurately capture temperature changes but often fail to detect mechanical stimuli or exhibit measurement errors under mechanical perturbation.

To bridge this gap, dual-modal tactile sensors that integrate both force and temperature sensing have been proposed (Table S1). Some dual-modal tactile sensors achieve high-sensitivity detection of both force and temperature by calibrating the responses of a single sensing layer.^42,43^ However, due to the intrinsic coupling between mechanical and thermal stimuli in such materials, these devices are generally unable to provide simultaneous and decoupled measurements of force and temperature. To address this limitation, specific sensing integration, which involves isolating and protecting the sensing layers to reduce signal crosstalk, offers a way to simultaneously detect force and temperature signals. For instance, some designs incorporate thermoelectric-based temperature sensing units along with buffer layers to suppress mechanical interference.^44–46^ Nevertheless, these configurations often exhibit limited flexibility and low integration density, which hinders their application in compact systems. Although software decoupling calibrates actual force responses based on precharacterized force responses at different temperatures with easy implementation,^47–49^ its decoupling efficiency still requires improvement. Alternatively, multilayer structures or array-based architectures, as hardware decoupling, have been developed to physically separate the force-sensing and temperature-sensing elements within a single device, allowing for independent and decoupled signal readout.^50–53^ Despite their effectiveness, such designs usually involve complex structures and increased fabrication challenges. Aforementioned solutions face these existing limitations, such as complex architectures and costly fabrication, poor flexibility and integration compatibility, and non-negligible signal crosstalk. These issues not only impair overall sensing performance but also hinder their practical deployment in robotic end-effectors, especially in scenarios requiring high flexibility and signal quality.

Here, to tackle this dilemma (Figure 1I), we present a soft robotic tactile skin (RoboTac skin) with intrinsically self-decoupled dual-modal sensing (Figure 1A). Based on a standard and concise four-layer sandwich structure and an easily processable ionic conductive film (Figure 1B), RoboTac skin enables intrinsically self-decoupled force and temperature sensing with an ultralight weight (0.15 g) and ultralow cost (0.45 USD). This specific sandwich structure generates two electrical signals (Figure 1E), i.e., a capacitance formed by the top and bottom flexible printed circuit boards (FPCBs), and a resistance at the bottom FPCB. Due to the ionic conductivity mechanism of the sensing film, thermal stimuli predominantly modulate lateral ionic transport, whereas mechanical compression primarily changes the film thickness. As a result, the capacitance channel responds predominantly to mechanical stimuli, with only a small temperature-induced variation, whereas the resistance channel responds predominantly to thermal stimuli and exhibits limited interference from mechanical loading. We further confirmed the intrinsically self-decoupled dual-modal sensing ability through electrochemical analysis and conducted sensing performance tests using a dual-modal experimental platform. Combining simple structure, ultralight weight, ultralow cost, and easy fabrication, RoboTac skin enables robots to acquire stable and high-quality force and temperature signals via a simple approach (sensing performance comparison in Table S2). Finally, as illustrated in Figure 1J, via easy deployment of RoboTac skin, we demonstrated its efficacy in enhancing robots with object perception (object classification and materials identification), special tasks (object transfer and laboratory automation), and human-robot interaction (assistive service and medical healthcare). This study is expected to greatly enhance robotic tactile perception, improve the generalizability of robot-world interaction, and further inspire research in multimodal sensors, intelligent perception, and embodied robotics.

**Figure 1:**
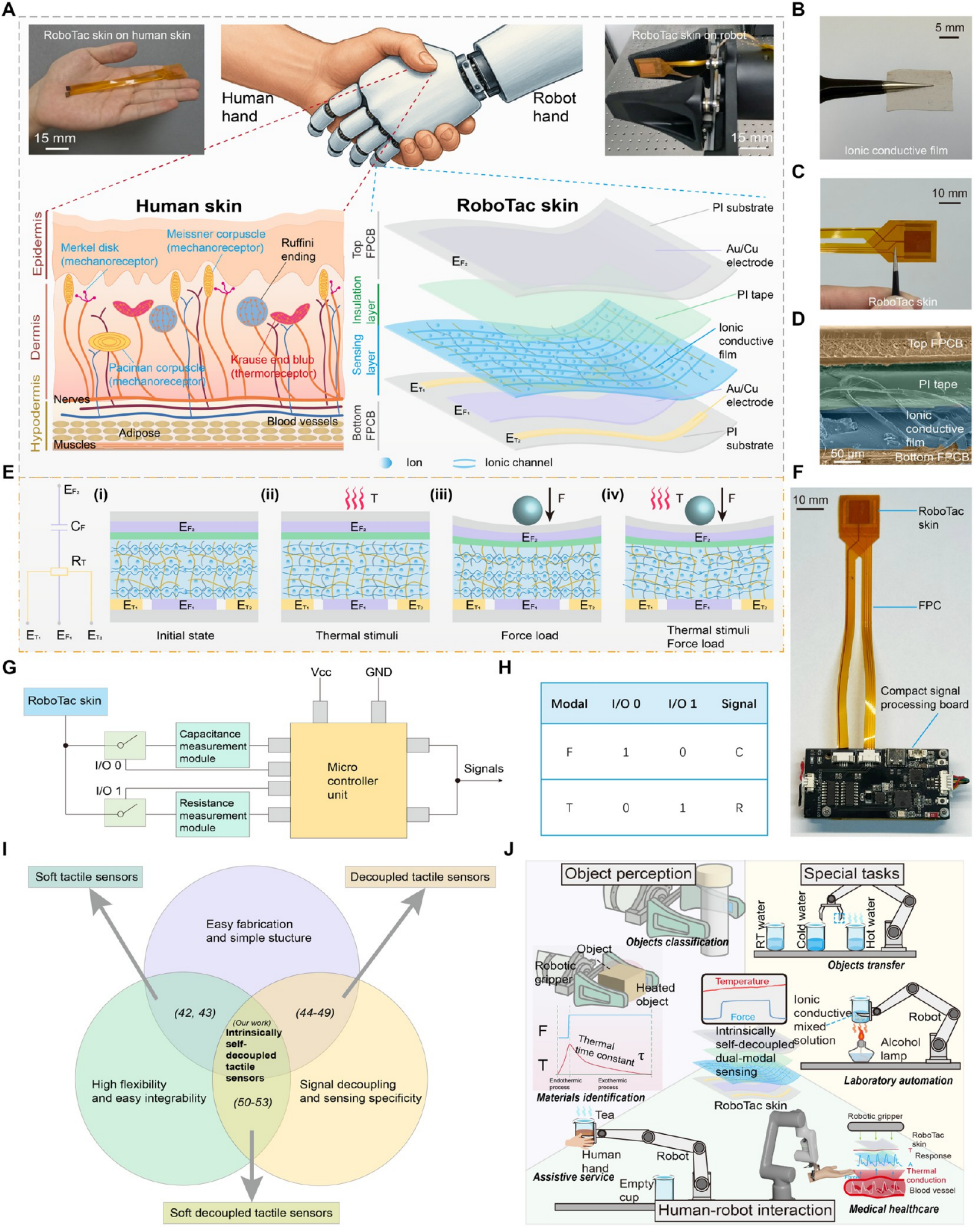
Overview of RoboTac skin with intrinsically self-decoupled dual-modal sensing. (A) Illustration of human skin structure and RoboTac skin structure, and optical photos of RoboTac skin on a human hand and a robotic gripper. The mechanoreceptors and thermoreceptors are shown as functional biological inspiration for modality-level force and temperature perception, rather than as direct morphological or neural analogues. (B) Optical photo of the ionic conductive film. (C) Optical photo of RoboTac skin. (D) SEM photo of the cross-section of RoboTac skin. (E) Illustration of the intrinsically self-decoupled dual modal sensing mechanism of RoboTac skin. (F) Optical photo of RoboTac skin and the compact signal processing board. (G) Schematic diagram of the compact signal processing system. (H) Working principle of the dual-modal signal measurement of the compact signal processing system. (I) Comparison of various tactile sensors, highlighting the RoboTac skin’s unique ability of intrinsically self-decoupled dual-modal sensing. (J) RoboTac skin enables robots with intrinsically self-decoupled dual-modal sensing, empowering their better performance for object perception, special tasks, and human-robot interaction.

## Results and Discussion

### Design and Principle of RoboTac Skin

As shown in Figure 1A, inspired by the multilayered structure of human skin (epidermis, dermis, hypodermis), we designed RoboTac skin with a four-layer sandwich configuration consisting of a top FPCB, insulation layer, sensing layer, and bottom FPCB. Drawing further inspiration from the dual-modal perception function of mechanoreceptors (Meissner corpuscle, Pacinian corpuscle, Merkel disk) and thermoreceptors (Krause end bulb) in human skin, the device achieves intrinsically self-decoupled dual-modal sensing of force and temperature via the ionic conduction mechanism of its sensing layer (an ionic conductive film). As shown in Figure 1C, the four layers (from top to bottom) are a polyimide (PI) substrate, PI tape, ionic conductive film, and PI substrate (Au/Cu electrodes), with a cross-sectional SEM image provided in Figure 1D.

The core of the intrinsically self-decoupled dual-modal sensing capability of RoboTac skin lies in the ionic conductivity mechanism of the ionic conductive film,^54,55^ as illustrated in Figure 1E. Along the *z*-axis, RoboTac skin forms a capacitance (C_F_) between electrodes E_F1_ and E_F2_, while in the xy-plane, it forms a resistance (R_T_) between electrodes E_T1_ and E_T2_. The C_F_ and R_T_ are constructed as two independent channels and do not share the same signal circuit. As shown in Figure 1E (i), when there is no external force (F) or thermal (T) stimulus, both C_F_ and R_T_ remain at their initial values and do not change. When only a thermal stimulus is applied, as shown in Figure 1E (ii), the ionic flow rate in the xy-plane of the ionic conductive film increases, thereby decreasing R_T_ and enabling a negative temperature coefficient (NTC) sensing mechanism. At the same time, due to ionic confinement in the *z*-direction, C_F_ remains nearly unchanged, indicating effective suppression of thermal interference in the capacitance channel. When only a mechanical stimulus is applied, as shown in Figure 1E (iii), the ionic flow rate in the xy-plane remains unchanged, while compression in the *z*-direction leads to a change in thickness and an increase in C_F_, resulting in a force-sensing mechanism based on capacitance. In this case, R_T_ remains nearly unchanged under mechanical input, indicating effective suppression of force-induced interference in the resistance channel. Finally, as shown in Figure 1E (iv), when both force and thermal stimuli are present, the ionic flow rate in the xy-plane increases, decreasing R_T_, while the thickness in the *z*-direction decreases, increasing C_F_. Due to ionic confinement, *z*-direction ion movement is strongly restricted, and the variations in C_F_ and R_T_ are dominated by mechanical and thermal stimuli, respectively, with only limited cross-channel interference. Therefore, through the ionic conductivity mechanism of the ionic conductive film and the designed electrode structure, RoboTac skin achieves intrinsically self-decoupled force and temperature sensing with low cross-channel interference.

Figure 1F shows the dual-modal measurement system of RoboTac skin, in which the sensor is linked to a compact signal processing board via FPC. As illustrated in Figure 1G, the RoboTac skin interfaces with a microcontroller through two independent channels, namely a capacitance measurement module for C_F_ and a resistance measurement module for R_T_, and the microcontroller is subsequently connected to a PC. In operation (Figure 1H), enabling I/O 0 while disabling I/O 1 allows C_F_ variations to be recorded for force measurement, whereas enabling I/O 1 while disabling I/O 0 records R_T_ variations for temperature measurement. Through signal acquisition and processing by the compact board, RoboTac skin provides independently acquired force and temperature signals with low cross-channel interference. By integrating a carefully designed structure with a uniquely functional sensing material, we have developed a device with intrinsically self-decoupled dual-modal sensing capability, laying the foundation for endowing robots with embodied tactile perception.

### Fabrication and Characterization of RoboTac Skin

The sensing layer, a key component of RoboTac skin, was prepared using a simple one-pot, three-step process (Figure 2A). Pectin (PEC), chitosan (CHI), glycerol (GLY), PEG, CaCl_2_, and citric acid were dissolved in deionized water by stirring in a water bath at 80 °C for 2 h, followed by 1 h of ultrasonication to remove air bubbles. The resulting mixture was dried at 80 °C for 2 h and cut into the desired shape to obtain the ionic conductive film. The sensing layer is an ionic conductive film that is soft and flexible, providing a mechanical foundation for a soft tactile sensor. As shown in Figure 2B and Movie S3, the sensing layer is easy to stretch, fold, twist, and squeeze, laying the foundation for constructing a soft tactile sensor. After the ionic conductive film was prepared, it was encapsulated into the RoboTac skin (total mass 0.15 g) using a simple method involving common PI tape and an FPCB within 150 s, as shown in Movie S2. Notably, the total cost of a single RoboTac skin is only 0.45 USD (Table S4), while the cost of a single ionic conductive film is merely 0.005 USD (Table S5). The combination of a straightforward fabrication process and low material cost will make the large-scale production of RoboTac skin feasible in the future.

**Figure 2:**
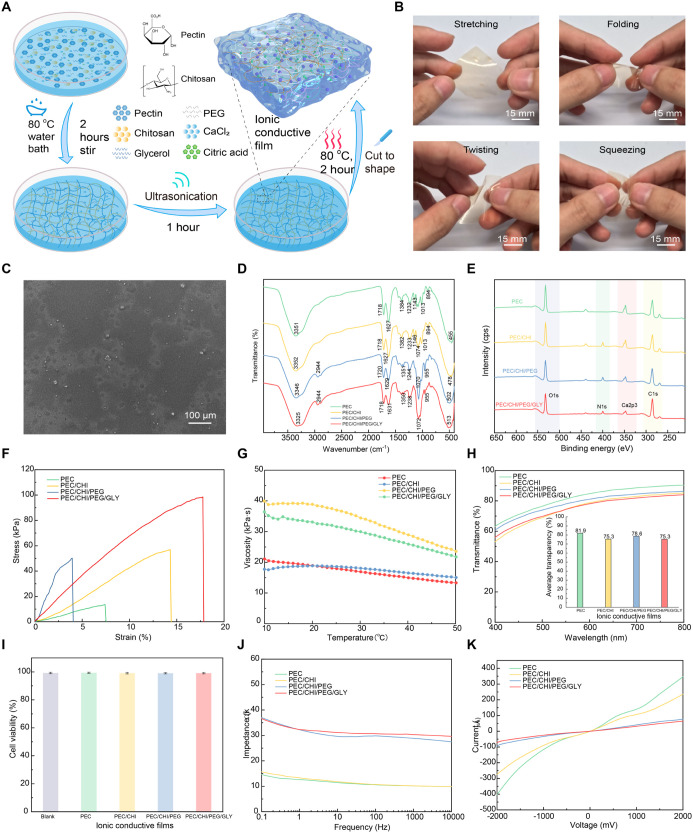
Fabrication and characterization of RoboTac skin. (A) Schematic illustration of the fabrication of the ionic conductive film. (B) Optical images of the deformation of the ionic conductive film. (C) SEM images of the surface of the ionic conductive film. (D) FTIR spectra of the ionic conductive films. (E) XPS spectra of the ionic conductive films. (F) Tensile stress–strain curves of the ionic conductive films. (G) Viscosity of the ionic conductive films under various temperature conditions. (H) UV–vis spectra of the ionic conductive films. (I) Cell viability of the ionic conductive films. (J) EIS characteristics of the ionic conductive films. (K) I–V characteristics of the ionic conductive films.

SEM images (Figure 2C and Figure S1) reveal smooth and uniform surfaces for all films, with PEC/CHI/PEG/GLY showing the highest degree of uniformity. In addition, Fourier transform infrared spectroscopy (FTIR) was used to further characterize the four samples. As illustrated in Figure 2D, the FTIR spectra confirm the synergistic interactions in the PEC/CHI/PEG/GLY film. A broadened and red-shifted O–H/N–H stretching band (∼3325 cm^–1^) indicates enhanced hydrogen bonding among pectin, chitosan, PEG, and glycerol. Increased intensity of C–H (2944 cm^–1^) and C–O–C (1072–1013 cm^–1^) bands reflects the incorporation of flexible ether linkages, facilitating ionic transport and deformability. Meanwhile, characteristic peaks of carboxyl (1720 cm^–1^) and Ca–O (500 cm^–1^) confirm the retention of the Ca^2+^-cross-linked network, ensuring mechanical stability. These features underpin a material that is both flexible and robust, suitable for tactile sensors. Also, these films were investigated through X-ray photoelectron spectroscopy (XPS) in Figure 2E and Figure S2. The XPS spectra of the four films reveal distinct elemental signals corresponding to O1s, N1s, Ca2p3, and C1s, indicating the presence of oxygen, nitrogen, calcium, and carbon in the samples. The XPS spectra confirm that the PEC/CHI/PEG/GLY film possesses abundant oxygen-containing groups (O1s), preserved nitrogen functionalities from chitosan (N1s), and a stable Ca^2+^-cross-linked network (Ca2p3). The broadened C 1s peak indicates increased C–O/C–O–C content from PEG and glycerol, consistent with enhanced flexibility and ionic transport capacity.

Moreover, the mechanical and rheological properties of the films were systematically evaluated. The tensile stress–strain curves of the four hydrogel formulations reveal distinct mechanical behaviors (Figure 2F). The PEC film showed the lowest strength (∼15 kPa) and limited extensibility (∼6%), indicating poor mechanical integrity. Adding chitosan (PEC/CHI) improved both strength (∼55 kPa) and elongation (∼15%). The PEG-containing sample (PEC/CHI/PEG) maintained relatively high strength (∼46 kPa) but failed at a lower strain (∼4.5%), suggesting increased brittleness. In contrast, PEC/CHI/PEG/GLY exhibited the highest strength and best flexibility, with the highest tensile strength (over 100 kPa) and elongation (∼18%), attributed to the plasticizing effect of glycerol. The viscosity-temperature profiles of the four ionic conductive films (Figure 2G) exhibit typical thermoresponsive behavior, with viscosity decreasing as temperature increases. The PEC/CHI/PEG/GLY film exhibits an intermediate-high viscosity with a smooth decline from 10 to 50 °C, indicating a balanced network that is both stable and compliant. Compared to the high-viscosity but temperature-sensitive PEC/CHI/PEG, the addition of glycerol enhances thermal stability, ensuring consistent processability and mechanical performance over a broad temperature range. As shown in Figure S3A and B, the PEC/CHI/PEG/GLY film maintains solid-like behavior (G’>G″) across a broad deformation range with lower stiffness than stiffer counterparts, ensuring conformal contact. Also, it shows smooth and predictable softening from 10 to 50 °C with stable viscoelastic balance (Figure S3C), combining CHI-reinforced network integrity with PEG/GLY-induced flexibility for skin-interfacing applications. Additionally, the ultraviolet–visible spectroscopy (UV–vis) tests were carried out to study the transparency of the ionic conductive films. Figure 2H presents the UV–vis transmittance spectra of the ionic conductive hydrogel films in the wavelength range of 400–800 nm. All films exhibited increasing transmittance with wavelength, with average values above 75% across the visible range (PEC/CHI/PEG/GLY 75.3%). Cell viability experiments were also conducted to investigate the biocompatibility of the ionic conductive films (Figure S4). As shown in Figure 2I, the cell viability of these films is all above 95%, confirming their biocompatibility.

Furthermore, the electrochemical properties of ionic conductive films were also studied by using the electrochemical analyzer. Figure 2J shows the electrochemical impedance spectroscopy (EIS) characteristics of the ionic conductive films in the range of 0.1–10 kHz. The impedance of all films generally decreases with increasing frequency, exhibiting a stable plateau at high frequencies (above 1000 Hz) and more pronounced variation at low frequencies (below 10 Hz). This behavior suggests that ion transport, rather than electronic conduction, dominates the interfacial polarization effects in these films. Additionally, the incorporation of PEG results in an increase in overall impedance, indicating that PEG may hinder ion mobility within the hydrogel matrix, possibly due to increased viscosity and enhanced hydrogen bonding networks. Figure 2K presents the current–voltage (I–V) characteristics of the films, all of which exhibit good linearity. As CHI, PEG, and GLY are sequentially introduced, the slope of the I–V curves gradually decreases, indicating a reduction in ionic conductivity. This trend is consistent with the frequency impedance results. In the cyclic voltammetry (CV) comparison of different formulations (Figure S5A), PEC exhibits the highest current response but suffers from pronounced polarization and curve distortion. Incorporation of CHI markedly decreases the current due to the denser network limiting ion transport, while the addition of PEG restores part of the current and improves the rectangularity of the CV profile. The PEC/CHI/PEG/GLY film achieved a symmetric CV curve with minimal polarization, maintaining its shape over 50 cycles (Figure S5B), which indicates a well-balanced combination of ionic conductivity, electrochemical stability, and cycling durability. EIS analysis shows a small interception in the Nyquist plot with no visible high-frequency semicircle and a low-frequency inclined line, suggesting low bulk resistance and ion-diffusion-dominated transport (Figure S5C). Additionally, the phase angle starts at approximately −50° at low frequency and increases smoothly with frequency without abrupt changes, indicating a predominantly capacitive response that is stable across a broad frequency range (Figure S5D). Collectively, the PEC/CHI/PEG/GLY formulation was selected as the sensing layer due to its optimal balance of properties, combining high mechanical strength and flexibility, stable viscoelasticity over a broad temperature range, sufficient optical transparency, low interfacial resistance with fast ion transport, and excellent electrochemical stability, while maintaining outstanding biocompatibility, which makes it ideally suited for sensing layer in RoboTac skin.

### Principle of Intrinsically Self-Decoupled Sensing of RoboTac Skin

RoboTac skin’s intrinsically self-decoupled dual-modal sensing arises from the deliberate design of its multilayer structure. As shown in Figure 3A, the ionic conductive film is the key component of the sensing layer. The sensing layer is positioned on the bottom FPC (electrodes E_T1_, E_T2_, and E_F1_), while the top FPC (electrode E_F2_) is placed above it with an insulation layer in between. Figure 3B illustrates the self-decoupling mechanism, which is governed by ion conduction within the film (Movie S1). At the initial state without external force or temperature change (F = 0, T = RT; Figure 3B (i)), most Ca^2+^ ions are bound within the cross-linking sites of the pectin network, while only a small fraction remains loosely associated or free to migrate. The film exhibits an initial capacitance C_F0_ and resistance R_T0_. Under mechanical loading without temperature change (F > 0, T = RT; Figure 3B (ii)), the ionic cross-linking network remains unchanged, restricting directional ion migration along the xy-plane (ionic confinement). Consequently, the electrical resistance remains approximately constant (R_T_ = R_T0_). Meanwhile, compression along the *z*-axis reduces the film thickness and increases local ionic accumulation, resulting in higher capacitance (C_F_ > C_F0_). Under heating without mechanical load (F = 0, *T* > RT; Figure 3B (iii)), the ionic cross-linking network is thermally weakened, releasing additional mobile Ca^2+^ ions. These ions migrate along the xy-plane with an increased flow rate, thereby reducing the electrical resistance (R_T_ < R_T0_). Meanwhile, because the film thickness changes only slightly under thermal stimulation, the capacitance remains nearly unchanged (C_F_ = C_F0_). When both force and heating are applied (F > 0, T > RT; Figure 3B (iv)), mobile ions migrate along the xy-plane while ionic confinement along the *z*-axis persists. Consequently, the electrical resistance decreases (R_T_ < R_T0_) and the capacitance increases (C_F_ > C_F0_) owing to the combined effects of enhanced ionic accumulation and film thickness reduction. Through the interplay of *z*-axis ionic confinement and xy-plane ionic flow, the film produces predominantly selective capacitance and resistance responses with low cross-channel interference, thereby enabling intrinsically self-decoupled sensing of force and temperature. The theoretical model of this mechanism is provided in Supporting Information, including the piezocapacitive sensing model for force and the thermoresistive sensing model for temperature, which further verifies the intrinsically self-decoupled characteristics of RoboTac skin from a mathematical perspective.

**Figure 3:**
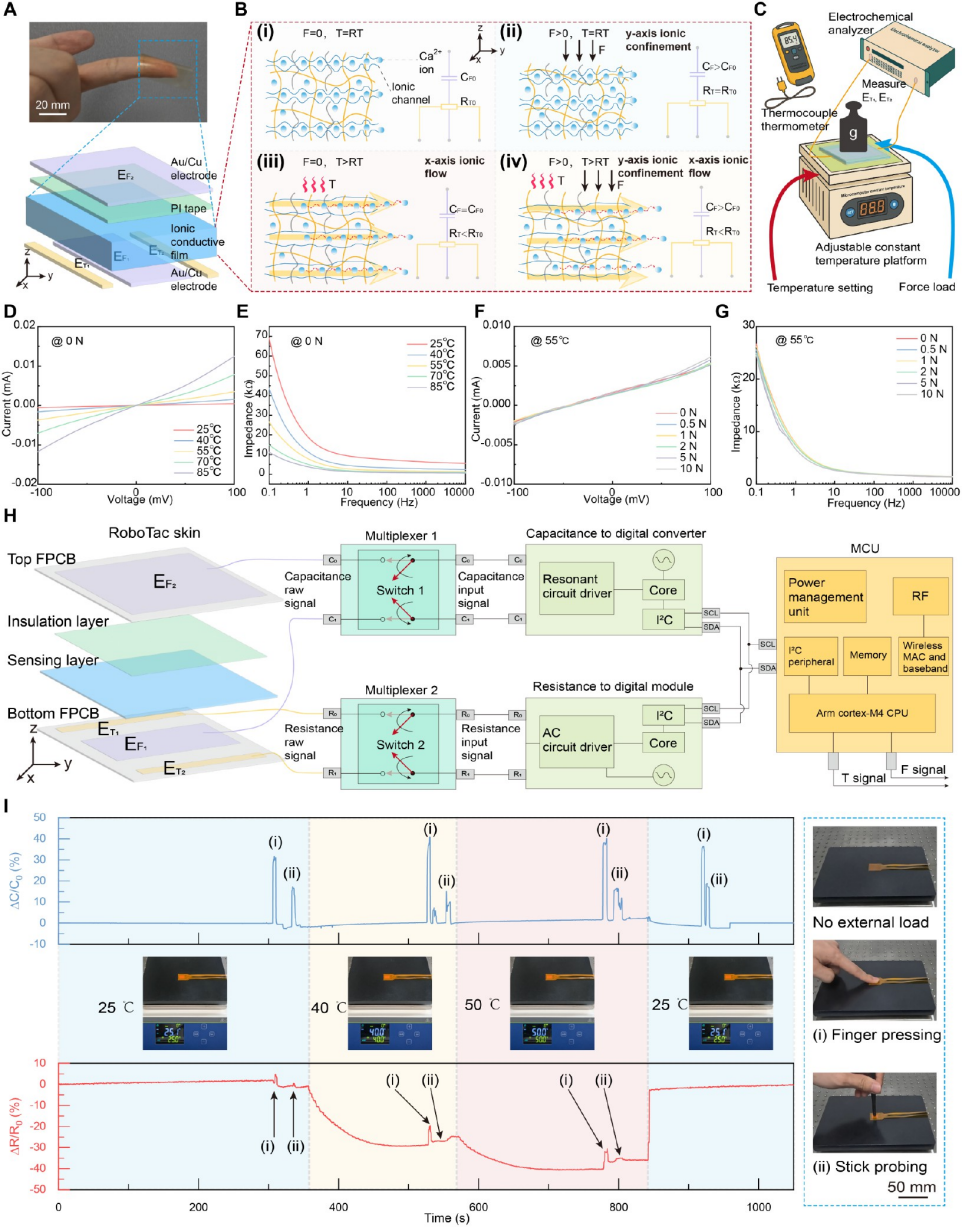
Principle of intrinsically self-decoupled sensing of RoboTac skin. (A) Optical photo of the ionic conductive film and illustration of RoboTac skin. (B) Schematic illustration of the intrinsically self-decoupled sensing mechanism. (C) Experimental setup for verification of the intrinsically self-decoupled sensing. (D) I–V characteristics of RoboTac skin without force load under different temperature conditions. (E) EIS characteristics of RoboTac skin without force load under different temperature conditions. (F) I–V characteristics of RoboTac skin with various force loads at 55 °C. (G) EIS characteristics of RoboTac skin with various force loads at 55 °C. (H) Data acquisition and processing system design for simultaneous measurement of force and temperature. (I) Experiment for validation of the intrinsically self-decoupled sensing.

To further validate the intrinsically self-decoupled dual-modal sensing capability of RoboTac skin, its electrochemical characteristics were examined using the experimental platform shown in Figure 3C. An adjustable constant temperature platform was employed to provide controlled thermal conditions, with a thermocouple thermometer for calibration, while weights were used to apply external forces. Then, an electrochemical analyzer measured the I–V curves and impedance characteristics between electrodes E_T1_ and E_T2_. Figure 3D and E show the I–V curves and EIS characteristics, respectively, under varying temperatures (25 °C, 40 °C, 55 °C, 70 °C, and 85 °C) without external force. As shown in Figure 3D, RoboTac skin exhibits a well-defined linear current–voltage relationship, and the slope of the I–V curve increases with temperature, rising from 0.0001% at 25 °C to 0.1% at 85 °C. This demonstrates an NTC sensing behavior. Consistently, the impedance–frequency plots in Figure 3E reveal a decrease in impedance with increasing temperature, further confirming its temperature sensitivity. Figure 3F and G present the results under various external forces (0, 0.5, 1, 2, 5, and 10 N) at a constant temperature of 55 °C. The I–V curves in Figure 3F nearly overlap, indicating that mechanical loading has only a limited influence on the temperature-sensing channel. Similarly, the impedance–frequency characteristics in Figure 3G show no significant changes, supporting the decoupling of temperature sensing from mechanical interference. This selective response arises because force sensing in RoboTac skin is primarily governed by compression-induced capacitance changes, while temperature variations produce only a small change in the capacitance channel over the calibrated range. As a result, thermal interference in the force-sensing channel is effectively suppressed. These findings further confirm the intrinsically self-decoupled nature of RoboTac skin, highlighting its potential for practical robotic applications.

Based on the intrinsically self-decoupled sensing capability of RoboTac skin, we developed a dual-modal data acquisition and processing system for simultaneous force and temperature measurement. As shown in Figure 3H, the system comprises two primary subsystems: a sensor interface and a data processing unit. RoboTac skin integrates multiple functional layers, including resistance-based temperature sensing electrodes (E_T1_, E_T2_), a capacitance-based force sensing electrode (E_F1_), and a top-layer floating electrode (E_F2_). To prevent signal interference between the two AC-excited measurement modalities, two analog multiplexers selectively switch among the sensor electrodes (E_F1_ and E_F2_ for multiplexer 1; E_T1_ and E_T2_ for multiplexer 2). In this configuration, the selected signals are routed either to a capacitance-to-digital converter (FDC module) for force sensing or to a resistance-to-digital converter (LCR module) for temperature sensing. The digitized data are then transmitted to an onboard microcontroller unit (MCU) via the I^2^C protocol for further processing (Figure S6). This fully integrated design enables real-time, high-fidelity acquisition of dual-modal tactile signals while maintaining desired scalability and modularity.

A verification experiment was conducted to further confirm the intrinsically self-decoupled dual-modal sensing capability of RoboTac skin (Movie S4 and Figure S7). As shown in Figure 3I, the skin was mounted on an adjustable constant temperature platform and subjected to two types of mechanical stimuli: finger pressing (Figure 3I (i)) and stick probing (Figure 3I (ii)). From 0 to 300 s, the platform was maintained at room temperature (RT) without active heating, and both capacitance and resistance remained at their initial values. At 305 s, finger pressing and stick probing were applied sequentially, producing capacitance variation rates of ∼30% and ∼15%, respectively, while the corresponding resistance variations were ∼5% and ∼0.5%. At 355 s, the platform temperature was raised to 40 °C. During the heating phase (360–480 s), capacitance remained nearly constant, whereas resistance gradually decreased to approximately −30%. After the temperature stabilized, finger pressing and stick probing at 528 s yielded capacitance changes of ∼40% and ∼10% and resistance changes of approximately −20% and −30%, respectively. The platform temperature then increased to 50 °C at 572 s. In this second heating phase, capacitance again remained unchanged, while resistance decreased further to around −40%. After stabilizing at 50 °C (778 s), mechanical stimuli produced response patterns consistent with those at 40 °C. Finally, at 845 s, the skin was transferred to another platform held at room temperature, and at 917 s, the same stimuli were applied, producing capacitance responses similar to those at elevated temperatures, while resistance returned to 0%. These results not only demonstrate the stable and repeatable behavior of both capacitive and resistive outputs, but also confirm that force and temperature stimuli can be independently measured, thereby validating the intrinsically self-decoupled sensing capability of RoboTac skin and highlighting its potential to enable human-like tactile perception in robotic systems.

### Performance of RoboTac Skin

To investigate the sensing performance of RoboTac skin, a calibration platform was constructed as shown in Figure 4A. A universal tensile machine equipped with an adjustable constant temperature platform was used to independently apply mechanical force and thermal variation. The platform, positioned on the compression side of the tensile machine, held the RoboTac skin directly beneath the compression axis (Figure S8), enabling temperature calibration under various mechanical loads and force calibration under different thermal conditions. The RoboTac skin was connected via FPC to a compact signal processing board with a stacked architecture consisting of a main board and a bottom board. The main board integrated a signal selection module and a capacitance measurement module, while the bottom board functioned as an LCR module. After signal conditioning and processing, capacitance and resistance data were transmitted through a USB cable to a PC for recording and analysis. This configuration allowed systematic calibration of both force and temperature sensing performance under controlled experimental conditions.

**Figure 4:**
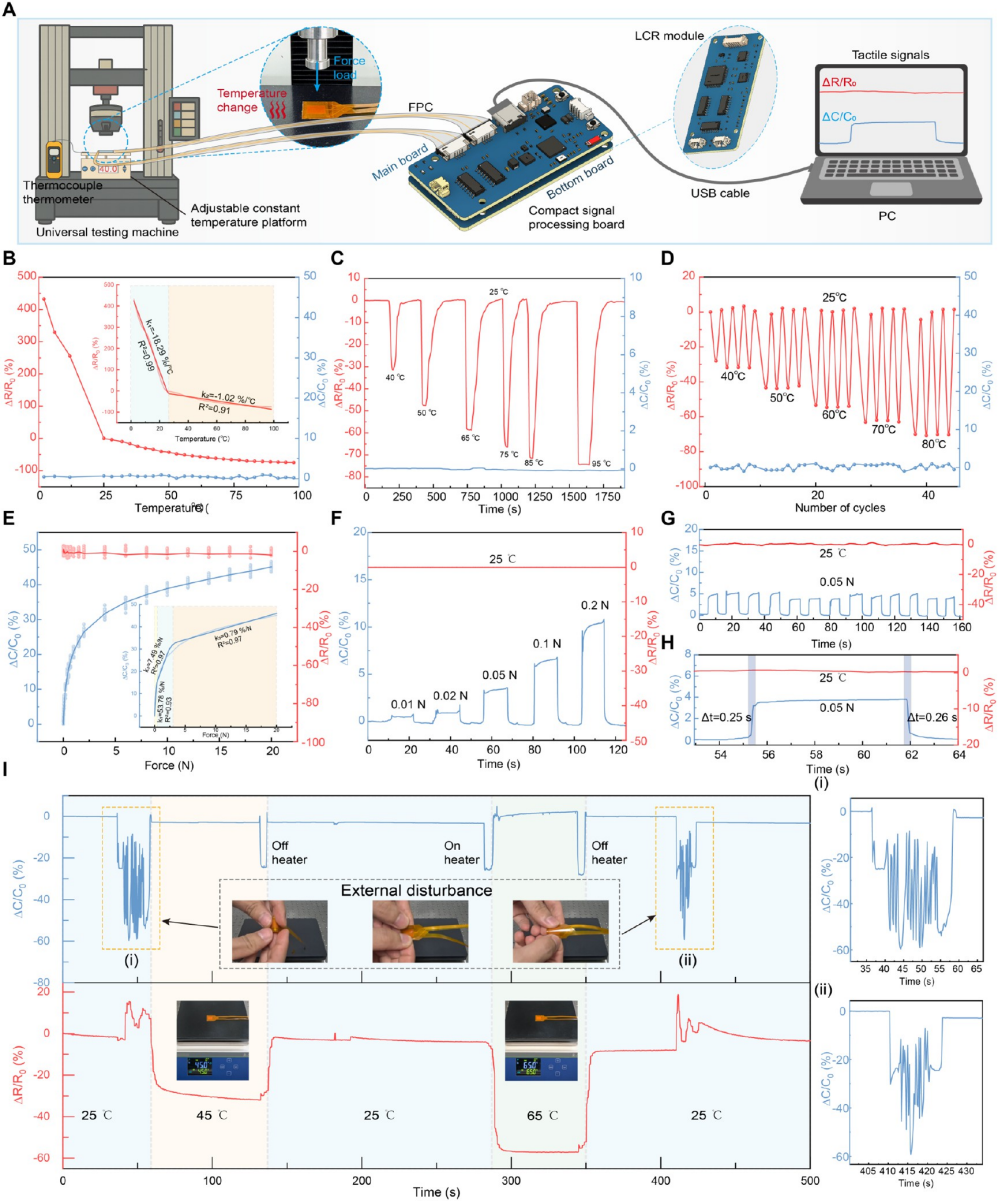
Intrinsically self-decoupled sensing performance of RoboTac skin. (A) Dual-modal experimental platform setup for sensing performance characterization. (B) Relationship between RoboTac skin’s response (resistance change rate ΔR*/*R_0_ and capacitance change rate ΔC*/*C_0_) and temperature. (C) Dynamic response of RoboTac skin to varied temperatures. (D) RoboTac skin’s response when thermal loads were applied and removed repeatedly. (E) Relationship between RoboTac skin’s response and force. (F) Dynamic response of RoboTac skin to varied forces. (G) RoboTac skin’s response when force loads were applied and removed repeatedly. (H) Time-resolved response of RoboTac skin to force load. (I) Dynamic response of RoboTac skin when an external disturbance is applied.

Thus, using the constructed calibration platform, the temperature and force sensing performances of RoboTac skin were evaluated independently. The sensing characteristics were obtained using the fully assembled device under controlled laboratory conditions, with normal compression applied within the active sensing area and stable contact with the loading or heating surface. As shown in Figure 4B, temperature sensing was characterized by recording both resistance and capacitance at each temperature point. The temperature was varied from 25 to 98 °C in 3 °C increments using the adjustable constant temperature platform, while additional measurements at 2 °C, 6 °C, and 12 °C were performed with an ice–water bath. Across the full range (2 °C-98 °C), the capacitance variation remained below 0.95% (maximum at 86 °C), confirming the decoupling of force sensing from temperature sensing. In the low-temperature range (2–25 °C), the resistance response showed high linearity (R^2^ = 0.99) and high sensitivity (−18.29%/°C), while in the high-temperature range (25 °C-98 °C) it maintained acceptable linearity (R^2^ = 0.91) with a sensitivity of −1.02%/°C. As shown in Figure 4C, dynamic temperature sensing was further assessed by monitoring the resistance response to rapid changes from room temperature to 40 °C, 50 °C, 65 °C, 75 °C, 85 °C, and 95 °C (heat transfer simulation in Figure S10). RoboTac skin accurately and promptly tracked temperature changes and consistently returned to its initial resistance when the platform was reset to RT. Throughout these tests, capacitance remained nearly constant, confirming robust dynamic temperature sensing performance with effective decoupling from force input. Repeatability was examined by cycling the platform between room temperature and target temperatures of 40 °C, 50 °C, 60 °C, 70 °C, and 80 °C, each repeated four times (Figure 4D). The resistance changes (∼30%, ∼40%, ∼50%, ∼60%, and ∼70%, respectively) closely matched the initial calibration curve with minimal fluctuations (Figure S9A, less than 0.8%) while capacitance variation remained negligible throughout (Figure S9B, ∼0.59%), confirming excellent repeatability, stability, and reliable decoupling of force sensing from temperature variation.

The force sensing performance of RoboTac skin was evaluated by measuring its capacitive responses to varying mechanical stimuli under different thermal conditions. As shown in Figure S11, the temperature was controlled using an adjustable constant temperature platform, starting from 25 °C and increasing in 5 °C increments up to 80 °C, yielding 12 distinct temperature settings. Forces from 0 to 20 N were applied using a universal testing machine. In the 2–20 N range, measurements were taken at 2 N intervals, whereas in the 0–2 N range, higher-resolution measurements were performed at 0.01, 0.02, 0.05, 0.1, 0.2, 0.3, 0.5, and 1.2 N. Capacitive and resistive responses under all temperature conditions were analyzed in terms of mean and standard deviation. As illustrated in Figure 4E, the normalized capacitance variation displayed distinct sensitivities across different force ranges: 53.78%/N (R^2^ = 0.93) for 0–0.4 N, 7.49%/N (R^2^ = 0.97) for 0.4–2 N, and 0.79%/N (R^2^ = 0.97) for 2–20 N. Across the full 0–20 N range, the mean of standard deviations in normalized capacitance was below 1% (Figure S12A), indicating minimal temperature influence and effective decoupling of thermal and mechanical sensing. Temperature-induced variation remained negligible throughout testing (Figure S12B), confirming the independence of temperature sensing from applied forces. Beyond temperature stability, RoboTac skin demonstrated high resolution in force sensing. As shown in Figure 4F, it reliably detected forces as small as 0.01 N, with dynamic responses matching the established sensitivity curves. This capability enables the detection of minute forces, which is crucial for interaction with delicate objects.

To quantify the mutual interference between the two sensing channels, we performed a full-scale normalized cross-sensitivity analysis using the complete force and temperature calibration data sets (Figure S13). The maximum temperature-induced capacitance variation over 2–98 °C was 0.96%, whereas the full-scale force-induced capacitance response over 0–20 N was 45.23%, corresponding to a temperature-to-force crosstalk ratio of 2.12%. Conversely, the maximum force-induced resistance variation was 5.83%, compared with a full-scale temperature-induced resistance response of 507.29%, yielding a force-to-temperature crosstalk ratio of 1.15%. In addition, the force-capacitance responses measured from 25 to 80 °C exhibited a mean standard deviation of 1.019% and a maximum standard deviation of 1.48% across the nonzero force points. Furthermore, Figure 4G demonstrates its repeatability under repeated loading: when subjected to a 0.05 N force for 15 cycles (110 cycles in Movie S5 and Figure S14), the capacitance variation consistently remained around 4%, and the resistance variation stayed close to zero, indicating desired stability and repeatability. RoboTac skin also exhibited a fast response time of approximately 0.25 s, as shown in Figure 4H. To further evaluate the fatigue characteristics of RoboTac skin, cyclic loading–unloading tests were extended to 2000 cycles at room temperature and 50 °C. As shown in Figure S15, the capacitance channel, which serves as the primary mechanical output, exhibited repeatable cyclic responses throughout the tests, and the response waveforms in the final cycles were comparable to those in the initial cycles. The mean peak capacitance variations were approximately 9.03% at room temperature and 8.94% at 50 °C, indicating that the cyclic force response was minimally affected by temperature. For the resistance channel, the mean normalized resistance levels were approximately −1.01% at room temperature and −49.39% at 50 °C. The latter represents the expected temperature-induced baseline offset relative to the initial room-temperature resistance, rather than cycle-induced drift. Within each fixed-temperature cycling block, the resistance signal remained stable and showed no obvious periodic modulation induced by repeated mechanical loading under the tested conditions. After the cyclic tests, visual inspection and manual bending and twisting confirmed that the internal PI-tape bonding remained intact, with no visible delamination, edge lifting, or tape detachment (Figure S16, Movie S12). These results demonstrate that the cross-channel responses remain substantially smaller than the corresponding principal responses within the calibrated operating ranges.

In addition, baseline recovery was further quantified using the repeated thermal and mechanical cycling data sets (Figure S17). After heating–cooling cycles, the resistance and capacitance baseline recovery errors were 1.78 and 0.70 percentage points, respectively. During 2000 mechanical cycles, the capacitance baseline recovery errors were 0.17 percentage points at room temperature and 0.27 percentage points at 50 °C, while the corresponding resistance errors were 0.11 and 0.13 percentage points. These results indicate reproducible baseline recovery with small residual offsets under repeated thermal and mechanical stimuli. These findings confirm that RoboTac skin enables high-performance force and temperature sensing with intrinsically selective transduction and ultralow experimentally quantified crosstalk between the two modalities.

Beyond characterizing the intrinsically self-decoupled sensing of RoboTac skin, its robustness against external disturbances was also evaluated (Movie S6 and Figure S18). As shown in Figure 4I, manual deformations such as folding and compression were applied under different thermal conditions (45 and 65 °C) to assess interference resistance. Under ambient conditions (25 °C), capacitance fluctuations remained at the noise level (Figure 4I (i)), while resistance exhibited moderate variation. After removal of the disturbance and placement on a 45 °C platform, capacitance remained nearly unchanged, whereas resistance variation increased to ∼35%. Returning to RT caused resistance variation to drop back to 0%, with capacitance still stable. At 65 °C, capacitance again remained steady, while resistance variation increased to ∼60%. Following a second round of manual disturbances at room temperature, both capacitance and resistance variations were negligible, indicating strong resistance to mechanical interference. These results confirm the robustness of RoboTac skin, demonstrating its stable performance under dynamic and unpredictable conditions. Taken together, the comprehensive evaluation establishes that RoboTac skin offers self-decoupled dual-mode sensing of force and temperature (specifications in Table S3), with high resolution, repeatability, and robustness, making it highly suitable for tactile sensing in robotic applications.

### Demonstration of RoboTac Skin on Object Perception

For humans, tactile perception is a fundamental sensory modality that plays a critical role in interactions with physical objects. Previous experiments have confirmed that RoboTac skin possesses intrinsically self-decoupled dual-modal sensing, enabling robots to acquire human-like tactile, and thereby enhancing their perception of objects in specific scenarios. As shown in Figure 5A, when RoboTac skin-equipped robotic gripper grasps different objects, the signal processor converts the capacitive and resistive inputs into force and temperature signals, respectively, via dual-modal calibration curves. These signals are then analyzed by a convolutional fully connected classification neural network (CNNFCNet) to identify the grasped object. As shown in Figure 5B, the RoboTac skin and the compact signal processing module were affixed to the inner and outer surfaces of one side of the robotic parallel gripper using a VHB tape. The robot then performed in situ grasping of a variety of common daily objects with different sizes, shapes, and stiffnesses (glue stick, test tube, disinfectant, tape, yarn ball, lubricant, phial, keycap, sausage, sugar cube, chip, and litchi). For each object, 50 trials were conducted, yielding a total of 600 data sets; representative signals from each object type are shown in Figure S19. The CNNFCNet model (Figure 5C) comprises two convolutional layers, two max-pooling layers, three fully connected layers, and a SoftMax classifier, enabling effective processing and classification of raw tactile signals. The classification confusion matrix (Figure 5D) demonstrates high self-state recognition accuracy (overall accuracy 95.1%), with most states exhibiting perfect discrimination accuracy of 100%. This accurate and reliable perceptual ability enables robots to conduct challenging tasks in versatile scenarios, such as assembly line grabbing, automated experiments, and medical healthcare.

**Figure 5:**
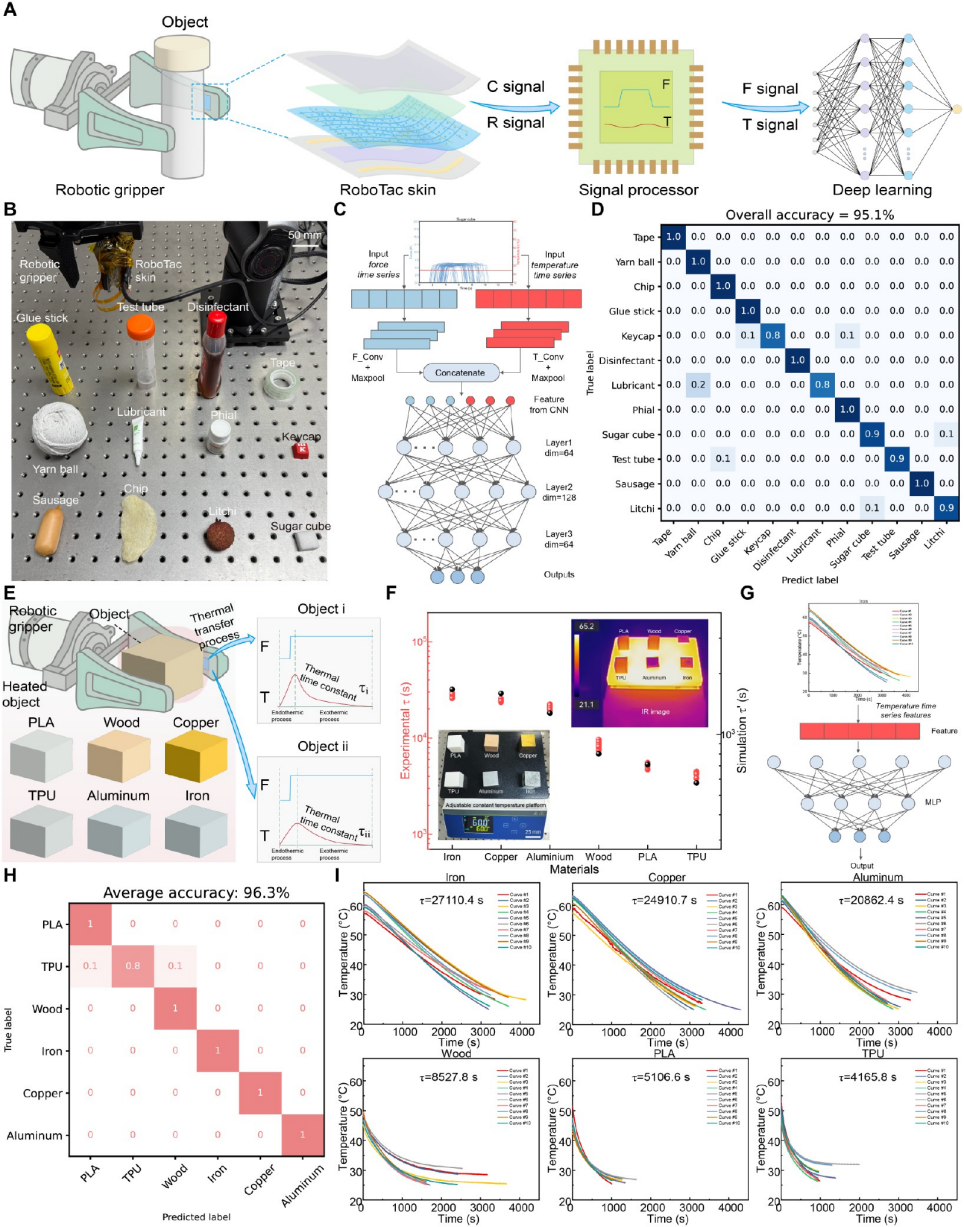
Demonstration of RoboTac skin on object perception. (A) The mechanism by which the robot perceived and classified objects through the RoboTac skin. (B) The photo of the robot equipped with RoboTac skin on its gripper alongside the objects (glue stick, test tube, disinfectant, tape, yarn ball, lubricant, phial, keycap, sausage, sugar cube, chip, and litchi) to be grasped. (C) The deep learning framework is based on dual-modal signals from RoboTac skin for object classification. (D) The confusion matrix of the classification results of the grasped objects by the robot. (E) The robot identifies the material of objects using RoboTac skin, based on the temperature signal for a transient heat conduction method. (F) The experimental time constant (τ) and simulation time constant (τ’) of the transient thermal decay process for different materials. (G) The deep learning framework is based on exponential decay curves of temperature from RoboTac skin for material classification. (H) The confusion matrix of the classification results of the materials by the robot. (I) Ten real-time temperature responses of the transient thermal decay process for different materials (iron, copper, aluminum, wood, PLA, and TPU).

Material classification has long been a challenge in robotic object perception, as relying solely on vision or force sensing often fails to distinguish between objects with identical shapes or similar appearances. The transient thermal conduction process refers to the change in an object’s temperature from a transient state to a steady state. For a single object that satisfies the lumped system assumption, the temperature variation curve from an initial high-temperature state to a steady state (e.g., RT) is termed the exponential decay curve of temperature. The fitted thermal time constant (τ) reflects the combined effects of the object’s thermal properties and the heat-transfer conditions at the object-sensor interface. Under controlled and consistent geometry, heating, contact, and environmental conditions, differences in τ can therefore be used as discriminative features for material identification. Thus, the material characteristics can be inferred by calculating the time constant from a measured temperature decay curve. As shown in Figure 5E, we conducted experiments using a robotic gripper equipped with RoboTac skin, which provides intrinsically self-decoupled dual-modal sensing capability, to perform material identification.

The test samples comprised six objects with identical volume and surface area but made of different materials: PLA, wood, copper, TPU, aluminum, and iron (main thermal parameters in Table S6). To minimize the influence of extrinsic variables, all samples were tested using the same heating platform, RoboTac skin, robotic gripper, nominal contact position, and grasping procedure under the same environment. The exponential decay curve of temperature measured via RoboTac skin was used to determine the material of each object. As shown in Figure 5F and Movie S7, the six objects were first heated to a constant temperature on the same adjustable constant temperature platform (60 °C) for a period of time, then grasped by the robotic gripper, which recorded their exponential decay curves of temperature (Figure 5I) via the RoboTac skin, including iron, copper, aluminum, wood, PLA, and TPU. The robotic gripper performed 10 measurements for each material, and the average time constants (in descending order) were iron (27,110 s), copper (24,910 s), aluminum (20,862 s), wood (8527 s), PLA (5106 s), and TPU (4165 s). These values, along with the simulated time constants (simulation in Figure S21), are plotted in Figure 5F. Notably, the initial temperatures of each object were different due to their different heat capacities, corresponding to the simulation in Figure S20. The results show that the ranking of experimental time constants is consistent with the simulated results, namely iron, copper, aluminum, wood, PLA, and TPU (in decreasing order), confirming that the time constant obtained from the exponential decay curve of temperature serves as a reliable indicator for material identification.

Furthermore, to enable object classification based on exponential decay curves of temperature, we designed a set of features as classification criteria in Figure 5G, including the curve’s initial value, steady-state value, and their difference; τ and goodness of fit obtained from fitting an exponential decay model; and the ratio of the slopes of the initial and final segments. These features were fed into a three-layer neural network built with a multilayer perceptron (MLP), in which ReLU activation functions and a Dropout mechanism were incorporated to enhance the model’s nonlinear representation capability and suppress overfitting. The classification confusion matrix (Figure 5H) demonstrates high self-state recognition accuracy (overall accuracy 96.3%), with most states exhibiting perfect discrimination accuracy of 100%. These findings further indicate that the intrinsically self-decoupled dual-modal sensing capability of RoboTac skin can significantly enhance robotic tactile sensing, enabling both object classification and materials identification for challenging cases, thereby laying the foundation for highly generalized object perception in daily scenarios.

### Demonstration of RoboTac Skin on Special Tasks

Special tasks demand a high level of adaptability from robots, and the deployment of RoboTac skin significantly enhances their dual-modal sensing capability for both force and temperature signals, thereby improving robots’ performance in such scenarios. As shown in Figure 6A and Movie S8, the robot equipped with the RoboTac skin exhibits dual-modal sensing capabilities, enabling precise manipulation and transfer of objects containing liquids at different temperatures. As illustrated in Figure 6B, the robot transferred RT water from a table to a platform. During the approach stage (0–23 s), the robot approached the target (F = 0 N, T = RT); when it grasped the cup at 23 s, the force signal changed while the temperature signal remained at RT. During the move to the target place stage (23–37 s), the force signal increased from ∼5 N to ∼10 N due to changes in gripping force during movement, while the temperature signal remained constant. After placing the cup (37–49 s), the force signal dropped to 0 N with no change in temperature. In the cold water transfer task (Figure 6C), the approach stage (0–18 s) showed similar force and temperature signals (F = 0 N, T = RT) as in the RT case. During grasping and moving (18–32 s), the force signal increased from ∼5 N to ∼10 N, and the temperature dropped from ∼25 °C to ∼15 °C. After the robot placed the cold water and returned to the initial position (32–53 s), the force signal dropped to 0 N, and the temperature began to recover toward RT. Figure 6D illustrates the process of transferring hot water. Similarly, during the approach stage (0–28 s), the force and temperature signals (F = 0 N, T = RT) were consistent with the previous cases. During the grasping and moving phase (28–65 s), the force signal rose from ∼5 N to ∼7.5 N, while the temperature gradually increased to 50 °C. Notably, sufficient contact duration was maintained during this phase to allow heat transfer from the object to RoboTac skin. The observed temperature evolution reflects the system-level transient heat-transfer process at the object-sensor interface rather than the sensor’s intrinsic electrical response time. After placing the hot water and returning to the initial position (65–77 s), the force signal dropped to 0 N, and the temperature signal gradually returned to RT. These experimental results further validate the intrinsically self-decoupled force and temperature sensing capability of RoboTac skin. By endowing the robot with human-like dual-modal perception, it demonstrates strong potential for performing object manipulation tasks under complex thermal and mechanical conditions.

**Figure 6:**
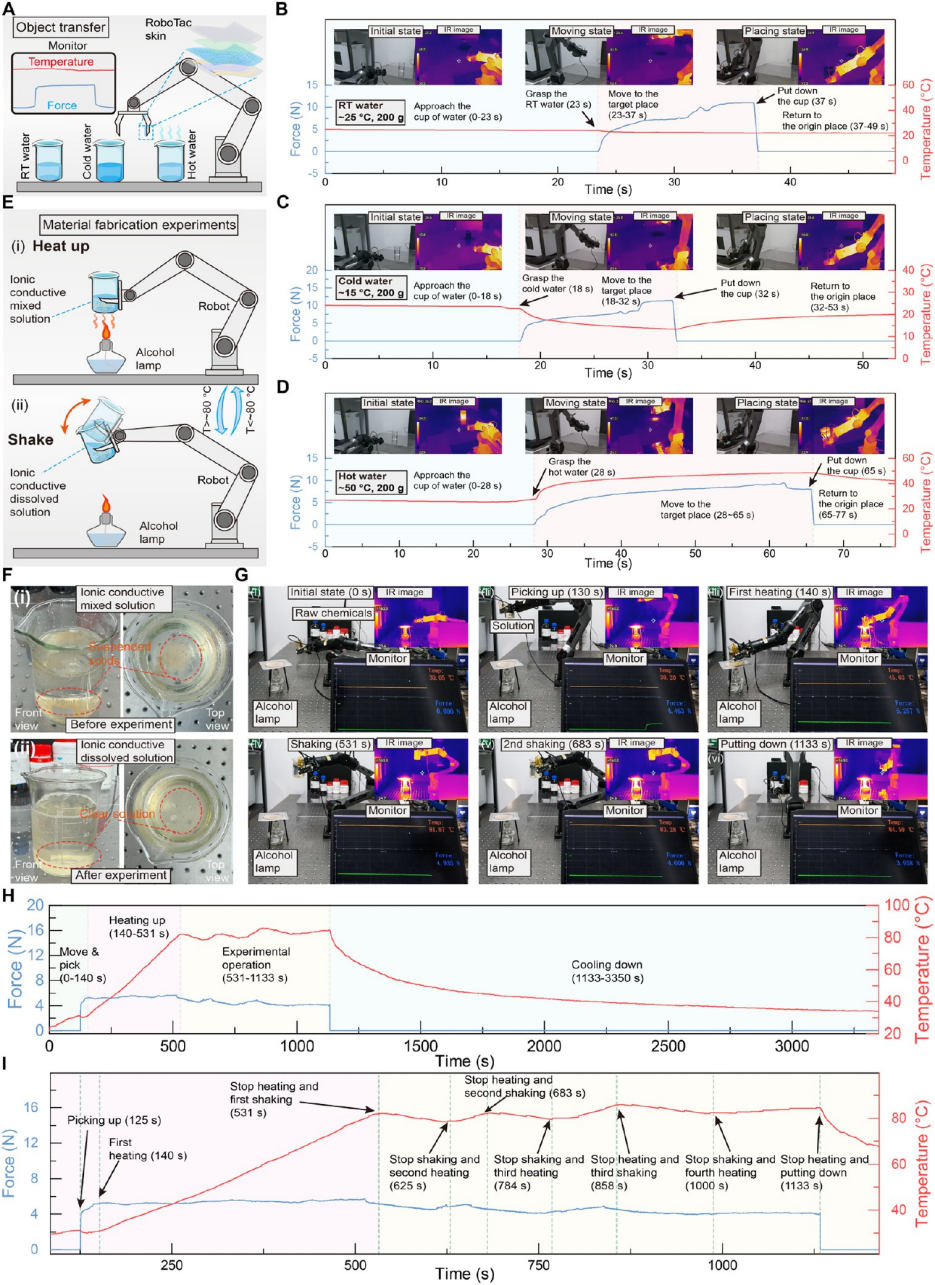
Demonstration of RoboTac skin on special tasks. (A) The robot transferred water with different conditions based on the dual-modal signals via RoboTac skin, including (B) room temperature water (∼25 °C, 200 g), (C) cold water (∼15 °C, 200 g), and (D) hot water (∼50 °C, 200 g). (E) The robot conducted an ionic conductive solution (precursor solution for ionic conductive film) based on the dual-modal signals via RoboTac skin. (F) Optical photos of the ionic conductive solution before and after the experiment conducted by the robot. (G) Key photos of the fabrication of the ionic conductive solution through the robot. (H) Real-time response of the dual-modal signals during (0–1133 s) and after (1133–3350 s) the experiment. (I) Real-time response of the dual-modal signals during the fabrication experiment (0–1133 s).

Automated material synthesis experiments in laboratory settings represent a type of special task in complex environments, requiring robots to possess highly capable sensing abilities, as such processes often involve varying force and temperature conditions. Here, the robot equipped with RoboTac skin was tasked with synthesizing the ionic conductive solution, which serves as the precursor to the ionic conductive film (Movie S9). The synthesis process involves heating and stirring to fully dissolve solutes in the solution and trigger the intended chemical reactions, which are decomposed into heating and shaking. As shown in Figure 6E (i), the robot placed the beaker containing the ionic conductive mixture onto an alcohol lamp for heating; once the temperature reached ∼80 °C, the robot proceeded to shake the beaker to accelerate the dissolution and reaction of the solutes (Figure 6E (ii)). These two steps were repeated cyclically until the solutes were fully dissolved to form the ionic conductive solution. Subsequently, this solution can be processed through heat-assisted evaporation to form the ionic conductive film.

As shown in Figure 6F (i), prior to the processing experiment, the solution formed by adding PEC, CHI, PEG, GLY, CaCl_2_, and citric acid into deionized water in a defined ratio appeared turbid with suspended particulates. In contrast, Figure 6F (ii) demonstrates that after the processing experiment, the solution became clear, with all suspended substances fully dissolved, resulting in the formation of the desired ionic conductive solution. The key steps of the experiment are illustrated in Figure 6G, and the dual-modal sensing signals recorded during the process are shown in Figure 6H and I. According to Figure 6H, the entire process can be divided into four stages: move and pick (0–140 s), heating up (140–531 s), experimental operation (531–1133 s), and cooling down (1133–3350 s). As shown in Figure 6G (i), when the robot was in the initial state (0 s), the force signal was 0 N and the temperature signal was RT. At 125 s, after the robot moved to the solution and completed the grasp (Figure 6G (ii)), the force signal increased to ∼5 N while the temperature remained at RT. At 140 s, the robot placed the solution above an alcohol lamp for the first heating (Figure 6G (iii), F = ∼5 N, T gradually increasing). After ∼390 s of heating (F = ∼5 N), the solution reached ∼80 °C at 531 s. As shown in Figure 6G (iv), the robot then lifted the solution vertically to stop heating and initiated the first shaking (F remained constant and T gradually decreased). When the temperature dropped below ∼80 °C (625 s), the robot stopped shaking and began a second heating (F remained constant and T gradually increased). When the temperature exceeded ∼80 °C (683 s), the robot stopped heating and shook second (Figure 6G (v)), where the temperature gradually decreased while the force signal remained unchanged. This was then followed by two more heating phases (at 784 and 1000 s) and one additional shaking (at 858 s), with corresponding fluctuations in temperature while the force signal remained stable. At 1133 s, as shown in Figure 6G (vi), the robot ended the experimental operation and transferred the solution to the experimental table, resulting in the force signal dropping to 0 N. The temperature then gradually decreased, reaching ∼30 °C after ∼2300 s.

Following the robot’s operation, the solutes in the ionic conductive solution were fully dissolved, forming a homogeneously stirred and chemically reacted precursor solution that can be used for the subsequent synthesis of the ionic conductive film. The results of the robotic heating and stirring experiment further validate the intrinsically self-decoupled dual-modal sensing capability of RoboTac skin, which enables stable and reliable perception during complicated tasks, thereby demonstrating environmental adaptability and providing a foundation for laboratory automation. By endowing the robot with human-like dual-modal perception, RoboTac skin enables precise interaction with force–temperature coupled objects commonly encountered in specialized scenarios, paving the way for fully automated robotic material synthesis in laboratory settings.

### Demonstration of RoboTac Skin on Human–Robot Interaction

As robots increasingly integrate into daily human life, human-robot interaction has become ever more critical. A fundamental prerequisite for effective human-robot interaction is the robot’s ability to achieve human-like tactile sensing, enabling it to perceive objects exhibiting coupled force and temperature features commonly encountered in daily environments. Here, we demonstrate human-robot interaction tasks empowered by the intrinsically self-decoupled dual-modal sensing capability of RoboTac skin. As shown in Figure 7A, a robot equipped with RoboTac skin performs a tea-brewing service task for a human, which consists of two steps: brew tea (Figure 7A (i)) and deliver to humans (Figure 7A (ii)). RoboTac skin provides essential force and temperature signals that allow the robot to brew tea with hot water and deliver the prepared tea to the human.

**Figure 7:**
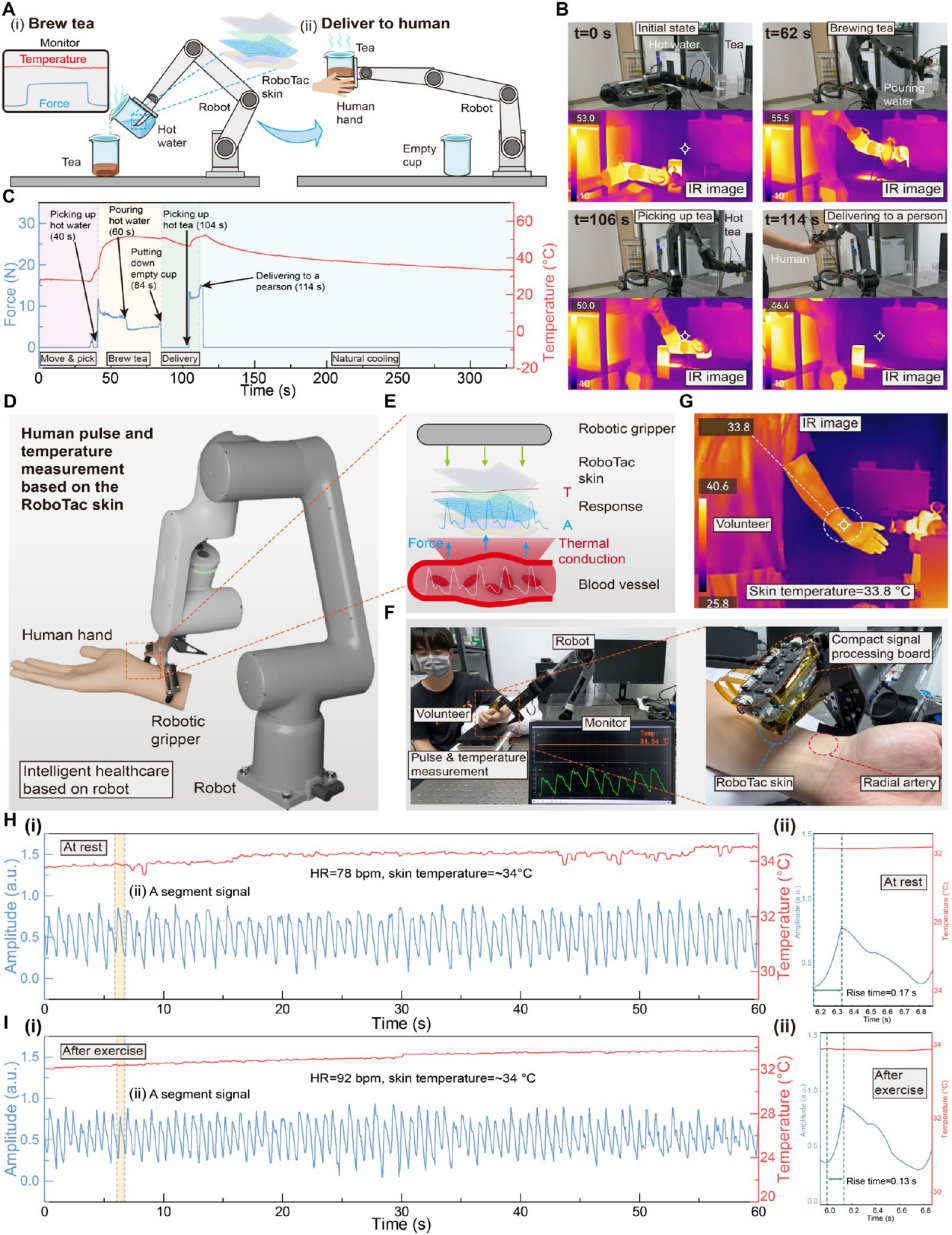
Demonstration of RoboTac skin on human-robot interaction. (A) The robot brewed tea for humans based on dual-modal signals sensed by RoboTac skin. (B) Key photos of brewing tea for a person through the robot. (C) Real-time response of the dual-modal signals during (0–114 s) and after (114–330 s) the experiment. (D) The robot performs pulse diagnosis for humans using bimodal signals sensed by RoboTac skin, simultaneously measuring pulse and skin temperature. (E) The principle of measuring radial artery pulse and surface body temperature using RoboTac skin. (F) Photos of the robot measuring a volunteer’s wrist pulse and surface body temperature using RoboTac skin. (G) The skin temperature at the wrist region of the human body is shown in the IR image. (H) (i) The pulse and body temperature of the volunteer at rest (period ∼60 s) as perceived by the robot, along with (ii) the corresponding signals of a single pulse cycle (6.1–6.8 s). (I) (i) The pulse and body temperature of the volunteer at rest (period ∼60 s) as perceived by the robot, along with (ii) the corresponding signals of a single pulse cycle (6–6.7 s).

As illustrated in Figure 7B and Movie S10, the robot was in the initial state (0 s), with a cup of hot water and an empty cup containing tea leaves placed on the table. The robot then picked up the cup of hot water and poured (62 s) it into the teacup for brewing. Subsequently, it picked up the brewed teacup at 106 s and delivered it to the human at 114 s. The dual-modal sensing signals provided by RoboTac skin during the task are shown in Figure 7C. During the move and pick phase (0–40 s), the robot obtained dual-modal signals (F = 0 N, T = RT). Upon grasping the hot water cup at 40 s, the temperature began to rise and reaches ∼50 °C, while the force signal increased sharply to ∼7.5 N. Notably, at around 38 s, the sensed temperature already started to increase and a small spike appeared in the force signal, which is attributed to the gripper approaching and slightly bumping into the cup wall. During the tea-brewing stage (60 s), the force gradually decreased to ∼5 N, and the temperature signal stabilized at ∼50 °C. The robot then moved the now-empty cup back to its initial position and released it (84 s), with both force and temperature signals remaining unchanged. At 104 s, the robot moved to the teacup and completed the grasp, registering a force of ∼12 N and a temperature that, having decreased to ∼46 °C due to heat dissipation from the gripper, rises again to ∼50 °C. During delivery to the human (114 s), it is noteworthy that a step-like increase in force occurred at ∼111 s, because the human hand encountered and gripped the teacup. After the task is completed (after 114 s), the robot returned to its initial state, the temperature naturally decayed, and the force dropped back to 0 N. These results further verify the intrinsically self-decoupled dual-modal sensing capability of RoboTac skin, which enables the robot to reliably complete complex tea-brewing tasks for humans. This serves as a solid foundation for robots to perform assistive service tasks in close collaboration with humans.

In addition, with the advent of population aging, healthcare-related robotic tasks are becoming increasingly important. Pulse and body temperature, as two of the most fundamental physiological indicators, have long been focal points of health monitoring. However, enabling robots to simultaneously monitor human pulse and body temperature remains a significant challenge. Here, as illustrated in Figure 7D, we demonstrate that the intrinsically self-decoupled dual-modal sensing of RoboTac skin endows robots with the capability to simultaneously monitor both parameters. Figure 7E illustrates the working principle, where the robot’s gripper presses on the region of the wrist artery so that the pulsatile blood flow exerts rhythmic forces and the associated heat is conducted to the RoboTac skin, enabling simultaneous sensing of pulse and temperature. As RoboTac skin can sense both force and temperature concurrently, it enables the measurement of both pulse and body temperature. As shown in Figure 7F and Movie S11, we demonstrated a task in which the robot measures the pulse and temperature of a volunteer. With RoboTac skin deployed on the robot gripper, the robot can continuously monitor the volunteer’s pulse and body temperature in real time with skin temperature ∼34 °C (Figure 7G) as a benchmark.

First, the robot measured the volunteer’s pulse and body temperature under resting conditions. As shown in Figure 7H (i), the measured signals (∼60 s) demonstrate good periodic consistency in the pulse data and stable, steady readings for the temperature. The results indicate a resting heart rate (HR) of 78 bpm and a body temperature of ∼34 °C. Figure 7H (ii) shows a segment of the resting-state pulse signal, which exhibits a rise time of 0.17 s and distinct pulse waveform features. In addition, the robot measured the volunteers’ pulses and body temperature after exercise. As shown in Figure 7I (i), the postexercise pulse signal also exhibits strong periodic consistency, and the temperature signal remains stable, with the measured HR reaching 92 bpm and the body temperature maintained at ∼34 °C. Figure 7I (ii) presents a segment of the postexercise pulse signal, which has a rise time of 0.13 s and still shows clear pulse features. These experimental results demonstrate that the intrinsically self-decoupled dual-modal sensing of RoboTac skin enables the robot to capture both distinct pulse signals and stable temperature readings. The observed acceleration in pulse after exercise aligns with the expected physiological response and further validates the effectiveness of robots equipped with RoboTac skin in medical healthcare scenarios. This highlights the potential of equipping medical and caregiving robots with human-like tactile sensing capabilities.

## Conclusions

Tactile perception plays a pivotal role in enabling robots to interact intelligently and safely with their environment. While prior dual-modal tactile sensors have achieved progress in sensing force and temperature simultaneously, they typically suffer from signal crosstalk, complex architecture, and limited robustness under real-world conditions. These limitations significantly hinder their practical integration into robotic systems, particularly those requiring stable and high-fidelity multimodal perception. In contrast, RoboTac skin developed in this work addresses these challenges by introducing a tactile sensing strategy with intrinsically self-decoupled force and temperature modalities via ionic conduction. Drawing inspiration from the structural and functional characteristics of human skin, RoboTac skin addresses these gaps with a novel sensing mechanism based on ion conduction and directional confinement, enabling intrinsically self-decoupled, robust dual-modal measurements with ultralow mutual interference. Here, “human-like tactile sensing” refers specifically to dual-modal force and temperature perception with intrinsic force–temperature self-decoupling. Other aspects of human touch, such as vibration, texture, pain, humidity, and neural encoding, are beyond the scope of this work. RoboTac skin employs a scalable four-layer sandwich architecture with a specially formulated, transparent, and biocompatible ionic conductive film that enables predominantly selective responses to mechanical and thermal inputs. Electrochemical and mechanical characterizations reveal that this film achieves an optimal balance between conductivity, mechanical resilience, and thermal responsiveness. Experimental results further confirm that RoboTac skin exhibits high sensitivity and repeatability in both temperature and force, with experimentally quantified full-scale crosstalk below 2.12% across a wide work range. Moreover, it demonstrates strong environmental robustness under folding, compression, and temperature variation, underscoring its reliability in dynamic manipulation scenarios. Benefiting from this intrinsically self-decoupled dual-modal sensing capability, RoboTac skin enables robots to acquire clean tactile information even under simultaneous mechanical and thermal interference. By integrating RoboTac skin, we demonstrated its capability to enhance robotic performance in object perception (object classification and material identification), specialized tasks (object transfer and laboratory automation), and human-robot interaction (assistive services and medical healthcare). This work is anticipated to advance robotic tactile sensing, improve the generalizability of robot-environment interactions, and stimulate further research in multimodal sensing, intelligent perception, and embodied robotics.

Nevertheless, some limitations remain and motivate future work. The current electrode design, while effective for decoupling, may require further miniaturization and array-level integration to enhance spatial resolution for high-resolution tactile mapping. In addition, although the ionic conductive film has demonstrated high performance under controlled conditions, long-term stability and repeatability in harsh environments (such as humid or biofluid-exposed environments) require further investigation. In the current device, the ionic conductive film is enclosed between the top and bottom FPCBs and sealed with PI tape, which effectively limits direct exposure to the ambient environment and suppresses moisture loss. Future work will focus on evaluating dehydration-induced signal drift and developing improved moisture-barrier encapsulation, extending architecture into flexible tactile arrays, integrating active switching or signal preprocessing, and combining with AI-based decoding frameworks to achieve intelligent perception. The key to scaling RoboTac skin into arrays lies in the design of electrode architectures and multiplexed readout circuits that maximize resolution while preserving sensitivity. Advanced manufacturing approaches may be leveraged to realize optimized array structures, while efficient addressing architectures will further enhance the overall sensing fidelity. In conclusion, this study presents a very simple yet highly effective dual-modal tactile skin with intrinsically self-decoupled force and temperature sensing. This work not only advances the fundamental understanding of multimodal tactile decoupling but also offers promising potential for diverse applications in dexterous manipulation, environmental interaction, and human-robot collaboration.

## Materials and Methods

### Materials

Pectin was supplied by Sigma-Aldrich Corporation. Chitosan (CHI), polyethylene glycol 400 (PEG400), and glycerol (GLY) with analytical reagents were purchased from Shanghai Macklin Biochemical Technology Co., Ltd. Citric acid and calcium chloride (CaCl_2_) were bought from Shanghai Aladdin Biochemical Technology Co., Ltd. Polyimide (Kapton) tape was obtained from 3 M Company. FPCB (polyimide substrate) was provided by JLC Technology Group. All solvents and chemicals were purchased from commercial sources unless otherwise noted.

### Preparation of RoboTac Skin

As shown in Figure 2A, a prepolymerized solution was prepared by dissolving pectin (0.2 g), chitosan (0.01 g), PEG400 (0.1 g), glycerol (0.1 g), CaCl_2_ (0.2 g), and citric acid (0.02 g) in deionized water (10 g). The mixture was stirred continuously in an 80 °C water bath for 2 h, followed by ultrasonic treatment for 1 h to remove air bubbles. Subsequently, the solution was heated at 80 °C for 2 h, after which the resulting ionic conductive film was cut into the desired shape.

Then, the ionic conductive film was mounted onto the bottom FPCB. A piece of PI tape was then adhered to the ionic conductive film as an insulation layer. Lastly, RoboTac skin was obtained by sealing the top FPCB on top of the insulation layer. After fabrication of the RoboTac skin, the FPCB was connected to the compact data processing board.

### Data Acquisition and Processing System Design and Programming

A dual-modal data acquisition and processing system was developed to capture tactile information from RoboTac skin. As illustrated in Figure 3H, RoboTac skin includes multiple functional layers, i.e., resistance-based temperature sensing electrodes (E_T1_, E_T2_) at the bottom layer, capacitance-based force sensing electrodes (E_F1_) at the bottom layer, and a top-layer capacitance-based force sensing electrode (E_F2_) at the top layer. To avoid interference between the two AC-excited measurement modalities, two analog multiplexers (CD74HC4052M) are employed to selectively switch between different sensor electrodes. The selected signals are routed either to a capacitance-to-digital converter (FDC2214) for force sensing or to an external commercial LCR module for temperature measurement via resistance sensing. All digitized data is transmitted via the I^2^ C protocol to an onboard microcontroller unit (MCU) for further processing.

To implement this architecture, we designed a compact wireless PCB measuring 30 × 63 mm, which was fabricated and assembled by JLC Technology (Figure S22). The core processing module is the ESP32-S3-WROOM-1 (Espressif Systems, Inc.), which is a high-performance microcontroller. Additional key components on the board include a USB-to-UART bridge (CH340N) for firmware flashing and serial communication, and a 4-pin FPC connector (AFA07-S04FCA-00, Jushuo Electric Company) to interface with tactile skin. The FDC2214 chip provides high-resolution, resonant-based capacitance measurement with up to four independently configurable input channels. The power subsystem is built around the AXP2101 (X-Powers), a multichannel power management IC that supports voltage sequencing and dynamic power control. To ensure clock stability for the MCU and peripheral modules, both a 40 MHz passive crystal oscillator (Yangxing Technology Company) and a 40 MHz active crystal oscillator (ClearType Systems Company) are deployed. Firmware development was carried out using the Arduino IDE (version 2.0.4). Sensor communication drivers and control logic were adapted from open-source libraries, including the Adafruit MLX90393 driver (MIT license) and the official Espressif SDK. The ESP32-S3MCU was programmed in UART boot mode. This integrated system enables real-time and high-fidelity acquisition of dual-modal signals with strong scalability and modularity for robotic applications.

### Verification of Self-Decoupled Sensing

Intrinsically self-decoupled sensing was verified through electrochemical experiments. The RoboTac skin was mounted on the adjustable constant temperature platform, and a thermocouple thermometer was employed to monitor the temperature of the platform. The force load was applied by using standard calibrated weights with equal base areas. Then, the electrochemical analyzer was connected to the electrodes E_T1_ and E_T2_ to obtain electrochemical characteristics data under conditions where either temperature (Figure 3C and D) or force (Figure 3E and F) is altered independently.

### Sensing Performance and Calibration of RoboTac Skin

RoboTac skin sensing performance was characterized by a universal testing machine (ZQ-990, Dongguan Zhiqu Precision Instruments Co., Ltd.) with an adjustable constant temperature platform on it (Figure 4A and Figure S8). The universal testing machine provided different force loads, while the adjustable constant temperature platform (LC-DB-XDA, Lichen Instrument Technology Co., Ltd.) provided the temperature change. After the RoboTac skin connected to the compact data processing board, a PC was able to obtain the signals of resistance and capacitance through the compact data processing board (LCR module for resistance and FDC module for capacitance). The dual-modal sensing performance was measured as temperature and force sensing performance, measuring one while the other is measured. The temperature sensing performance was measured as resistance during various temperatures by the adjustable constant temperature platform (step size 3 °C when above 25 °C) and ice water (2, 6, 12 °C when below 25 °C). The force sensing performance was measured as capacitance during different force loads at different temperature conditions (from 25 to 80 °C in 5 °C steps).

### Setup of the Demonstration

For all demonstrations in this work, RoboTac skin was fixed to the inner surface of the robotic gripper (PiPER, AgileX Robotics) using double-sided adhesive tape (VHB tape, 3M Company), while the compact signal processing board was mounted on the outer surface. RoboTac skin and the compact signal processing board were connected via an FPC.

## Supplementary Material





## Notes

Experimental details; comparison with state-of-the-art dual-modal tactile sensors; specifications and cost analysis of RoboTac skin; mathematical modeling of intrinsically self-decoupled force–temperature sensing; mechanical, optical, biocompatibility, rheological, and electrochemical characterization of the ionic conductive films; transient heat-transfer modeling and finite-element simulations; quantitative evaluation of force–temperature cross-sensitivity; electronic system and circuit design; calibration, repeatability, cyclic durability, baseline recovery, and disturbance-resistance tests; and experimental setups and additional results for object recognition, material identification, robotic manipulation, and human-robot interaction (PDF)
Principle of intrinsically self-decoupled sensing (Video S1); fabrication process of RoboTac skin (Video S2); flexibility and transparency of the ionic conductive film (Video S3); validation of intrinsically self-decoupled sensing (Video S4); repeatable response of RoboTac skin (Video S5); response of RoboTac skin under external disturbances (Video S6); response of RoboTac skin during robotic contact with different materials (Video S7); response of RoboTac skin during robotic grasping of water cups at different temperatures (Video S8); robotic preparation of the ionic conductive film precursor solution using RoboTac skin (Video S9); robotic tea-making using RoboTac skin (Video S10); simultaneous measurement of human pulse and skin temperature using RoboTac skin (Video S11); and structural and encapsulation integrity of RoboTac skin before and after prolonged cyclic testing (Video S12) (ZIP)
