# Supplementary material for: Intrinsic Force–Temperature Self-Decoupling Enables Human-like Tactile Sensing in a Soft Ionic Skin
Source: ACS Nano. 2026 Jul 25;20(31):22195–214. doi: 10.1021/acsnano.6c09063 (PMC13472318; doi:10.1021/acsnano.6c09063)
Supplement: Supplementary file 1 [file nn6c09063_si_001.pdf]

Supplementary Materials for

Intrinsic Force-Temperature Self-Decoupling Enables Human-Like Tactile Sensing in a Soft Ionic Skin

Authors:

Yu Feng[1,2]†, Jiankun Li[1,2]†, Cong Wu[1,6], Senlin Hou[1], Yehui Liu[1], Hui Sun[1,2], Meng Chen[1,2], Ziyi Li[1], Zhi Li[3], Guanglie Zhang[1,2]*, Steven Wang[1], Vellaisamy A. L. Roy[4], Xinge Yu[5], Wen Jung Li[1,2]*

Affiliations:

[1]Department of Mechanical Engineering, City University of Hong Kong, Hong Kong SAR, China
[2]CAS-CityU Joint Laboratory for Robotic Research, City University of Hong Kong, Hong Kong SAR, China
[3]College of Computer Science and Software Engineering, Shenzhen University, Shenzhen, 518000, China
[4]School of Science and Technology, Hong Kong Metropolitan University, Hong Kong SAR, China
[5]Department of Biomedical Engineering, City University of Hong Kong, Hong Kong SAR, China
[6]School of Engineering and Technology, China University of Geosciences (Beijing), Beijing, 100083, China

*Corresponding author. Email: gl.zhang@cityu.edu.hk, wenjli@cityu.edu.hk
†These authors contributed equally to this work.

Table S1. Overall comparison of state-of-the-art soft dual-modal tactile sensors and RoboTac skin.

Decoupling type	Sensor	Sensing Specificity	Transduction Type	Fabrication complexity	Structure complexity	Flexibility	Integrability difficulty	Cost	Notes
Specific sensing integration	FIN-shaped field-coupled T`FT (FCTFT) (*1*)	Force and temperature	Piezoelectric and capacitive	High	Medium	Low	Medium	High	Signal decoupling through isolation of temperature-sensing materials
	Nanoscale temperature-pressure tactile sensor (*2*)	Pressure and temperature	Resistive	Medium	Medium	High	Low	High	
	Multifunctional tactile sensor (*3*)	Pressure and temperature	Piezoelectric and thermoresistive	High	Medium	Medium	Low	Medium	
	Triboelectric nanogenerator (TENG) tactile sensor (*4*)	Pressure and temperature	TENG	Medium	High	Medium	Medium	Medium	
	Fabric-based skin (*5*)	Force and temperature	Piezoresistive and thermoresistive	High	High	High	High	Low	
	Quadruple tactile sensor (*6*)	Pressure and temperature	Piezoresistive and thermoresistive	Medium	Medium	Medium	High	Medium	
	Ion-electronic sensor (*7*)	Pressure and temperature	Piezocapacitive and thermoresistive	High	Low	Medium	Low	Medium	
	MXene/carboxyl single-walled carbon nanotube /carboxymethyl cellulose/silica nanofiber aerogel (MCCSA) sensor (*8*)	Pressure and temperature	Piezoresistive and thermoelectric	High	Low	Medium	Low	High	
	Hemisphericalshaped ion sensor (*9*)	Pressure and temperature	Piezocapacitive and thermocapacitive	High	High	Medium	Medium	High	
	Dualdielectric-layer iontronic pressure sensor (DLIPS) (*10*)	Pressure and temperature	Piezocapacitive and thermocapacitive	Medium	Low	Medium	Medium	High	
	Multisensory e-skin (11)	Pressure and temperature	Piezoresistive and thermoresistive	High	Medium	High	Low	Medium	
	Interference-free bimodal tactile sensor (*12*)	Pressure and temperature	Mechanoluminescent and thermoresistive	Medium	Low	High	Low	High	Signal decoupling by using different sensing materials
	Fingerprint sensor (*13*)	Pressure and temperature	Piezocapacitive and conductive	High	High	Medium	Medium	High	
	Finger-shaped tactile sensor (FTS) (*14*)	Force and temperature	TENG	High	High	Low	Low	Medium	
	3D force and temperature (F3T) F^{3}T (*15*)	Force and temperature	Hall effect and thermoresistive	Medium	High	Low	Medium	Medium	
	Single-electrode-mode TENG e-skin (*16*)	Pressure and temperature	TENG and thermoresistive	High	Medium	High	Medium	High	Signal decoupling by spatial distribution of sensing materials
Software decoupling	TFT-based sensor (*17*)	Pressure and temperature	Piezoresistive and carrier mobility	High	Medium	Medium	Low	High	Signal decoupling with compensation
	Conductive ink-based sensor (*18*)	Pressure and temperature	Piezoresistive and thermoresistive	Low	Low	Medium	Low	Low	Simple structure and easy integration, while insufficient signal decoupling
	CNT film-based	Pressure and	Piezoresistive	Low	Low	Medium	Low	Medium	

	sensor (*19*)	temperature	and thermoresistive						
	Parallel-connected triboelectric generator and ionogel tactile sensor (p-TITS) (*20*)	Pressure and temperature	TENG and thermal impedance	Medium	Medium	Medium	Low	Medium	
	Multifunctional flexible tactile sensor (*21*)	Pressure and temperature	Resistive	High	High	High	Low	High	Easy integration with insufficient signal decoupling
	TLC-based sensor (*22*)	Force and temperature	Visuotactile	Low	Medium	Low	Medium	High	Simple structure with inefficient decoupling compensation
Hardware decoupling	Fingerprint-shaped fluidic sensor (*23*)	Force and temperature	Resistive	Medium	Medium	High	Low	Medium	Signal decoupling with complicated design
	Intrinsically soft robotic sensor (*24*)	Force and temperature	Piezocapacitive and thermal impedance	High	High	High	Low	High	
	Optical microfiber sensor (*25*)	Pressure and temperature	Photoelectric	Medium	Low	High	Low	High	Simple structure with complex signal processing
**Intrinsically self-decoupling**	**RoboTac skin (this work)**	**Force and temperature**	**Piezocapacitive and thermoresistive**	**Low**	**Low**	**High**	**Low**	**Low**	**Easy fabrication, simple structure, intrinsic signal decoupling, high flexibility, and easy integrability**

Notes: Dual-modal sensing can be achieved through various strategies. One approach involves further isolating and protecting the temperature-sensitive material to prevent mechanical interference, a method known as specific sensing integration. Another strategy, called software decoupling, compensates for temperature effects by calibrating force response curves under different temperatures, thereby correcting the force signal. Hardware decoupling avoids crosstalk between temperature- and force-sensitive materials by spatially separating the sensing units. In contrast, intrinsically self-decoupling achieves ultra-low crosstalk dual-modal sensing through the inherent properties of a single sensing material, without the need for additional compensation, separation, or hardware modifications.

Table S2. Sensing performance comparison of state-of-the-art soft dual-modal tactile sensors and RoboTac skin.

Sensor	Force/pressure sensitivity	Force resolution	Force range	Temperature sensitivity	Temperature resolution	Temperature range
Human fingertip skin	0.001 N^{-1} (*26*)	0.025 N (applied force 0.5 N) (*27*)	0-20 N (*26-28*)	20% °C^{-1} (*29-31*)	0.1 °C (*32*)	5-60 °C (*32, 33*)
FIN-shaped field-coupled TFT (FCTFT) (*1*)	45.2 nA·kPa^{-1}	10 mN	0-1 N	~1 °C^{-1}	1 °C	-10 °C-50 °C
Nanoscale temperature-pressure tactile sensor (*2*)	0.1329 kPa^{-1}	NA	0-2 kPa	1.196% °C^{-1}	NA	22.4-65 °C
Multifunctional tactile sensor (*3*)	0.5 kPa^{-1} (0-150 kPa); 0.13 kPa^{-1} (150-300 kPa)	NA	0-300 kPa	NA	NA	25-50 °C
Triboelectric nanogenerator (TENG) tactile sensor (*4*)	9.21 kPa^{-1} (< 8.36 kPa); 1.5 kPa^{-1} (8.36-100 kPa)	NA	0-100 kPa	NA	NA	25-200 °C
Fabric-based skin (*5*)	NA	0.7 N	0-70 N	NA	0.78 °C	27-55 °C
Quadruple tactile sensor (*6*)	117 mV·kPa^{-1}	0.01 N	0-20 kPa	0.17% °C^{-1}	NA	NA
Ion-electronic sensor (*7*)	1 kPa^{-1}	1 Pa	0-300 kPa	-0.47% °C^{-1} (0-20 °C); -0.24% °C^{-1} (20-50 °C)	NA	0-50 °C
MXene/carboxyl single-walled carbon nanotube /carboxymethyl cellulose/silica nanofiber aerogel (MCCSA) sensor (*8*)	1.21 kPa^{-1}	0.025 Pa	NA	39.2 μV·K^{-1}	0.01 K	5-40 K
Hemisphericalshaped ion sensor (*9*)	0.038 kPa^{-1}	10 Pa	0-30 kPa	4.5% °C^{-1}	NA	25-55 °C
Dualdielectric-layer iontronic pressure sensor (DLIPS) (*10*)	2.46 kPa^{-1} (0.001-6 kPa); 72548.7 kPa^{-1} (6-100 kPa); 1241.6 kPa^{-1} (100-420 kPa)	0.832 Pa	0.001-420 kPa	-224.5% °C^{-1}	NA	30-50 °C
Multisensory e-skin (11)	8.71×10^{-5} kPa^{-1}	0-120 kPa	NA	0.164% °C^{-1}	NA	20-80 °C
Interference-free bimodal tactile sensor (*12*)	NA	2 N	NA	-0.6% °C^{-1}	NA	21-60 °C
Fingerprint sensor (*13*)	1.78×10^{-3} kPa^{-1} (< 350 kPa); 9.65×10^{-5} kPa^{-1} (> 350 kPa)	NA	0-1.6 MPa	0.03% °C^{-1}	NA	30-45 °C
Finger-shaped tactile sensor (FTS) (*14*)	NA	NA	0-3 N	NA	NA	NA
3D force and temperature (F^3T) (*15*)	10531 μT·N^{-1} (0-1 N N); 377 μT·N^{-1} (>1 N)	0.01 N (0-1 N); 0.03 N (>1 N)	0-7.2 N	NA	0.5 °C	20-80 °C
Single-electrode-mode TENG e-skin (*16*)	5.07 mV·Pa^{-1} (0-1720 Pa); 1.89 mV·Pa^{-1} (1720-3650 Pa)	NA	0-3650 Pa	NA	0.5 °C	25-100 °C
TFT-based sensor (*17*)	2.64 kPa^{-1} (0-1 kPa); 0.22 kPa^{-1} (1-6 kPa); 0.02 kPa^{-1} (6-20 kPa)	NA	0-20 kPa	-2.72% °C^{-1}	0.5 °C	25-50 °C
Conductive ink-based sensor (*18*)	610.208 kPa^{-1}	NA	0-250 kPa	17.2% °C^{-1}	NA	20-80 °C
CNT film-based sensor (*19*)	0.67 kPa^{-1}	2 mN	0-12 kPa	1.41% °C^{-1}	0.5 °C	-50-150 °C
Parallel-connected triboelectric generator and ionogel tactile sensor (p-TITS) (*20*)	0.03419 mV·kPa^{-1}	NA	0-90 kPa	NA	1 °C	25-45 °C

Multifunctional flexible tactile sensor (*21*)	2.71 × 10^{-4} kPa^{-1}	40 Pa	0-6.1 kPa	0.104% $°C^{-1}$	0.5 °C	25-80 °C
TLC-based sensor (*22*)	NA	0.7 N	0-16 N	NA	0.4 °C	25-31 °C
Fingerprint-shaped fluidic sensor (*23*)	0.32 N^{-1} (0-13.5 N)	NA	0-13.5 N	0.41%° C^{-1} (20-50 °C)；0.21% $°C^{-1}$ (50-80 °C)	0.5 °C	20-80 °C
Intrinsically soft robotic sensor (*24*)	NA	NA	NA	NA	0.1 °C	-40-100 °C
Optical microfiber sensor (*25*)	0.045 kPa^{-1}	NA	0-22 kPa	1.205 nm$°C^{-1}$	0.5 °C	25-95 °C
**RoboTac skin (this work)**	**0.0079 N^{-1} (2-20 N)**	**0.01 N**	**0-20 N**	**-1.02% $°C^{-1}$ (25-98 °C)**	**0.1 °C**	**2-98 °C**

Notes: The human fingertip skin, as the primary interface for tactile interaction with the external environment, exhibits outstanding capabilities in simultaneously perceiving force and temperature (*34-37*). Table S2 presents the dual-modal sensing performance of the state-of-the-art soft dual-modal tactile sensors listed in Table S1, along with that of RoboTac skin. Similar to human skin, the RoboTac skin intrinsically possesses self-decoupled dual-modal sensing of force and temperature. Furthermore, this table highlights its favorable sensing performance, establishing a solid foundation for enabling embodied tactile perception in robots.

Table S3. Specifications of RoboTac skin.

Specification	Force sensing	Temperature sensing
**Sensitivity**	53.78 %/N (0-0.4 N)<br>7.49 %/N (0.4-2 N)<br>0.79 %/N (2-20 N) *	-18.29 %/°C (2-25 °C)<br>-1.02 %/°C (25-98 °C)
**Linearity**	0.93 (0-0.4 N)<br>0.97 (0.4-2 N)<br>0.97 (2-20 N)	0.99 (2-25 °C)<br>0.91 (25-98 °C)
**Resolution**	0.01 N	0.1 °C
**Response time**	0.25 s	0.01 s **
**Range**	0-20 N	2-98 °C
**Size**	20 x 20 x 0.25 mm^3	
**Weight**	0.15 g	
**Areal mass density**	0.038 g/cm^2	
**Cost**	3.24 RMB	

* The force response curve of RoboTac skin follows a logarithmic trend, but it can be well approximated by piecewise linear fitting over three segments, resulting in good linearity.

** The temperature response time of RoboTac skin is calibrated by comparing the time at which the thermocouple detects a change in the adjustable constant temperature platform with the corresponding moment when RoboTac skin registers a temperature response. This rapid response time is further supported by simulation results, as shown in Fig. S10.

Table S4. Cost of RoboTac skin.

Materials	Cost (RMB)
Sensing layer	0.038
Insulation layer	0.2
Top and bottom FPCB	3
**Total cost**	**3.24**

Notes: All material costs here are calculated based on the actual area used in RoboTac skin (3.24 RMB=~0.45 USD). Each fabricated ionic conductive film (0.38 RMB) can be cut into approximately 10 pieces of the sensing layers, resulting in a material cost of about 0.038 RMB per layer (BOM cost per cm^2=0.81 RMB/cm^2). Additionally, the cost of small-batch fabricated FPCBs is approximately 3 RMB per piece (data source from JLC Technology Group).

Table S5. Cost of raw chemicals of the ionic conductive film.

Raw chemicals	Unit price (g/RMB)	Mass (g)	Cost (RMB)
Pectin	1.48	0.2	0.3
Chitosan	0.87	0.01	0.0087
PEG400	0.1	0.1	0.01
Glycerol	0.42	0.1	0.042
$CaCl_2$	0.09	0.2	0.018
Citric acid	0.4	0.02	0.008
**Total cost (RMB)**	**0.38**		

Data source: Shanghai Aladdin Biochemical Technology Co., Ltd. The cost of raw chemical reagents can be further reduced with continued mass production.

Table S6. Main thermal parameters of six materials.

Materials	Specific Heat c_p (J/kg·K)	Thermal Conductivity k (W/m·K)	Density ρ (kg/m³)
**Copper**	380-400	390-400	8900-9000
**Aluminum**	880-960	220-250	2600-2800
**Iron**	440-470	70-100	7700-8000
**Wood**	1500-2500	0.12-0.15	400-700
**PLA (60% filler)**	1800	0.2	600
**TPU (60% filler)**	2000	0.25	480

Notes: This experiment demonstrates the use of a RoboTac skin-equipped robotic gripper to identify object materials through the thermal exchange process. The objects used have identical dimensions, each being a cube with 25 mm edge length, ensuring consistent volume and surface area. Although the actual basic thermal parameters of these materials fall within certain ranges, the midpoint values of these ranges were selected for subsequent simulations to facilitate modeling.

Text S1. Dual-modal sensing mathematical model

The intrinsically self-decoupled dual-modal sensing capability of RoboTac skin originates from its ionic conductive film, which serves as the sensing material, and the multi-electrode configuration embedded on the top and bottom FPCBs within a sandwich-like structure. The capacitance response is predominantly governed by normal force, whereas the resistance response is predominantly governed by temperature. Small cross-channel contributions may remain under practical conditions, as quantitatively evaluated in Text S4. In this section, we study theoretical models to describe their sensing mechanisms, including a piezocapacitive model for force detection and a thermoresistive model for temperature sensing.

Piezocapacitive sensing model for force

The top and bottom electrodes sandwich the ionic conductive film, forming a classic sandwich structure that functions as a parallel-plate capacitor. The capacitance of this structure can be described as follows:

$$C = \frac{\varepsilon_0 \varepsilon_r A}{d} \tag{1}$$

Here, ε_0 , ε_r , d, and A represent the vacuum permittivity, relative permittivity, distance between the top and bottom FPCBs, and electrode area, respectively. When RoboTac skin is subjected to a normal force, the thickness of the sensing layer changes from d_0 to d, leading to the following expression:

$$\Delta d = d - d_0 \tag{2}$$

The sensing layer is treated as a linear elastic material. According to Hooke's law, we have:

$$\frac{-\Delta d}{d_0} = -\frac{\sigma}{E} = -\frac{F}{AE} \tag{3}$$

Here, E and σ denote the Young's modulus of the ionic conductive film and the applied stress, respectively. By rearranging the equation, we obtain:

$$\Delta d = -\frac{Fd_0}{AE} \tag{4}$$

By combining equations (2) and (4), the thickness of the sensing layer under applied force can be expressed as:

$$d = d_0\left(1 - \frac{F}{AE}\right) \tag{5}$$

Substituting equation (5) into equation (1) yields:

$$C = \frac{\varepsilon_0 \varepsilon_r A}{d_0}\left(1 - \frac{F}{AE}\right)^{-1} \tag{6}$$

The initial capacitance is given as C_0 , so the relative change in capacitance can be expressed as:

$$\frac{\Delta C}{C_0} = \frac{C - C_0}{C_0} = \left(1 - \frac{F}{AE}\right)^{-1} - 1 \tag{7}$$

From the above equation, it can be seen that since the electrode area A can be considered constant, the relative change in capacitance depends primarily on the applied force F and is directly proportional to it. When $F \ll AE$, a Taylor expansion of equation (7) gives the following relationship:

$$\begin{aligned}\frac{\Delta C}{C_0} &= -1+1+\frac{F}{AE}+\left(\frac{F}{AE}\right)^2+\cdots \\ &\simeq \frac{F}{AE}\end{aligned} \tag{8}$$

From the above equation, we observe that as the pressure increases, the response of the capacitance change rate exhibits a nonlinear increase, following a logarithmic or power-law trend.

When the temperature T changes, the capacitance can be described as follows:

$$\begin{aligned}&C=\varepsilon_r(T)\frac{\varepsilon_0 A}{d} \\ &\varepsilon_r(T)=\varepsilon_{r0}(1+\alpha_T \Delta T)\end{aligned} \tag{9}$$

Here, α_T represents the temperature sensitivity coefficient (*38*, *39*). From equation (6), we have:

$$C=\frac{\varepsilon_0\varepsilon_{r0}(1+\alpha_T\Delta T)A}{d_0}\left(1-\frac{F}{AE}\right)^{-1} \tag{10}$$

At this point, the relative change in capacitance is still given by:

$$\begin{aligned}\frac{\Delta C}{C_0} &= \frac{C-C_0}{C_0} \\ &= \frac{\frac{\varepsilon_0\varepsilon_{r0}(1+\alpha_T\Delta T)A}{d_0}\left(1-\frac{F}{AE}\right)^{-1}-\frac{\varepsilon_0\varepsilon_{r0}(1+\alpha_T\Delta T)A}{d_0}}{\frac{\varepsilon_0\varepsilon_{r0}(1+\alpha_T\Delta T)A}{d_0}} \\ &= \left(1-\frac{F}{AE}\right)^{-1}-1\end{aligned} \tag{11}$$

Thus, we have established the mechanism for measuring force using the capacitance change rate of RoboTac skin. We find that the capacitance change rate is primarily dependent on the force. Although temperature can cause changes in the relative permittivity of the ionic conductive film, which in turn affects the measurement

capacitance of RoboTac skin, the effect of temperature remains small within the calibrated range by solving for the capacitance change rate. This demonstrates the normalized capacitive response is dominated by force, while the experimentally observed temperature-dependent contribution remains small within the calibrated range. Then, we will model the mechanism of temperature measurement through the thermoresistive effect in RoboTac skin.

Thermoresistive sensing model for temperature

The ionic conductive film is an ionic conductive gel. For this type of ionic conductive material, the resistance is primarily influenced by temperature changes through the ionic mobility μ, which typically follows an Arrhenius relationship (*25*-*27*), as shown below:

$$\mu(T) = \mu_0 \cdot \exp\left(-\frac{E_a}{k_B T}\right) \tag{12}$$

Here, μ_0 represents the ion mobility at high temperature limits, E_a is the ionic migration activation energy, k_B is the Boltzmann constant, and T is the absolute temperature. Since higher ionic mobility leads to greater conductivity, i.e., the conductivity σ is proportional to μ_0, we have:

$$\sigma(T) = nq\mu(T) \sim \exp\left(-\frac{E_a}{k_B T}\right) \tag{13}$$

The relationship between resistance R and conductivity σ is given by:

$$R = \frac{L}{\sigma A} \tag{14}$$

Here, L and A represent the distance between the bottom electrodes and the effective cross-sectional area for electrical conduction. By combining equations (13) and (14), the relationship between the temperature-dependent resistance and the temperature-

dependent conductivity is given by:

$$R(T) = \frac{L}{\sigma(T)A} \sim \exp\left(\frac{E_a}{k_B T}\right) \tag{15}$$

From the above equation, we observe that the resistance decreases nonlinearly with increasing temperature, meaning that as the temperature increases, the resistance decreases, exhibiting a negative temperature coefficient. By substituting equation (12) into equation (15) and introducing the initial resistance R_0 and temperature T_0, the thermoresistive model is given as follows:

$$R(T) = R_0 \cdot \exp\left(\frac{E_a}{k_B} \cdot \left(\frac{1}{T} - \frac{1}{T_0}\right)\right) \tag{16}$$

The above equation indicates that the resistance of the ionic conductive film decays exponentially with temperature changes, which aligns with the actual behavior of ionically conductive materials in thermoresistive effects (*43*, *44*), exhibiting a negative temperature coefficient. Although *R(T)* is theoretically an exponential function, within the working temperature range of 0-100 °C (273-373 K), this exponential function can be approximated as a linear function through a Taylor expansion, as shown below:

$$\frac{1}{T} \approx \frac{1}{T_0} - \frac{T - T_0}{T_0^2} \tag{17}$$

Substituting the above equation into equation (16) yields:

$$R(T) \approx R_0 \cdot \exp\left(\frac{E_a}{k_B T_0}\left(1 - \frac{T - T_0}{T_0}\right)\right) \tag{18}$$

After further performing a Taylor expansion on the above equation, we obtain:

$$R(T) = R_0 \cdot \left[1 - \frac{E_a}{k_B T_0^2} \cdot (T - T_0) \right] \tag{19}$$

Let the temperature coefficient α_T be related to the change in resistance ΔR as follows:

$$\begin{aligned} \alpha_T &= - \frac{E_a}{k_B T_0^2} < 0 \\ \Delta R &= R(T) - R_0 \end{aligned} \tag{20}$$

Substituting the above equation into (19), the relative change in the resistance of the ionic conductive film is given by the following relationship:

$$\frac{\Delta R}{R_0} = \alpha_T (T - T_0) \tag{21}$$

From the above equation, we observe that the relative change in resistance is inversely proportional to temperature, meaning the ionic conductive film exhibits a negative temperature coefficient. When an external force F is applied, the shape of the ionic conductive film undergoes slight changes, which results in small variations in the distance between the bottom electrodes and the effective cross-sectional area for electrical conduction. This causes equation (14) to change to the following:

$$R(T,F) = \frac{L(F)}{A(F)} \frac{1}{\sigma(T)} \tag{22}$$

Under small changes, the ionic conductive film can be treated as a linear elastic material. The variation in the distance between the bottom electrodes and the effective cross-sectional area for electrical conduction is given by:

$$\begin{aligned} L(F) &= L_0 \left(1 - \frac{F}{k L_0} \right) \\ A(F) &= A_0 \left(1 + \eta \frac{F}{k L_0} \right) \end{aligned} \tag{23}$$

Here, k and η represent the elastic stiffness and the viscous damping coefficient, respectively. Substituting the above equation into (11), we obtain:

$$R(T,F) = \frac{L_0}{A_0}\left[1 - (1+\eta)\frac{F}{kL_0}\right]\frac{1}{\sigma(T)} \tag{24}$$

Let λ be the force sensitivity coefficient. After rearranging, we obtain:

$$\begin{gathered} R(T,F) = R_0(T)(1-\lambda F) \\ \lambda = \frac{1+\eta}{kL_0} \end{gathered} \tag{25}$$

After introducing the force F, the corrected formula for the relative change in resistance becomes:

$$\frac{\Delta R}{R_0} = \alpha_T (T - T_0) - \lambda F \tag{26}$$

Since the elastic stiffness of the ionic conductive film is relatively high and the viscous damping coefficient is small, the force sensitivity coefficient is low. Therefore, the influence of the force on the relative change in resistance is minimal and can be neglected. As a result, the formula for the relative change in resistance remains as shown in equation (21). Based on the thermoresistive properties of the ionic conductive film, temperature measurement can be achieved while suppressing cross-channel interference from force, thus decoupling force from temperature measurement. Furthermore, by combining the previously discussed decoupling model for force measurement, we have established the dual-modal self-decoupling mathematical model for RoboTac skin. This self-decoupling characteristic does not arise from the selection of two different sensing materials, software compensation algorithms, or hardware-based decoupling. Rather, the decoupling feature of RoboTac skin originates from the device itself, meaning it is an intrinsic self-decoupling property.

Text S2. Characterization of the ionic conductive films.

Tensile tests of the ionic conductive films

The tensile tests were conducted on a tensimeter (LT-5000, Reotai Precision Instrument Co., Ltd.) equipped with a 10 N load cell at a crosshead speed of 20 mm/min. The tested rectangular ionic conductive film samples (30 mm in length, 20 mm in width, and 0.5 mm in thickness) were prepared using a carbon dioxide (CO_2) laser engraver (P2, Makeblock Co., Ltd.).

UV-Vis tests of the ionic conductive films

The tested ionic conductive film samples were cut into small pieces (1 cm x 1 cm), respectively, and tested by a UV-Vis spectrophotometer (SpectraMax M5e Multi-Mode Microplate Reader, Molecular Devices Company).

Cell viability tests of the ionic conductive films

Madin-Darby canine kidney (MDCK) cells are maintained in the standard CO_2 incubator (HERACELL 150i, Thermo Scientific Co., Ltd.) with an environment of 5% CO_2 and 37 °C; while cells are cultured in the growth medium (GM) of Minimum Essential Medium Eagle (Procell Co., Ltd.) as supplemented with 10% Fetal Bovine Serum and 1% Penicillin-Streptomycin-Neomycin (Gibco Co., Ltd.). The tested ionic conductive film samples were autoclaved at 121 °C for 30 minutes. After sterilization, each of the samples (0.2 g) was immersed in GM at 37 °C for 24 hours; after removing those samples, the remaining solutions were used to test for biocompatibility. MDCK cells (P12) were first seeded in petri dishes and cultured in GM for 24 hours. Then, GM was substituted with different leaching solutions to culture cells for another 24 hours, respectively. In addition, the cells continued to grow in GM as a control. LIVE/DEAD Cell Imaging kit containing Calcein AM and

Propidium Iodide (Life Technologies Co., Ltd.) was used to calculate the cell viability.

Electrochemical tests of the ionic conductive films

The electrochemical characteristics tests (impedance and linear sweep voltammetry) were performed using an electrochemical workstation (DH7000D, Jiangsu Donghua Analytical Instrument Co., Ltd.). The tested rectangular ionic conductive film samples (30 mm in length, 10 mm in width, and 0.5 mm in thickness) were prepared using a carbon dioxide (CO_2) laser engraver (P2, Makeblock Co., Ltd.).

Text S3. Model of transient heat conduction for the object and the gripper.

Transient heat-conduction model of the object-gripper system

When the robotic gripper grasps an object at a certain temperature and forms close contact, heat transfer occurs through the contact surface between the two bodies until thermal equilibrium is reached. This process is characterized as transient heat conduction. RoboTac skin is positioned between the object and the gripper, and due to its negligible thickness, it can be approximated as part of the contact interface. In this setup, we focus on the temperature evolution at the center point of the contact surface. Since the object-gripper interface is assumed to be perfectly bonded, thermal contact resistance is negligible. Meanwhile, the non-contact surfaces exchange heat with the surrounding air via natural convection, which is described by Newton’s law of cooling. Therefore, the transient heat conduction in this system can be approximated as one-dimensional and follows the governing equation:

$$\rho c \frac{\partial T}{\partial t} = k \frac{\partial^2 T}{\partial z^2} \tag{27}$$

Here, $T(z, t)$ represents the temperature distribution, and ρ, c, and k denote the density, specific heat capacity, and thermal conductivity, respectively. Objects A and B (the gripper) are tightly stacked along the Z-axis, with thicknesses L_A and L_B, respectively. The initial temperature of object A is 60 °C, while that of object B is the ambient room temperature (25 °C). The initial and boundary conditions are given as follows:

$$
\begin{aligned}
&T_A(z,0)=60\ °C,\ z\in[-L_A,0]\\
&T_B(z,0)=25\ °C,\ z\in[0,L_B]\\
&k_A\frac{\partial T}{\partial z}\bigg|_{z=-L_A}=h_A(T(-L_A,t)-T_\infty)\\
&k_B\frac{\partial T}{\partial z}\bigg|_{z=L_B}=h_B(T(-L_B,t)-T_\infty)\\
&T_A(0,t)=T_B(0,t)=T_{int}(t)\\
&k_A\frac{\partial T_A}{\partial z}\bigg|_{z=0}=k_B\frac{\partial T_B}{\partial z}\bigg|_{z=0}
\end{aligned}
\tag{28}
$$

Due to the small sizes of the object and the gripper, as well as the strong thermal diffusivity of the materials, the lumped capacitance model can be used to approximate the temperature variation process. Accordingly, the thermal capacitances of A and B are given by:

$$
\begin{aligned}
C_A&=\rho_A c_A V_A\\
C_B&=\rho_B c_B V_B
\end{aligned}
\tag{29}
$$

The contact thermal conductance is denoted as h_c, and the contact area is A. The heat transfer between the two components is therefore given by:

$$
Q=h_c A(T_A-T_B) \tag{30}
$$

Where T_A and T_B represent the temperatures of object A and gripper B, respectively. According to the principle of energy conservation, the following equations hold:

$$
\begin{aligned}
C_A\frac{dT_A}{dt}&=-h_c A(T_A-T_B)\\
C_B\frac{dT_B}{dt}&=h_c A(T_A-T_B)
\end{aligned}
\tag{31}
$$

By combining the above equations, we can obtain:

$$\frac{d}{dt}(T_A - T_B) = -\left(\frac{1}{\tau}\right)(T_A - T_B)$$
$$\tau = \frac{C_A C_B}{h_c A(C_A + C_B)} \tag{32}$$

Here, τ denotes the time constant. This expression indicates that the temperature at the contact point follows an exponential decay process and represents a weighted average of the temperatures of object A and gripper B. Based on empirical formulations of heat transfer between two contacting bodies, the temperature variation at the contact interface can be approximated by a single-exponential decay function:

$$T(t) = T_\infty + (T_0 - T_\infty) \cdot e^{-t/\tau} \tag{33}$$

The empirical formulation indicates that during the heat transfer process between two contacting bodies, the temperature at the contact interface is determined by the initial and final temperatures of the two objects. The cooling curve follows an exponential decay trend.

<u>Influence of contact conditions on the system-level thermal response</u>

The temperature response measured during robotic grasping includes both the intrinsic electrical response of RoboTac skin and the transient heat-transfer process across the object-sensor interface. The interfacial heat-transfer rate is expressed as

$$\dot{Q} = h_c A_c (T_o - T_s),$$

where (h_c) is the thermal contact conductance, (A_c) is the effective contact area, and (T_o) and (T_s) are the temperatures of the object and sensor, respectively. Based on the lumped thermal model, the characteristic thermal response time can be approximated as

$$\tau_{\mathrm{th}} = \frac{C_{\mathrm{eq}}}{h_c A_c},$$

where (C_{eq}) is the equivalent thermal capacitance of the object-sensor system. Therefore, increasing the effective contact area decreases the thermal time constant. Increasing contact pressure generally improves interfacial conformity and reduces the thermal contact resistance by increasing the real contact area, thereby accelerating heat transfer. Robot motion speed does not directly enter the heat-transfer equation. Instead, it determines the available contact duration (t_c). The fraction of the temperature difference transferred during contact can be approximately described as

$$\frac{\Delta T_s(t_c)}{\Delta T_{s,\infty}} = 1 - \exp\left(-\frac{t_c}{\tau_{\mathrm{th}}}\right).$$

Thus, a longer contact duration allows the sensor temperature to approach its steady-state value more closely, whereas a short contact duration may result in an incomplete temperature response. In the present study, these relationships are used to explain the physical influence of contact conditions. Systematic experimental determination of the individual effects of contact area, pressure, and motion speed remains a subject for future investigation.

Text S4. Quantitative evaluation of cross-sensitivity.

The self-decoupled sensing behavior of RoboTac skin arises from the selective responses of its two electrical output channels to mechanical and thermal stimuli. Normal force mainly changes the capacitance by compressing the ionic conductive film in the thickness direction, whereas temperature mainly changes the resistance by affecting lateral ion transport. In practice, small cross-channel responses may still occur because of temperature-dependent dielectric properties, thermal expansion, deformation of the ionic conduction pathway, and measurement fluctuations. The cross-sensitivity was therefore quantitatively evaluated rather than assumed to be zero.

Dual-modal response model

Within the calibrated operating ranges, the normalized capacitance and resistance responses can be described using a first-order model:

$$\begin{aligned} \frac{\Delta C}{C_0} &= S_{CF}F + S_{CT}\Delta T + \varepsilon_C \\ \frac{\Delta R}{R_0} &= S_{RF}F + S_{RT}\Delta T + \varepsilon_R \end{aligned} \tag{34}$$

where C_0 and R_0 are the initial capacitance and resistance, respectively; F is the applied normal force; $\Delta T = T - T_0$ is the temperature change relative to the reference temperature T_0; S_{CF} is the force-to-capacitance sensitivity; S_{RT} is the temperature-to-resistance sensitivity; S_{CT} is the temperature-to-capacitance cross-sensitivity; S_{RF} is the force-to-resistance cross-sensitivity; and ε_C and ε_R represent measurement noise and other residual contributions. The dual-modal response can also be expressed in matrix form:

$$\begin{bmatrix} \Delta C/C_0 \\ \Delta R/R_0 \end{bmatrix} = \begin{bmatrix} S_{CF} & S_{CT} \\ S_{RF} & S_{RT} \end{bmatrix} \begin{bmatrix} F \\ \Delta T \end{bmatrix} + \begin{bmatrix} \varepsilon_C \\ \varepsilon_R \end{bmatrix} \tag{35}$$

The diagonal terms describe the principal responses of the two sensing channels,

whereas the off-diagonal terms describe the cross-channel responses. Because these sensitivity coefficients have different physical units, direct comparison between $\% \, \mathrm{N}^{-1}$ and $\% \, {}^{\circ}\mathrm{C}^{-1}$ is not meaningful. Dimensionless full-scale normalized crosstalk ratios were therefore used for comparison.

Temperature-to-force crosstalk

The influence of temperature on the capacitance channel was evaluated using the temperature calibration data collected from 2 to 98 °C without an externally applied normal force. The maximum temperature-induced capacitance drift was defined as

$$E_{T\rightarrow C} = \max_{T} \left| \frac{C(T) - C(T_0)}{C(T_0)} \right| \tag{36}$$

The full-scale force-induced capacitance response was defined as

$$A_{F\rightarrow C} = \max_{F} \left(\frac{\Delta C}{C_0} \right) - \min_{F} \left(\frac{\Delta C}{C_0} \right) \tag{37}$$

where the applied force ranged from 0 to 20 N. The normalized temperature-to-force crosstalk ratio was calculated as

$$\eta_{T\rightarrow F} = \frac{E_{T\rightarrow C}}{A_{F\rightarrow C}} \times 100\% \tag{38}$$

The maximum temperature-induced capacitance drift was 0.96%, occurring at 86 °C. The full-scale force-induced capacitance response over 0-20 N was 45.23%. Therefore,

$$\eta_{T\rightarrow F} = \frac{0.95697}{45.2334} \times 100\% = 2.11563\% \tag{39}$$

Linear regression of the capacitance response against temperature gave

$$S_{CT} = -0.003069\ \%\ ^\circ\mathrm{C}^{-1} \tag{40}$$

with $R^2 = 0.0962$, $p = 0.1083$, and a 95% confidence interval of [−0.006863, 0.000724] % ℃$^{-1}$. The low R^2 value and the statistically non-significant slope indicate that the capacitance channel did not exhibit a clear systematic dependence on temperature. The maximum temperature-induced capacitance variation was only 2.116% of the full-scale force-induced capacitance response.

Force-to-temperature crosstalk

The influence of force on the resistance channel was evaluated using force calibration data collected at twelve fixed temperatures between 25 and 80 °C. At each temperature, normal forces from 0 to 20 N were applied while the resistance response was recorded. At a given temperature, the force-induced resistance drift was defined as

$$E_{F\to R}(T) = \max_{F} \left| \frac{R(F,T) - R(0,T)}{R(0,T)} \right| \tag{41}$$

The maximum force-induced resistance drift over all force and temperature conditions was then defined as

$$E_{F\to R}^{\max} = \max_{T} \left[E_{F\to R}(T) \right] \tag{42}$$

The full-scale temperature-induced resistance response was calculated as

$$A_{T\to R} = \max_{T} \left(\frac{\Delta R}{R_0} \right) - \min_{T} \left(\frac{\Delta R}{R_0} \right) \tag{43}$$

The normalized force-to-temperature crosstalk ratio was defined as

$$\eta_{F\to T} = \frac{E_{F\to R}^{\max}}{A_{T\to R}} \times 100\% \tag{44}$$

The maximum force-induced resistance variation among all individual measurements was 5.83389%, occurring at 30 °C and 2 N. The full-scale temperature-induced resistance response was 507.293%. Thus,

$$\eta_{F\to T} = \frac{5.83389}{507.293} \times 100\% = 1.15\% \tag{45}$$

After averaging repeated measurements at each force-temperature condition, the maximum mean force-induced resistance variation was 3.59504%, occurring at 60 °C and 10 N. The mean value of $E_{F\to R}(T)$ across all investigated temperatures was 2.38989%. The force-to-resistance cross-sensitivity was also obtained by fitting the resistance response as a function of force at each temperature. The maximum absolute cross-sensitivity was

$$\max|S_{RF}| = 0.06661\ \%\ \mathrm{N}^{-1} \tag{46}$$

and the mean absolute cross-sensitivity over all temperatures was

$$mean|S_{RF}| = 0.02691\ \%\ \mathrm{N}^{-1} \tag{47}$$

These values are small compared with the resistance variation caused by temperature, showing that normal loading introduces only limited interference into the temperature-sensing channel over the investigated force range.

Temperature dependence of the force-capacitance calibration

The stability of the complete force-capacitance calibration curve was further examined at twelve temperatures ranging from 25 to 80 °C. For each force point $\boldsymbol{F_i}$, the mean capacitance response across all temperatures was calculated as

$$\bar{C}(F_i) = \frac{1}{N_T}\sum_{j=1}^{N_T}\left(\frac{\Delta C}{C_0}\right)_{F_i,T_j} \tag{48}$$

where $N_T = 12$. The corresponding standard deviation was calculated as

$$\sigma_C(F_i) = \sqrt{\frac{1}{N_T - 1}\sum_{j=1}^{N_T}\left[\left(\frac{\Delta C}{C_0}\right)_{F_i,T_j} - \bar{C}(F_i)\right]^2} \tag{49}$$

Across all nonzero force points, the mean standard deviation of the capacitance response was 1.01853%. The maximum standard deviation was 1.4803%, occurring at 8 N. The small variation indicates that the force-capacitance calibration remained stable over the investigated temperature range. The force response was further divided into three calibrated regions: 0-0.4 N, 0.4-2 N, and 2-20 N. At each temperature, the force sensitivity was obtained from the slope of the linear fit within the corresponding force interval. The average sensitivities were

$$\begin{aligned} S_{CF}^{0-0.4\ \mathrm{N}} &= 34.346 \pm 2.729\ \%\ \mathrm{N}^{-1} \\ S_{CF}^{0.4-2\ \mathrm{N}} &= 7.547 \pm 0.699\ \%\ \mathrm{N}^{-1} \\ S_{CF}^{2-20\ \mathrm{N}} &= 0.9379 \pm 0.0567\ \%\ \mathrm{N}^{-1} \end{aligned} \tag{50}$$

To compare the temperature dependence of the three force regions, the sensitivity obtained at each temperature was normalized by the mean sensitivity of the corresponding region:

$$S_{\mathrm{norm}}(T) = \frac{S_{CF}(T)}{\bar{S}_{CF}} \tag{51}$$

As shown in Fig. S13C, the normalized sensitivities remained close to unity over 25-80 °C. Although moderate variations in the fitted local slopes were observed, the overall force calibration characteristics were maintained throughout the investigated temperature range.

Overall cross-sensitivity assessment

The cross-sensitivity results can be summarized using the following full-scale normalized response matrix:

$$\mathbf{M}_{\mathrm{norm}} = \begin{bmatrix} 100\% & 2.11563\% \\ 1.15\% & 100\% \end{bmatrix} \tag{52}$$

The rows correspond to the capacitance and resistance outputs, while the columns correspond to force and temperature inputs. The diagonal terms represent the principal responses, and the off-diagonal terms represent the normalized crosstalk ratios. After numerical evaluation, the temperature-to-force and force-to-temperature crosstalk ratios were 2.116% and 1.15%, respectively. Although the cross-channel responses were not strictly zero, they were much smaller than the corresponding principal responses within the calibrated operating ranges. These results support the low-interference force and temperature sensing capability of RoboTac skin without the need for algorithmic compensation or spatial separation of different sensing materials.

Text S5. Simulation of experiments based on RoboTac skin.

Steady-state heat transfer simulation of RoboTac skin

The mechanical model of RoboTac skin was constructed using SOLIDWORKS. It consists of a 20 × 20 × 0.25 mm thin film composed of four layers arranged from top to bottom: top PI layer, PI tape, ionic conductive film, and bottom PI layer, with respective thicknesses of 0.07 mm, 0.07 mm, 0.05 mm, and 0.07 mm. A thermal model of the steady-state heat transfer process between RoboTac skin and an object was then built using COMSOL. The ionic conductive film was defined as a gel material. The temperature of the object was varied from 30 °C to 100 °C in steps of 10 °C, and the corresponding steady-state temperature curve of the sensor was obtained (Fig. S10). The results show that when RoboTac skin comes into contact with objects at different temperatures, its temperature rapidly reaches that of the object, indicating a fast thermal response time.

Simulation results of the steady-state temperature of objects heated by a heat source

A mechanical model of the object and the heating stage was constructed using SOLIDWORKS. The object was modeled as a cube with a side length of 25 mm, and the heating stage was modeled as a rectangular block with a base of 100 mm and a thickness of 10 mm, consistent with the actual setup. The bottom surfaces of the two components were in tight contact. A thermal model of the steady-state heat transfer process between the object and the heating stage was then established using COMSOL. The material of the heating stage was set as thermally conductive copper, consistent with the experimental condition. The object was assigned different materials in separate simulations, including copper, aluminum, iron, and wood. All non-contact surfaces in the system were set to convective heat flux, with a convective heat transfer coefficient of 10 $W/(m^2 \cdot K)$ to simulate air cooling. The heating stage was maintained at 63 °C as the heat source, while the object started at room

temperature (25 °C). A point probe was placed at the center of the side surface of the object to monitor the temperature, and a steady-state heat transfer analysis was performed.

The resulting temperature curves at the side surface center are shown in Fig. S20. The steady-state temperatures for copper, aluminum, iron, TPU, PLA, and wood were found to be 62.93 °C, 62.98 °C, 62.71 °C, 39.70 °C, 37.90 °C, and 35.88 °C, respectively. The steady-state temperatures of copper, aluminum, and iron are close to that of the heating stage, due to their relatively similar specific heat capacities. In contrast, the TPU, PLA, and wood reached a significantly lower temperature, as they have a higher specific heat capacity, requiring more energy to increase in temperature. However, because of heat loss through air convection, the TPU, PLA, and wood could not continue heating indefinitely, resulting in a large temperature difference from the heating stage. This simulation result demonstrates that, when volume and surface area are held constant, the specific heat capacity of a material has a direct impact on the steady-state temperature it can reach when absorbing heat from a source in an air environment.

Simulation results of the transient heat conduction of different objects

First, a mechanical model of the object and the gripper was constructed using SOLIDWORKS. The object was modeled as a cube with a side length of 25 mm, consistent with the actual dimensions. The gripper was modeled as a rectangular block with a base of 25 mm and a thickness of 10 mm, representing the effective volume involved in actual heat transfer. The bottom surfaces of the two components were in tight contact.

Subsequently, a thermal model of the transient heat transfer process between the object and the gripper was built using COMSOL. The gripper material was set as ABS, which is consistent with the actual setup. The object material was assigned to different simulations, such as copper, aluminum, iron, TPU, PLA, or wood. All non-contact

surfaces were set to convective heat flux, with a convective heat transfer coefficient of 10 W/(m²·K) to simulate air cooling. The initial temperature of the object was set to 60 °C, while the gripper was set at room temperature (25 °C). A point probe was placed at the center of the contact surface between the object and the gripper, and a transient heat transfer analysis was conducted to obtain the cooling curve at this point (Fig. S21). An exponential function was used to fit the cooling curves, and the fitted cooling curves are as follows:

$$
\begin{aligned}
&T_{\text{copper}} = 31.6\exp\left(-\frac{1}{1594.8}t\right) + 25.3 \\
&T_{\text{aluminium}} = 31.1\exp\left(-\frac{1}{1273.1}t\right) + 25.3 \\
&T_{\text{Iron}} = 32.3\exp\left(-\frac{1}{1669.4}t\right) + 24.9 \\
&T_{\text{wood}} = 31.3\exp\left(-\frac{1}{801.2}t\right) + 25.2 \\
&T_{\text{PLA}} = 30.6\exp\left(-\frac{1}{710.2}t\right) + 25.2 \\
&T_{\text{TPU}} = 29.7\exp\left(-\frac{1}{575}t\right) + 25.2
\end{aligned}
\tag{53}
$$

Therefore, the time constants of the cooling curves for the six materials are obtained as follows:

$$
\begin{aligned}
&\tau_{\text{copper}} = 1594.8\ s \\
&\tau_{\text{aluminium}} = 1273.1\ s \\
&\tau_{\text{Iron}} = 1669.4\ s \\
&\tau_{\text{wood}} = 801.2\ s \\
&\tau_{\text{PLA}} = 710.2\ s \\
&\tau_{\text{TPU}} = 575\ s
\end{aligned}
\tag{54}
$$

From this, we observe that at the same temperature, the cooling rates of TPU, PLA, wood, aluminum, copper, and iron decrease in sequence. This result indicates that the cooling rate of an object is not only related to its thermal conductivity but also to its

heat capacity. Together, these two factors determine the object's cooling rate. By analyzing the actual room-temperature cooling curve measured by RoboTac skin, the equivalent heat capacity and equivalent heat transfer coefficient can be calculated, which can then be used for material identification.

Text S6. Setup of the demonstration.

For all demonstrations in this work, RoboTac skin was fixed to the inner surface of the robotic gripper (PiPER, AgileX Robotics) using double-sided adhesive tape (VHB tape, 3M Company), while the compact signal processing board was mounted on the outer surface. RoboTac skin and the compact signal processing board were connected via an FPC.

Demonstration setup of RoboTac skin on object identification

In the robot grasping task, the objects (glue stick, test tube, disinfectant, tape, yarn ball, lubricant, phial, keycap, sausage, sugar cube, chip, and litchi) were sourced from local grocery stores. The tactile data acquisition software was launched on the host computer to initiate recording, after which the robot was teleoperated to the target grasping position. The robotic gripper (opening width 100 mm) was then closed to secure the object. After maintaining the grasp for a predetermined duration, the gripper was opened, and the acquisition software was closed to end recording. For each object type, 50 trials were conducted; 40 datasets were used for training and 10 for validation, the latter being used to generate the confusion matrix.

Demonstration setup of RoboTac skin on material identification

In the experiments, all six objects had identical dimensions and nominal contact areas and were tested using the same heating platform, robotic gripper, sensor, and measurement procedure under the same ambient conditions. Nevertheless, the fitted time constant may still be influenced by the actual interfacial contact area, clamping force, surface roughness, initial temperature, and thermal contact resistance. Therefore, the experimentally obtained time constant is interpreted as an effective time constant of the object-sensor-gripper system under the specified testing conditions, rather than an intrinsic material constant.

Demonstration setup of RoboTac skin for special tasks

The aforementioned robotic system was also employed to perform specialized tasks. The object to be grasped was a beaker containing 120 g of water (with a beaker weight of 80 g). Three beakers with water at different temperatures (25 °C, 15 °C, and 50 °C) were used. Through teleoperation, the robot was navigated to the location of the beaker, where it grasped the beaker using the gripper, then moved to a different platform to place the beaker down. A motion camera (GoPro 12, GoPro, Inc.) and an infrared camera (K20, Hangzhou Hikvision Digital Technology Co., Ltd.) were used to record the entire experimental process. Meanwhile, RoboTac skin continuously measured force and temperature signals throughout the task. The integration of RoboTac skin endowed the robot with the capability to perceive different liquid temperatures, laying the foundation for its future applications as a tactile sensor in service robotics.

Another special task was a chemistry experiment involving the preparation of a precursor solution for an ionic conductive film. Initially, raw materials were added to deionized water according to the required formulation. At this stage, the mixture was a suspension, as the solutes were not fully dissolved and had not yet formed an ionic conductive solution. Through teleoperation, the robotic arm was directed to the beaker containing the suspension and grasped it with the gripper. The beaker was then moved above an alcohol lamp for heating. When RoboTac skin detected that the temperature had reached approximately 80 °C, the robotic arm lifted the beaker and shook it to evenly disperse the solutes, thereby promoting dissolution. This heating and shaking process was repeated four times until the solutes were fully dissolved, forming a homogeneous ionic conductive solution. The robotic arm then returned the beaker to its original position. This task demonstrated the feasibility of using the robotic arm equipped with intrinsically decoupled dual-modal RoboTac skin to prepare ionic conductive solutions. In the future, through the implementation of automated control algorithms, fully automated chemical experiments based on RoboTac skin can be realized, expanding its application potential in laboratory chemical tasks.

Demonstration setup of RoboTac skin on human-robot interaction

One example of human-robot interaction is the robotic tea-making scenario. Two cups were prepared, one containing tea leaves and the other filled with hot water, and placed at different locations on a platform. After being equipped with RoboTac skin at the end of its gripper, the robotic arm was teleoperated to move to the hot water cup and perform a grasp. It then transferred the cup above the tea-leaf container and poured the hot water. After pouring, the empty cup was returned to its original position. Subsequently, the robotic arm grasped the cup, now containing brewed tea, and delivered it to the volunteer.

Another application of human-robot interaction is in medical robotics. RoboTac skin enables robots to achieve intrinsically decoupled dual-modal sensing capability. Owing to the high force resolution and rapid thermal response, RoboTac skin can be used to measure both the wrist pulse and the surface skin temperature. When deployed on the gripper of a robotic arm, the system can monitor a volunteer's real-time skin temperature and pulse signals via an external display. The gripper was positioned to press on the radial artery of the volunteer's wrist to capture the relevant signals. Measurements were performed for approximately one minute under two conditions, i.e., at rest and after physical exertion.

Supplementary Figures

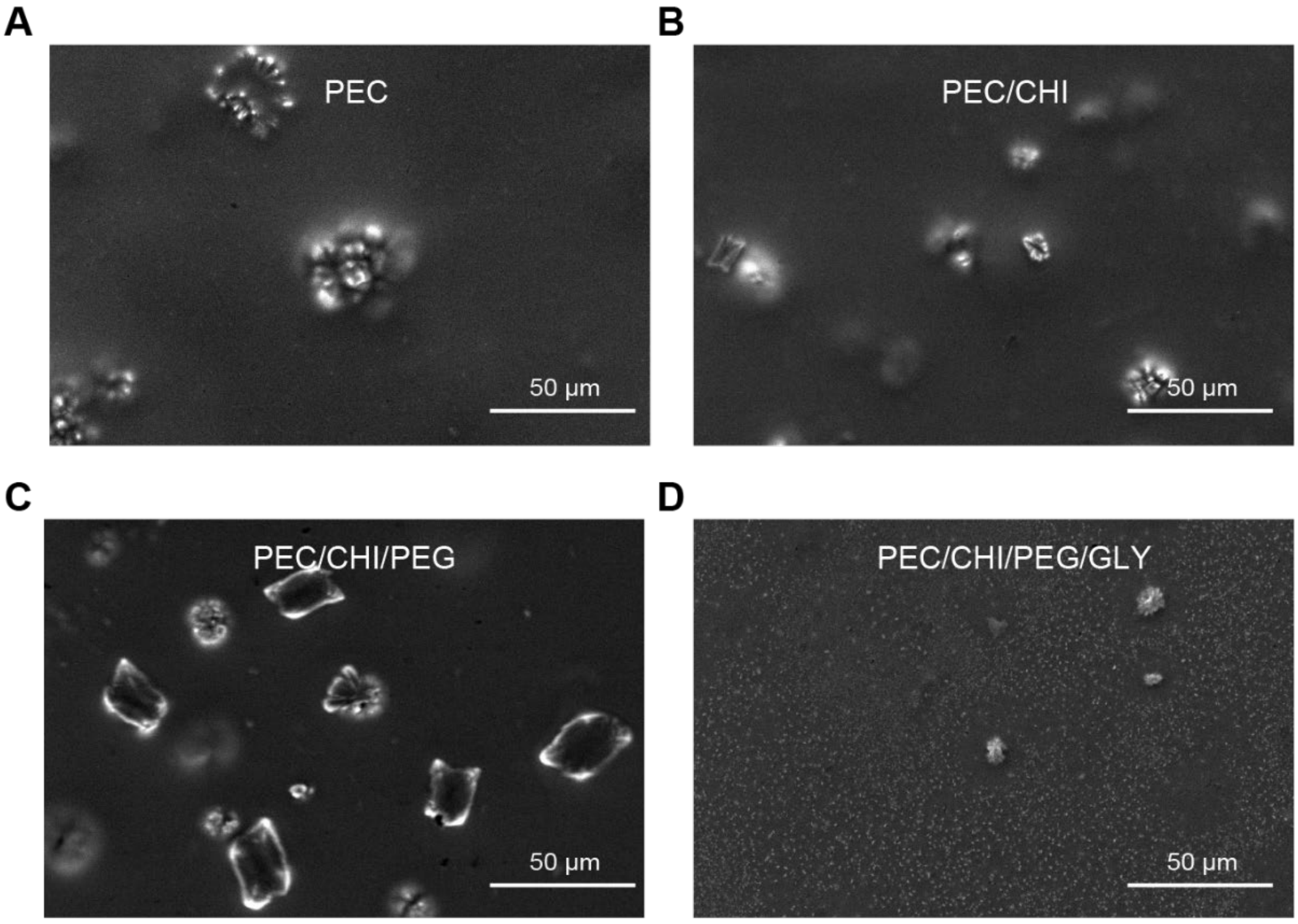

Fig. S1. SEM photos of the surface of four ionic conductive films.

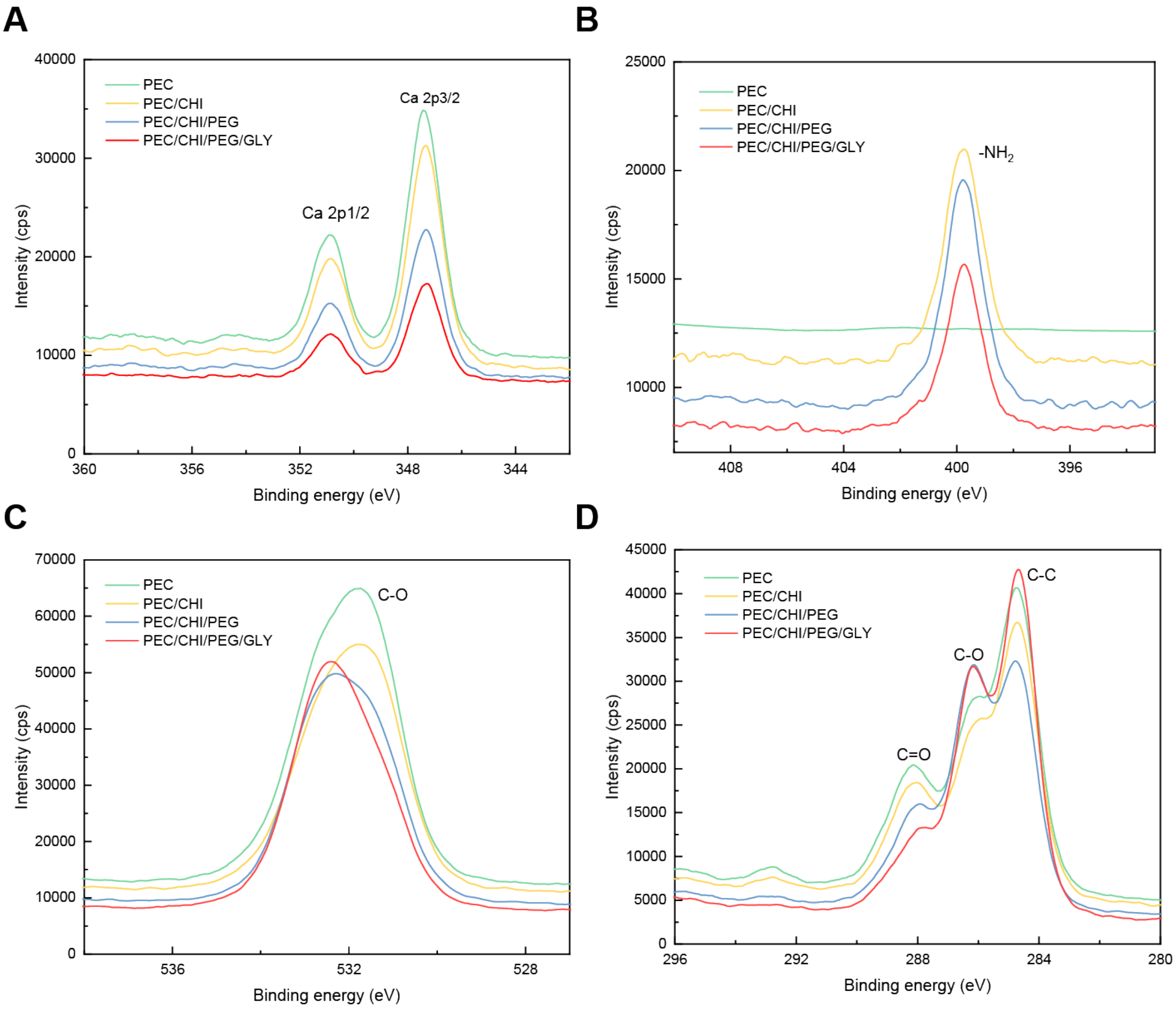

Fig. S2. XPS spectra of the ionic conductive films regarding different elements. (A) XPS spectra of calcium. (B) XPS spectra of nitrogen element. (C) XPS spectra of the oxygen element. (D) XPS spectra of the carbon element.

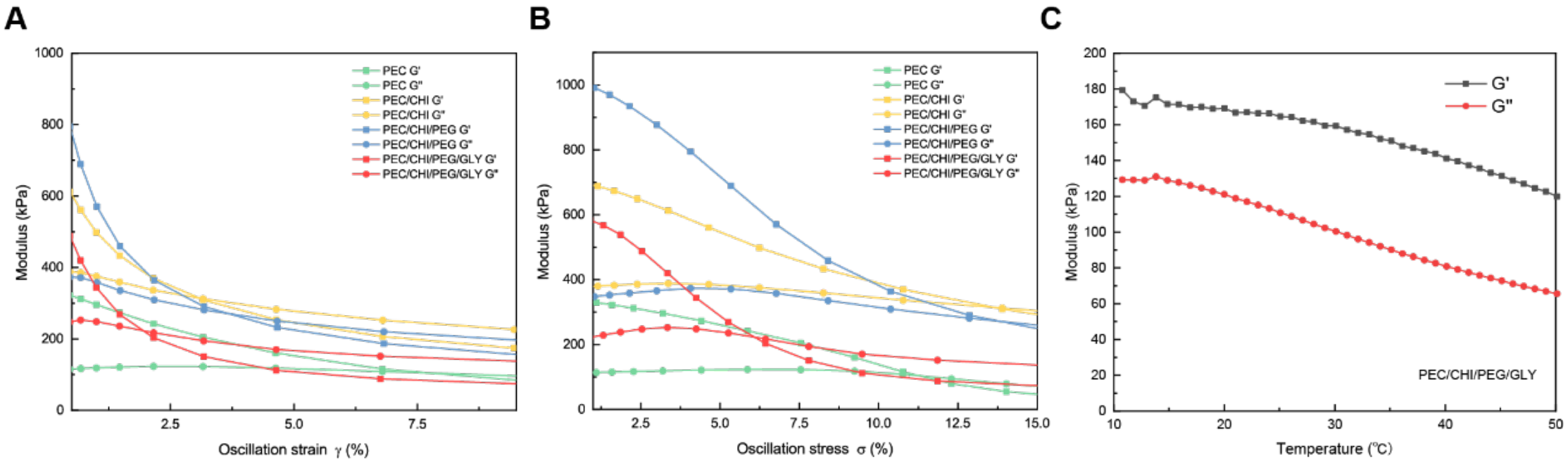

Fig. S3. Rheological characterizations of the ionic conductive films. (A) Relationship between the oscillation strain and the storage modulus and loss modulus. (B) Relationship between the oscillation stress and the storage modulus and loss modulus. (C) Relationship between the temperature (10-50 °C) and the storage modulus and loss modulus of the PEC/CHI/PEG/GLY film.

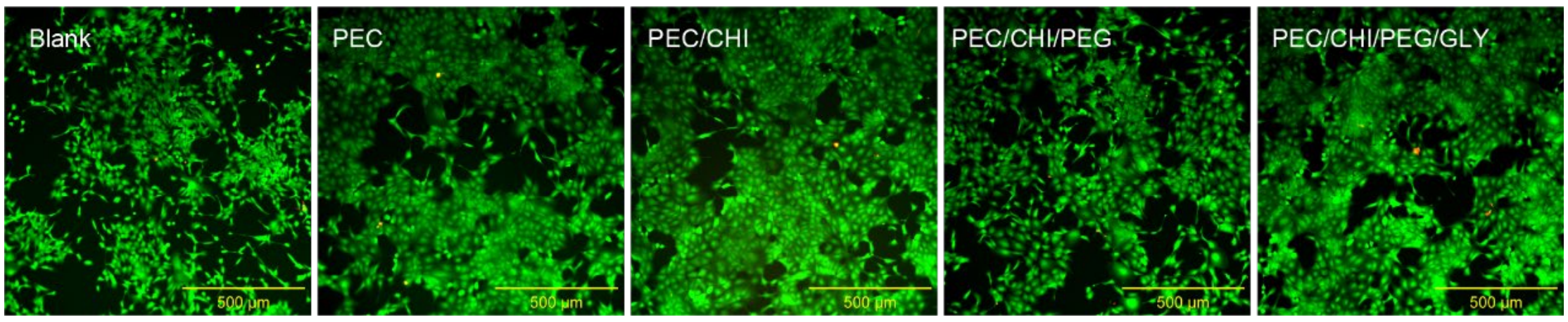

Fig. S4. Fluorescence images of the cultured cells under ionic conductive films. The results show that all ionic conductive films exhibit low cytotoxicity, demonstrating their good biocompatibility.

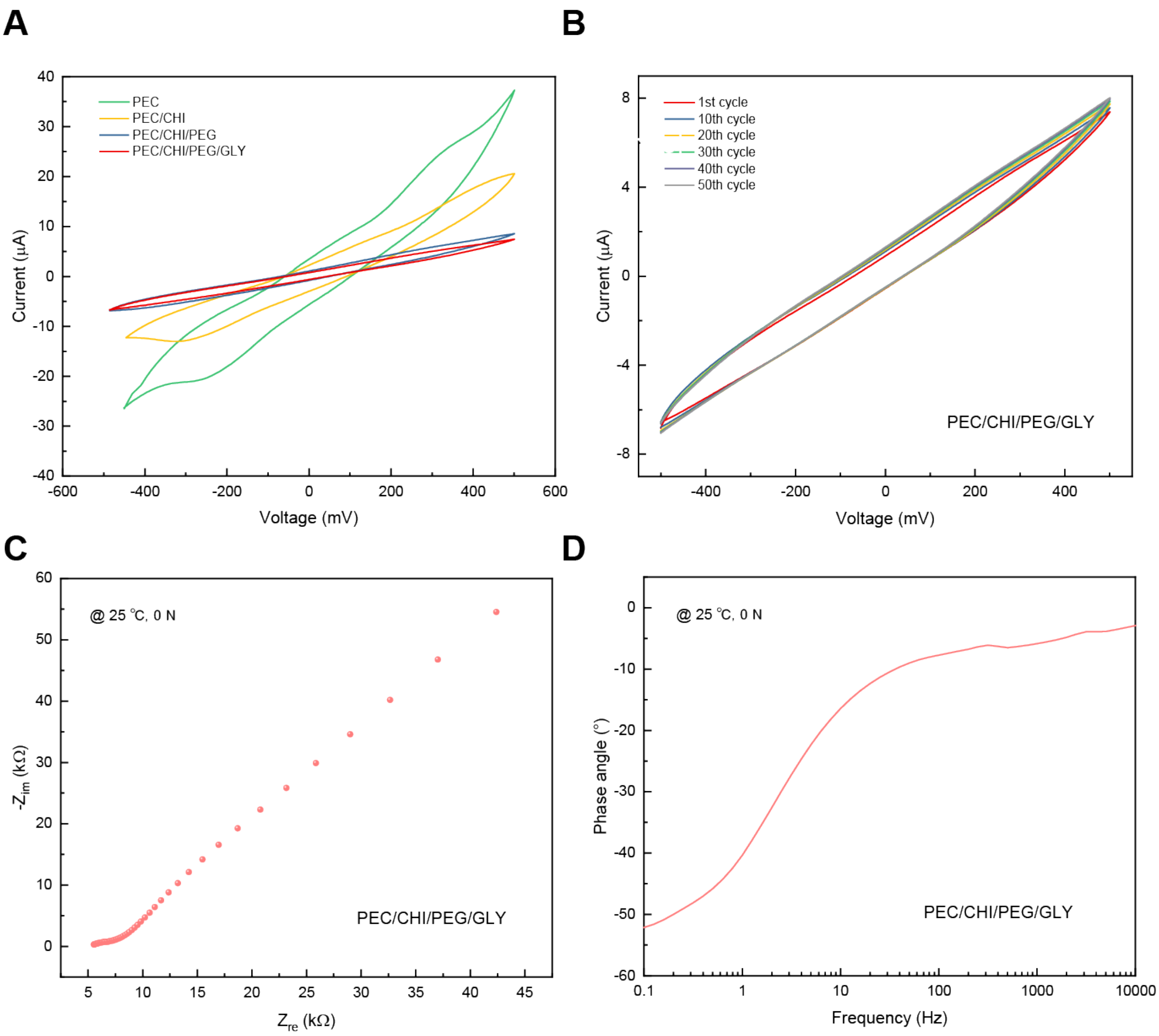

Fig. S5. Electrochemical characteristics of the ionic conductive films. (A) Cyclic voltammetry curves. (B) Multiple cyclic voltammetry curves of the PEC/CHI/PEG/GLY film. (C) The Nyquist characteristic of the PEC/CHI/PEG/GLY film. (D) Phase angle measurement of the PEC/CHI/PEG/GLY film as a function of frequency.

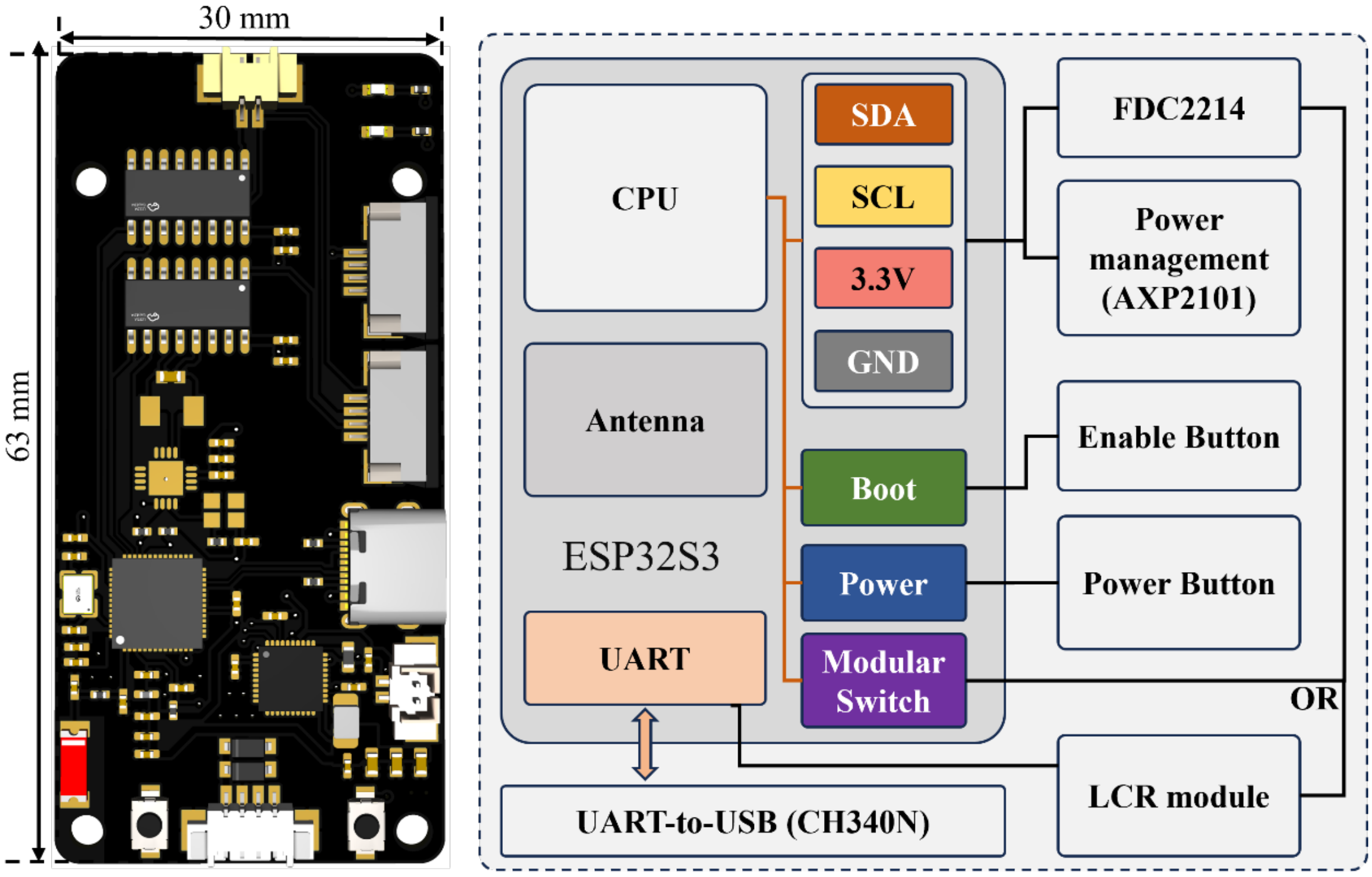

Fig. S6. Schematic of the electronic system of RoboTac skin. RoboTac skin's electronic system consists of an MCU module, a capacitive measurement module, and an impedance measurement module. The main controller board includes the MCU with integrated Wi-Fi/BLE, a power management unit, two buttons, a mode selection switch, a USB-to-UART interface, and two signal acquisition modules.

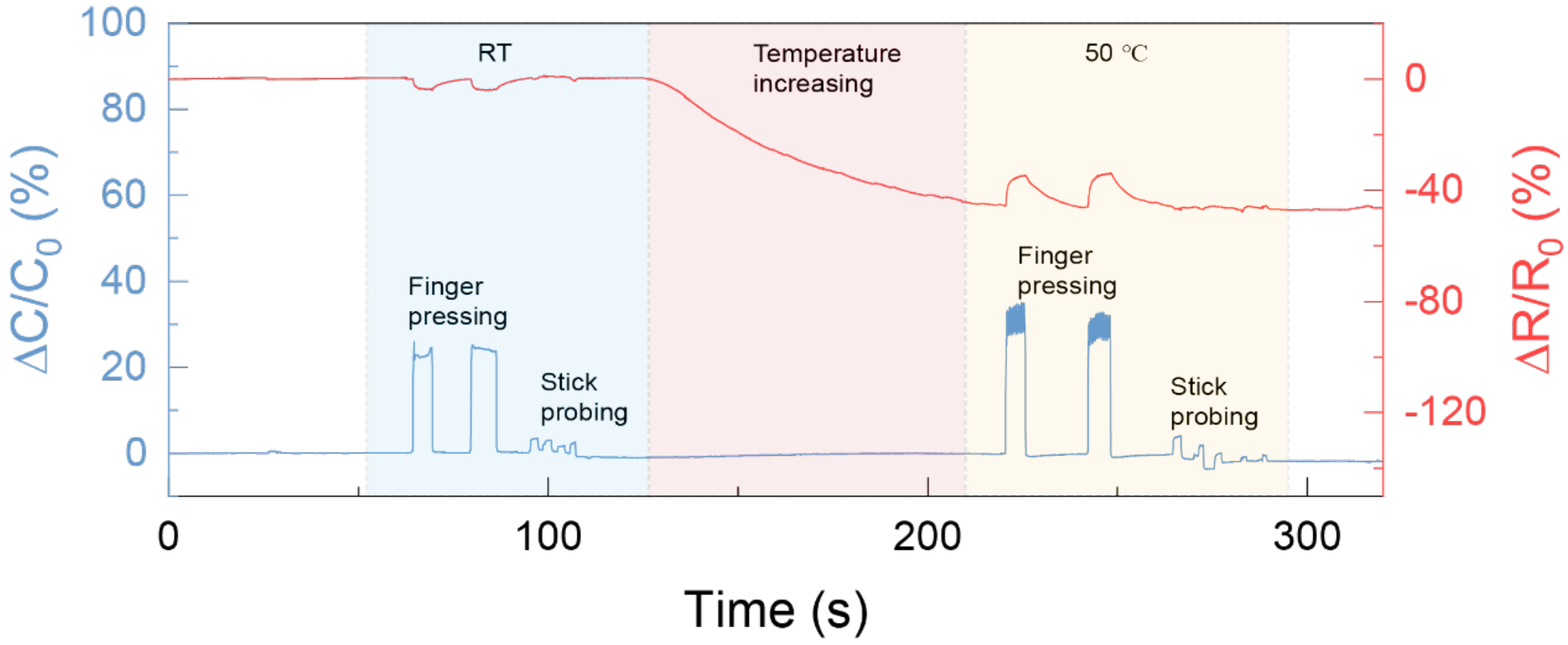

Fig. S7. Experiment for validation of the intrinsically self-decoupled sensing (corresponding to Movie S4). By applying both RT and non-RT forces to RoboTac skin under different environmental temperatures, we found that forces at RT did not alter its resistive response, whereas non-RT forces did. In addition, changing the environmental temperature of RoboTac skin did not affect its capacitive response. These findings further confirm the intrinsically self-decoupled dual-modal sensing capability of RoboTac skin.

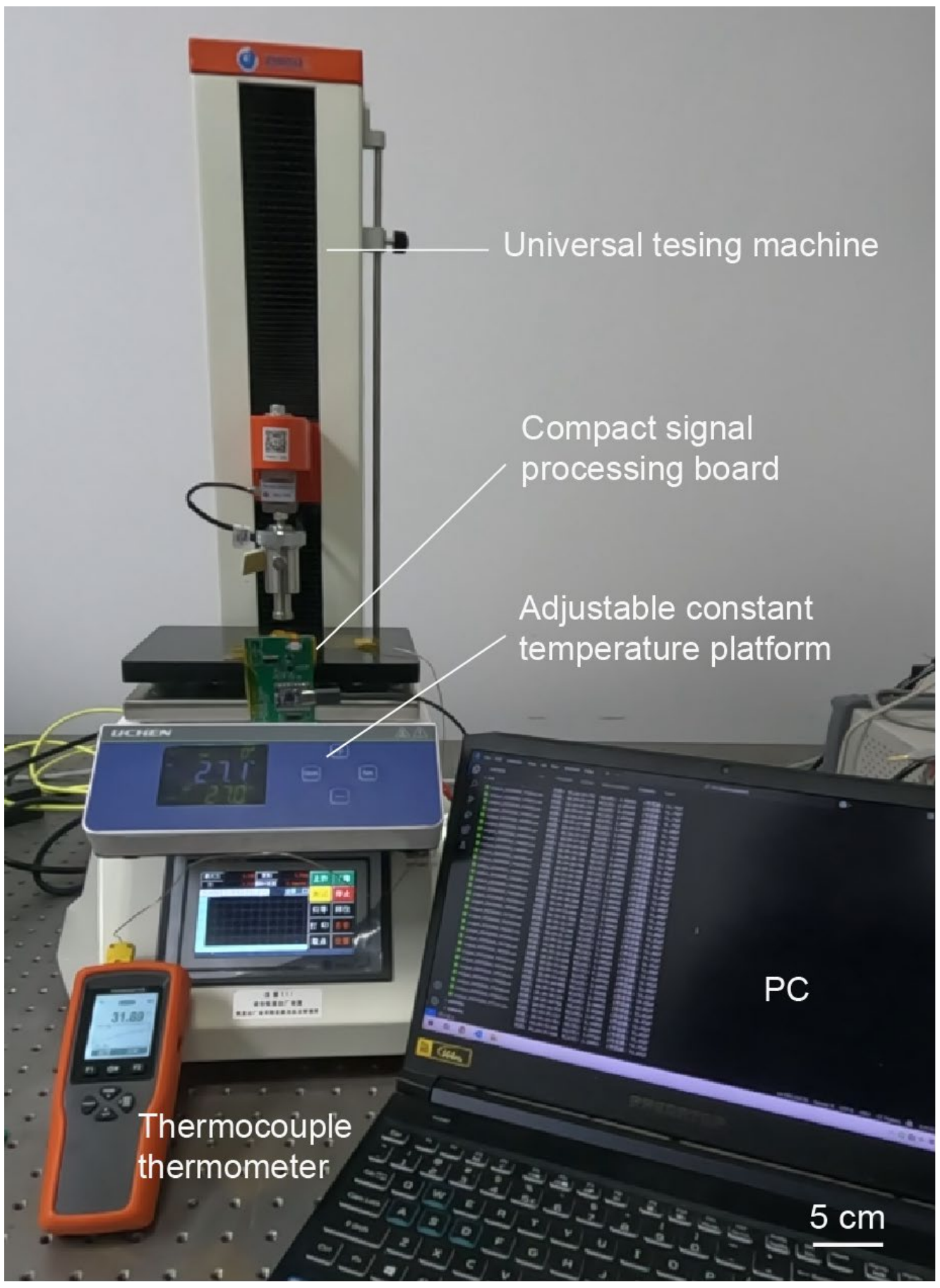

Fig. S8. Optical photo of the dual-modal experimental platform. The dual-modal calibration setup consists of a universal testing machine, an adjustable constant temperature platform, and a thermocouple thermometer. RoboTac skin is connected via an FPC to a compact signal processing board, which processes and transmits the data to a PC.

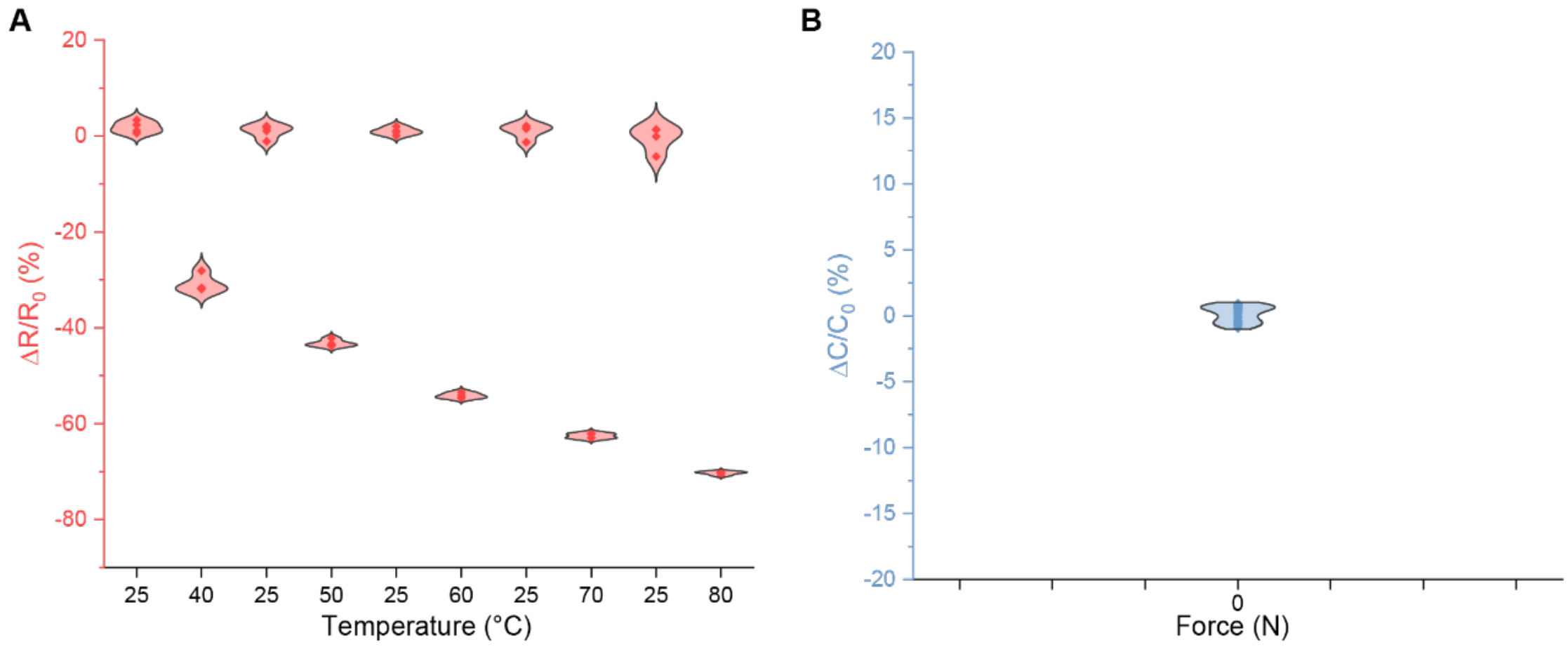

Fig. S9. Error of the dynamic response of RoboTac skin to varied temperatures. The results show that RoboTac skin exhibits minimal error in temperature response over multiple temperature cycling tests, indicating good consistency in temperature measurement. Meanwhile, the force response also shows minimal error, confirming the decoupling of force measurement from temperature variations.

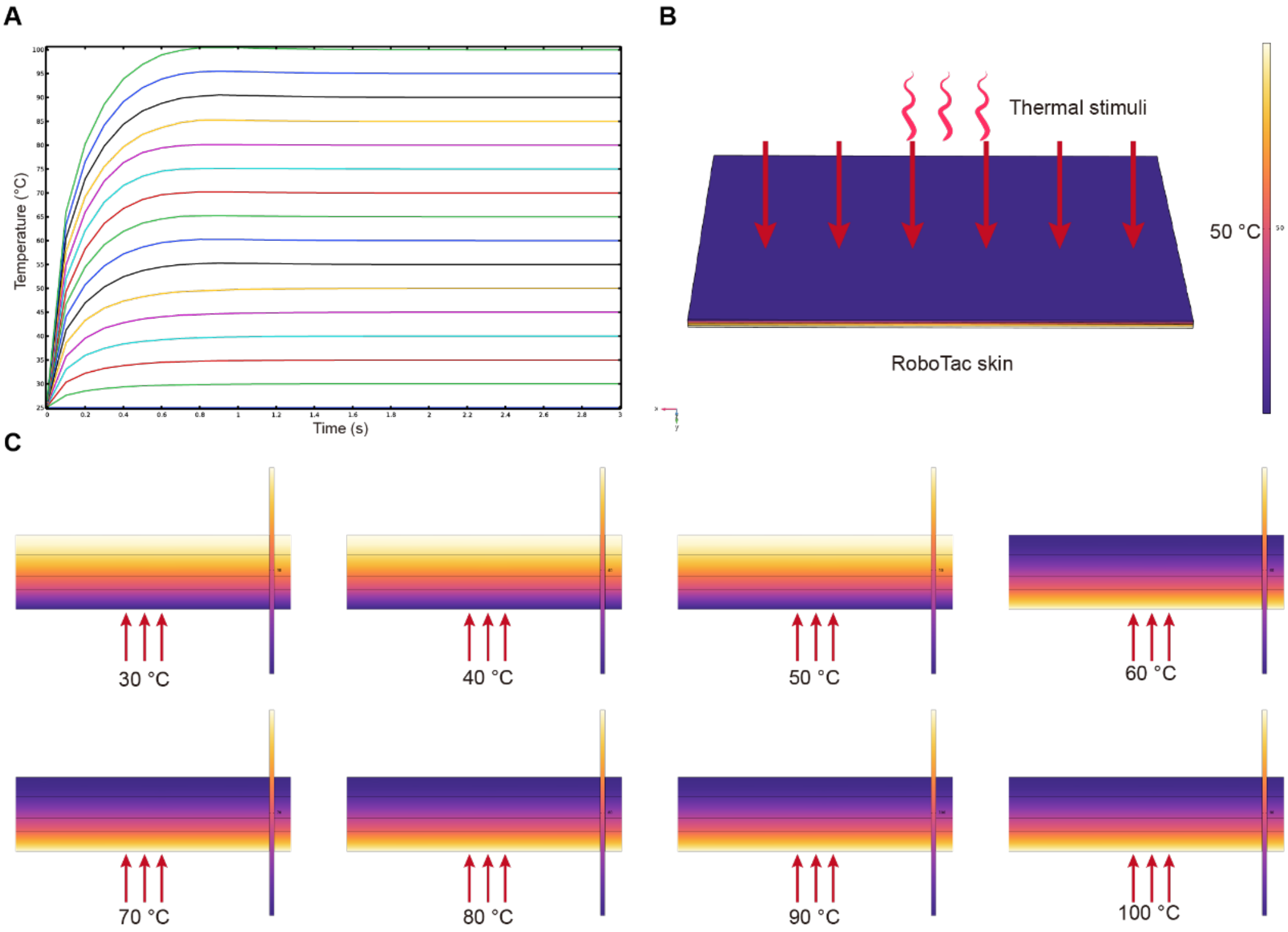

Fig. S10. Steady-state heat transfer simulation of RoboTac skin. (A) Dynamic temperature response of the sensing layer upon various thermal stimuli (From 25 °C to 100 °C, in 5 °C increments). (B) Illustration of the steady-state heat transfer simulation. (C) Results of steady-state heat transfer simulation (30 °C, 40 °C, 50 °C, 60 °C, 70 °C, 80 °C, 90 °C, 100 °C).

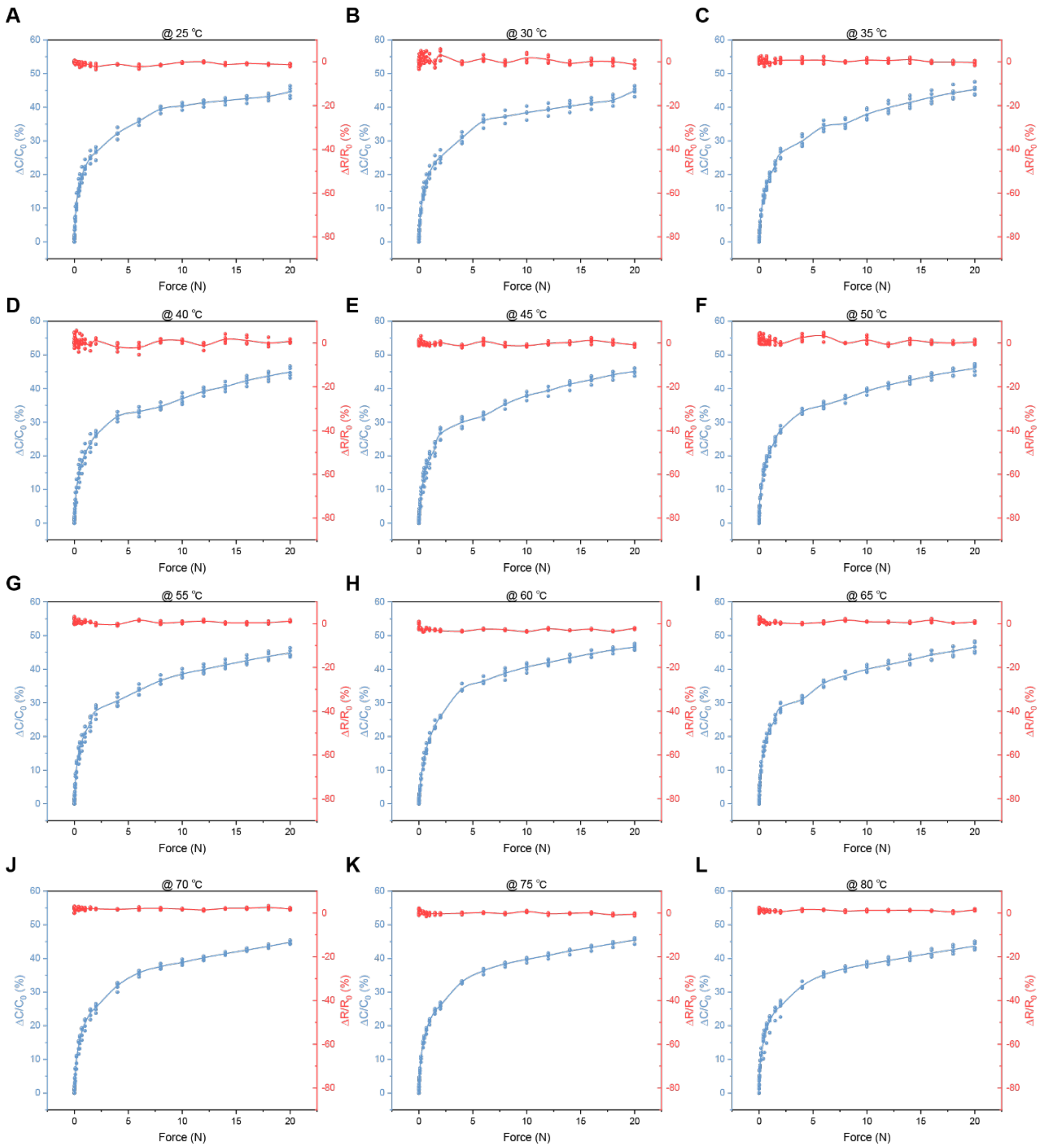

Fig. S11. Relationship between RoboTac skin's response and force under various thermal conditions (from 25 °C to 80 °C, in 5 °C increments). By calibrating the force signal response of RoboTac skin five times at different temperatures, we found that the capacitive responses measured at all twelve temperature conditions exhibited excellent consistency, while the resistive responses remained nearly unchanged. This indicates effective decoupling of temperature sensing from force sensing.

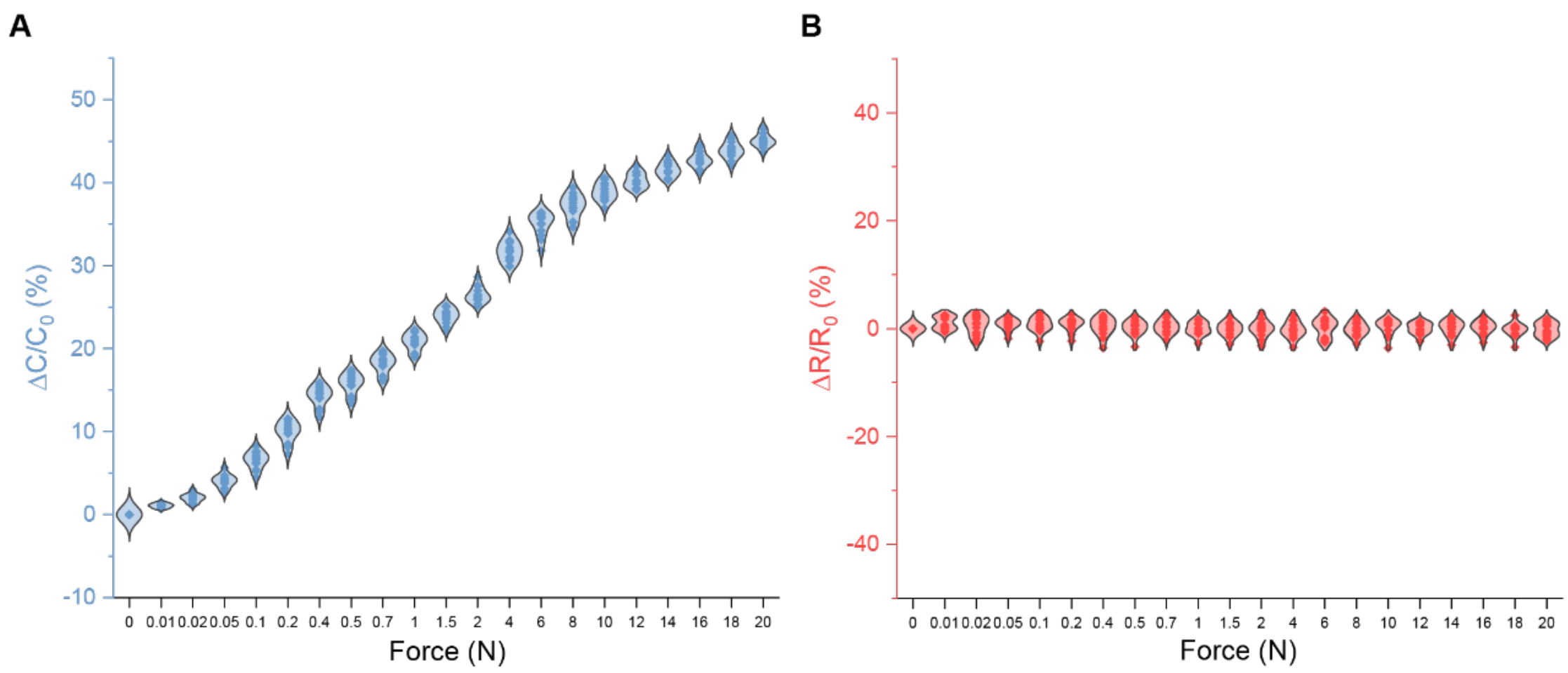

Fig. S12. Error of the dynamic response of RoboTac skin to varied temperatures when calibrating the force sensing ability. When different forces are applied to RoboTac skin under varying temperatures, the force response shows minimal error, indicating good consistency in force measurement. At the same time, the temperature response also exhibits low error, confirming the decoupling of temperature measurement from applied force.

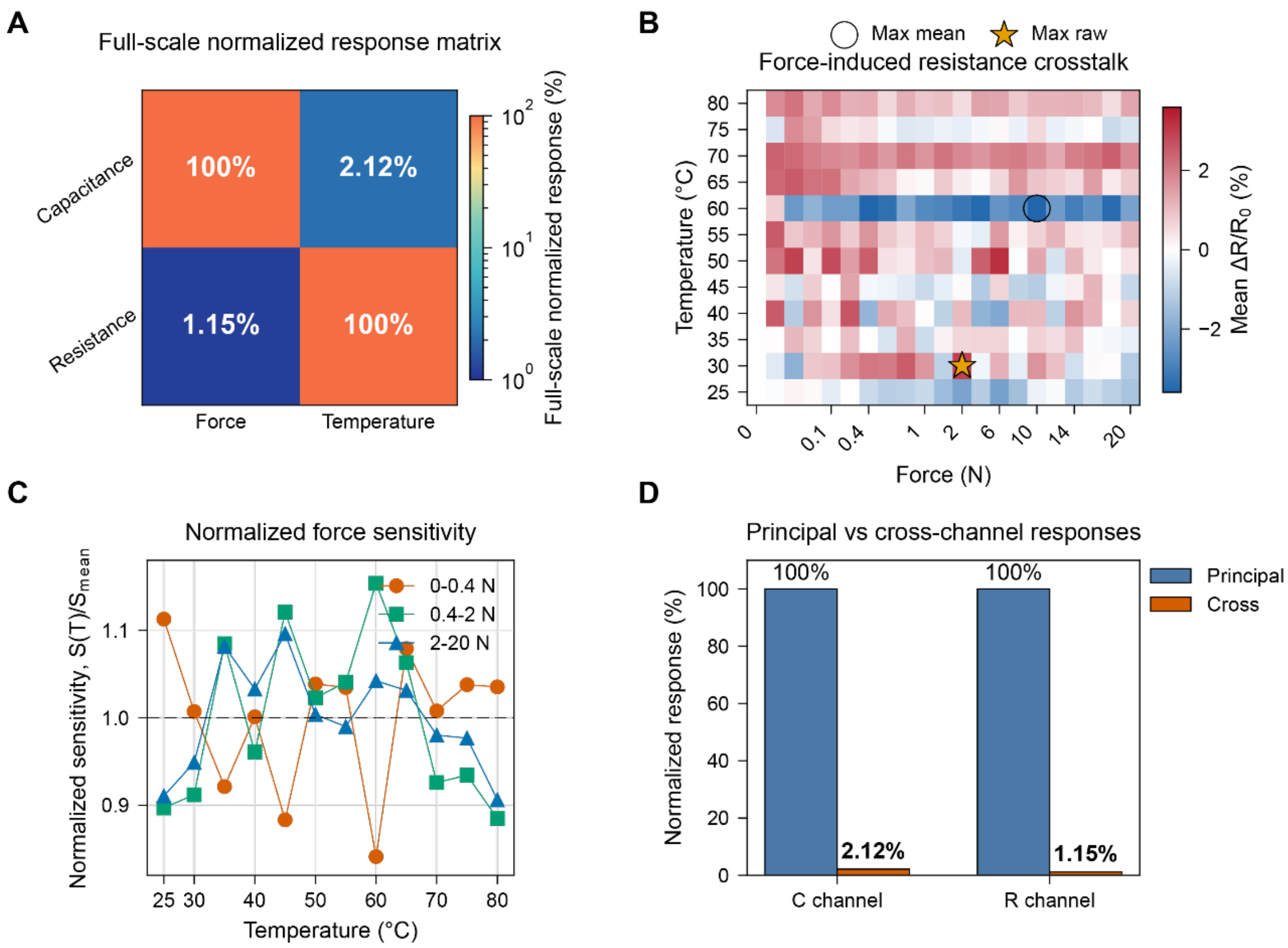

Fig. S13. Quantitative evaluation of the cross-sensitivity of RoboTac skin. (A) Full-scale normalized response matrix showing the principal force-to-capacitance and temperature-to-resistance responses and the corresponding cross-channel responses. The principal responses were normalized to 100%, while the temperature-to-force and force-to-temperature crosstalk ratios were 2.12% and 1.15%, respectively. (B) Heatmap of the replicate-averaged force-induced resistance variation under forces of 0-20 N and temperatures of 25-80 °C. The maximum replicate-averaged variation was 3.595% at 60 °C and 10 N, while the maximum raw variation was 5.834% at 30 °C and 2 N. (C) Normalized force sensitivities in the three force ranges as a function of temperature. (D) Comparison of the full-scale principal and cross-channel responses of the capacitance and resistance channels.

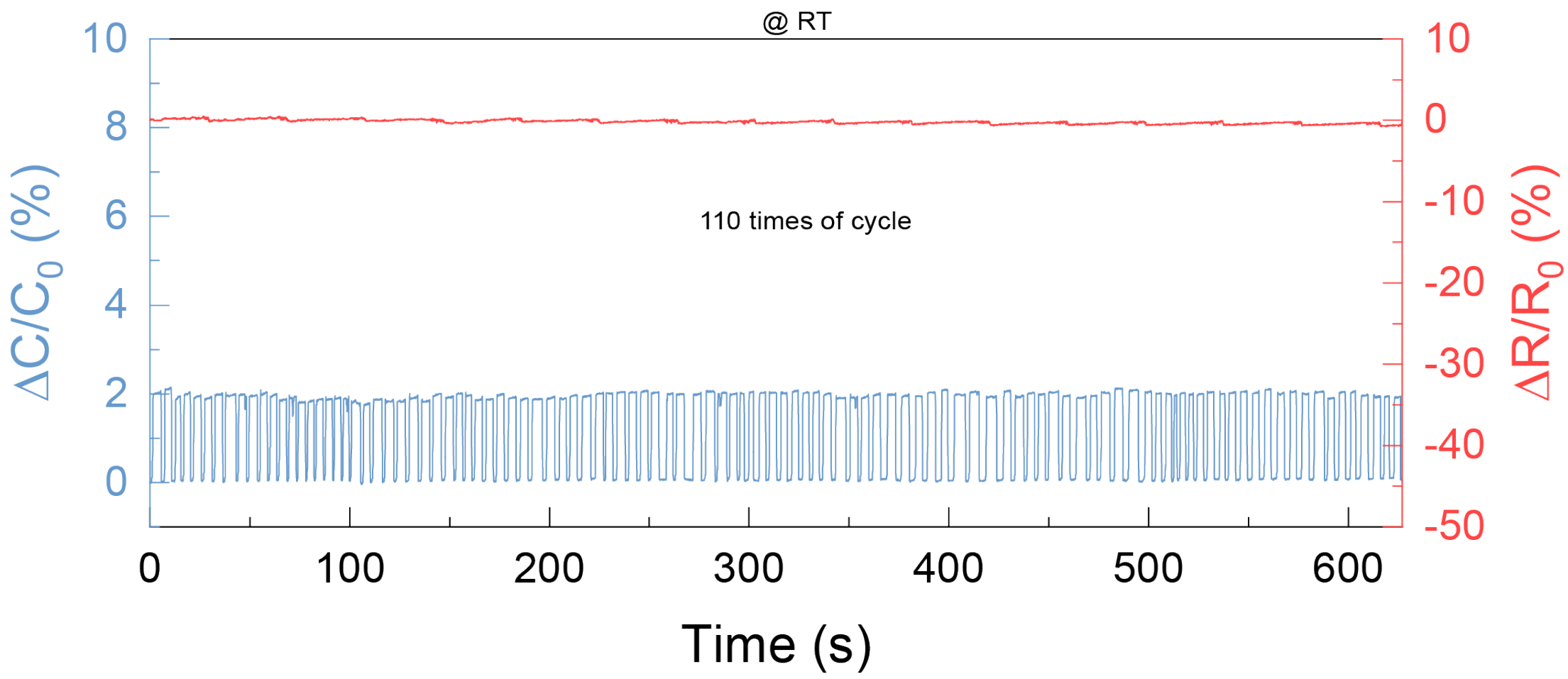

Fig. S14. Repeatable response (110 cycles) of RoboTac skin (corresponding to Movie S5). After 110 repeated applications of external force, the RoboTac skin continued to measure accurate capacitive signals with high consistency across responses, while the resistive signal remained unchanged. This result demonstrates the repeatability of RoboTac skin's measurements and further confirms its intrinsically self-decoupled dual-modal sensing capability.

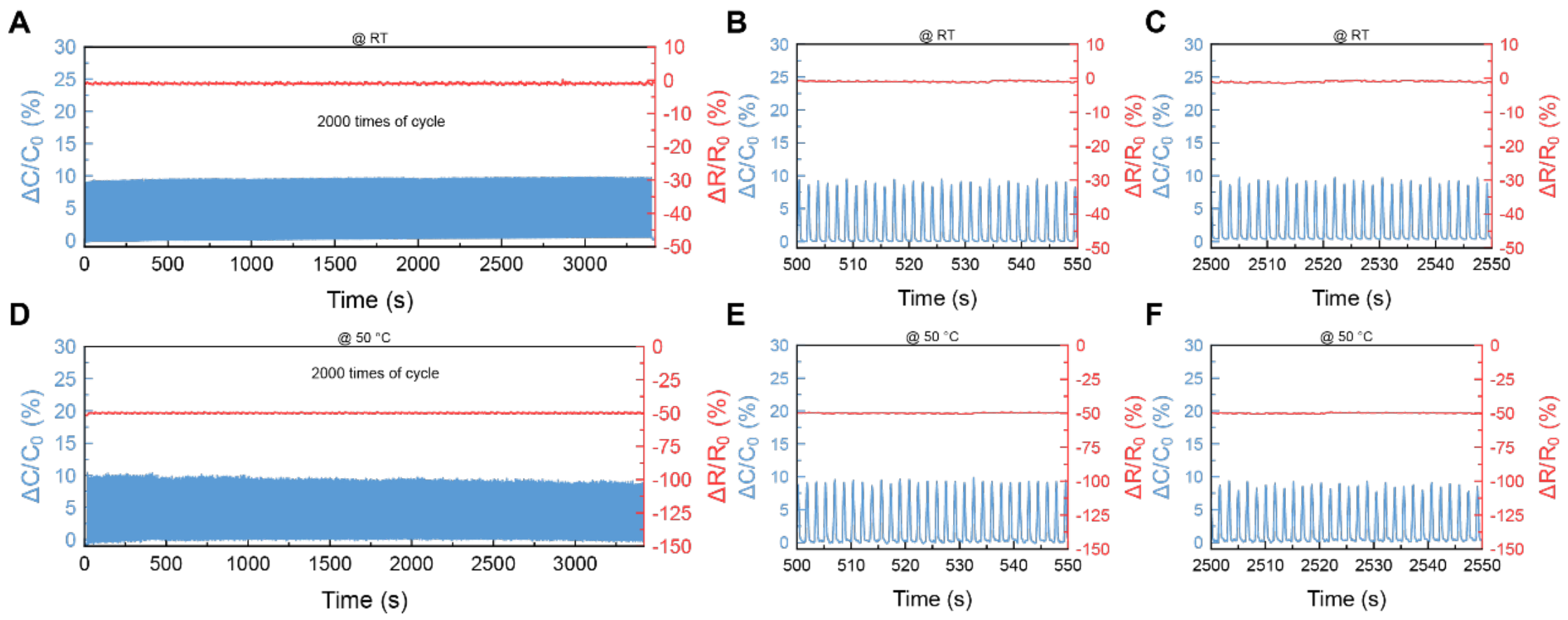

Fig. S15. Cycle test of RoboTac skin. Responses during 2000 loading-unloading cycles under two representative temperature-pressure conditions, together with enlarged views of the initial and final cycles. (A) Capacitance and resistance responses during 2000 loading-unloading cycles under room temperature. (B) Enlarged responses of the initial cycles under room temperature. (C) Enlarged responses of the final cycles under room temperature. (D) Capacitance and resistance responses during 2000 loading-unloading cycles under 50 ℃. (E) Enlarged responses of the initial cycles under 50 ℃. (F) Enlarged responses of the final cycles under 50 ℃.

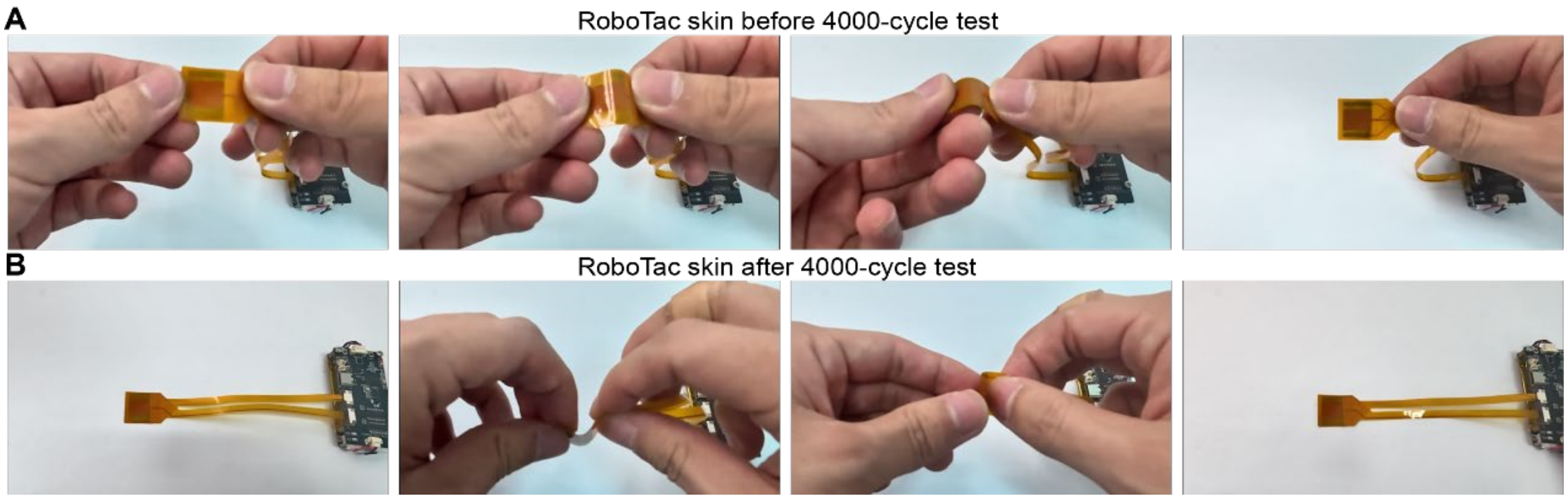

Fig. S16. Structural and encapsulation integrity of RoboTac skin before and after prolonged cyclic testing. Optical photographs of RoboTac skinbefore and after the two 2000-cycle loading-unloading tests conducted at room temperature and 50 °C, corresponding to 4000 cycles in total (duration time over 120 min). Manual bending and twisting were subsequently applied to evaluate the integrity of the internal PI-tape bonding. No visible edge lifting, interlayer delamination, bubble formation, or tape detachment was observed after cyclic testing or manual deformation.

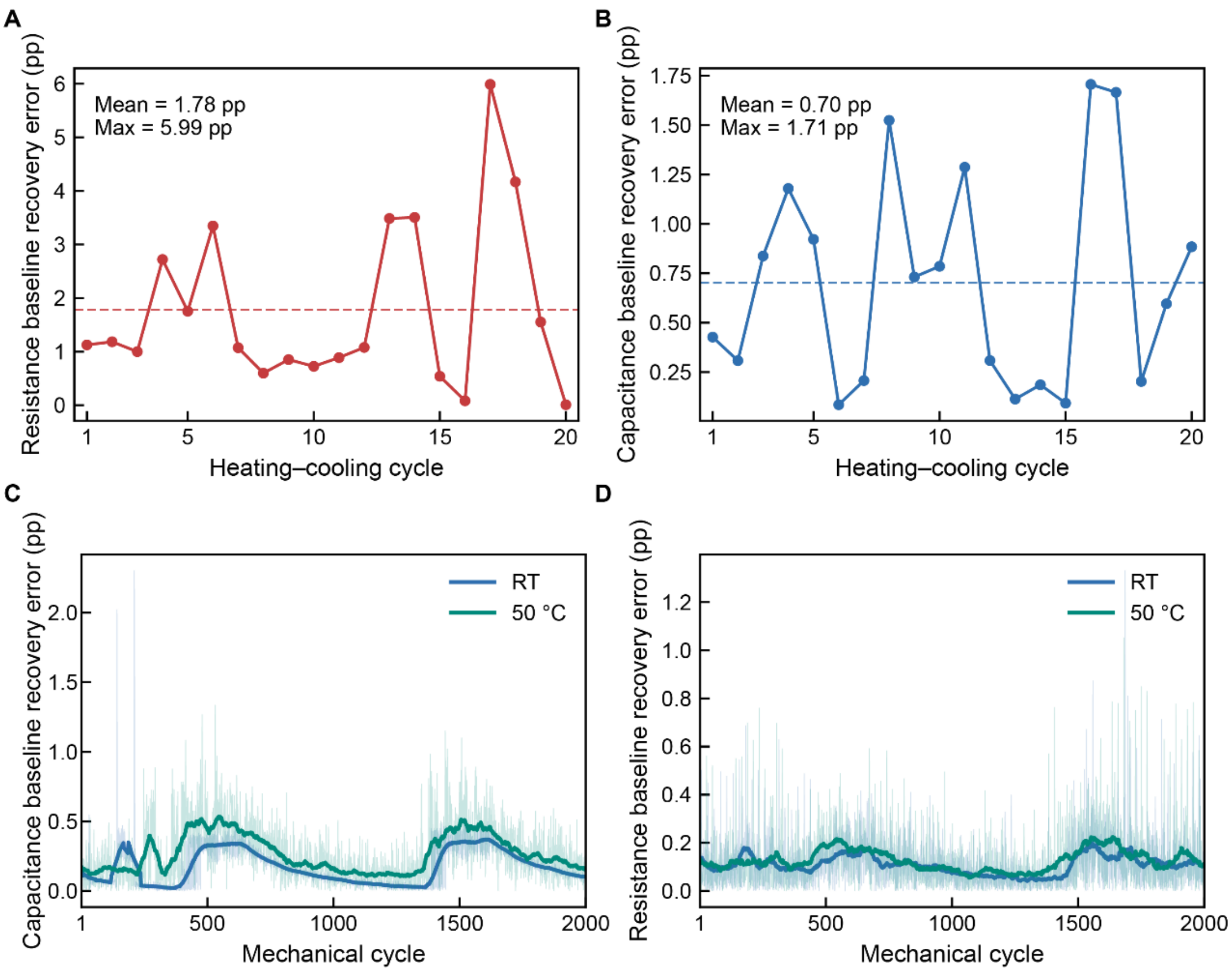

Fig. S17. Quantitative evaluation of baseline recovery under repeated thermal and mechanical stimuli. (A, B) Resistance and capacitance baseline recovery errors, respectively, after each heating-cooling cycle. (C, D) Capacitance and resistance baseline recovery errors, respectively, during 2000 mechanical loading-unloading cycles at room temperature and 50 °C. The dashed lines in (A) and (B) indicate the mean recovery errors. The thin lines in (C) and (D) represent the cycle-by-cycle values, while the thick lines represent the corresponding 50-cycle moving averages. Since the original signals were normalized as $\Delta C/C_0$ (%) and $\Delta R/R_0$ (%), the recovery errors are reported as absolute differences in percentage points (pp). Rigorous hysteresis loops were not extracted because synchronized continuous force and temperature trajectories were unavailable.

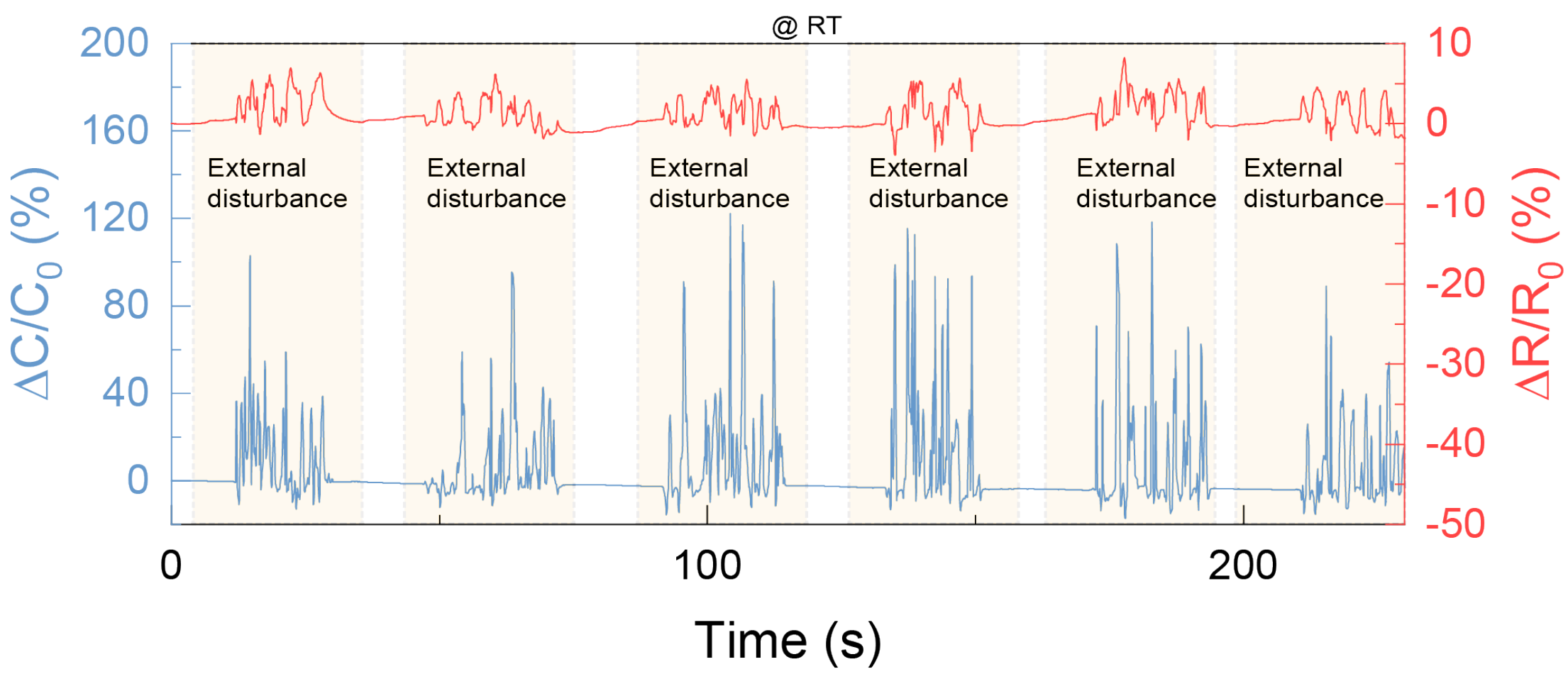

Fig. S18. Dynamic response of RoboTac skin when external disturbance (6 times) was applied repeatedly (corresponding to Movie S6). When RoboTac skin is subjected to external disturbances such as manual folding, twisting, bending, and stretching, both of its sensing modalities exhibit corresponding variations. Once the disturbance is removed, the signals returned to their baseline values, demonstrating the strong interference resistance of the RoboTac skin. This robustness paves the way for its stable deployment on robotic end-effectors to perform practical tasks.

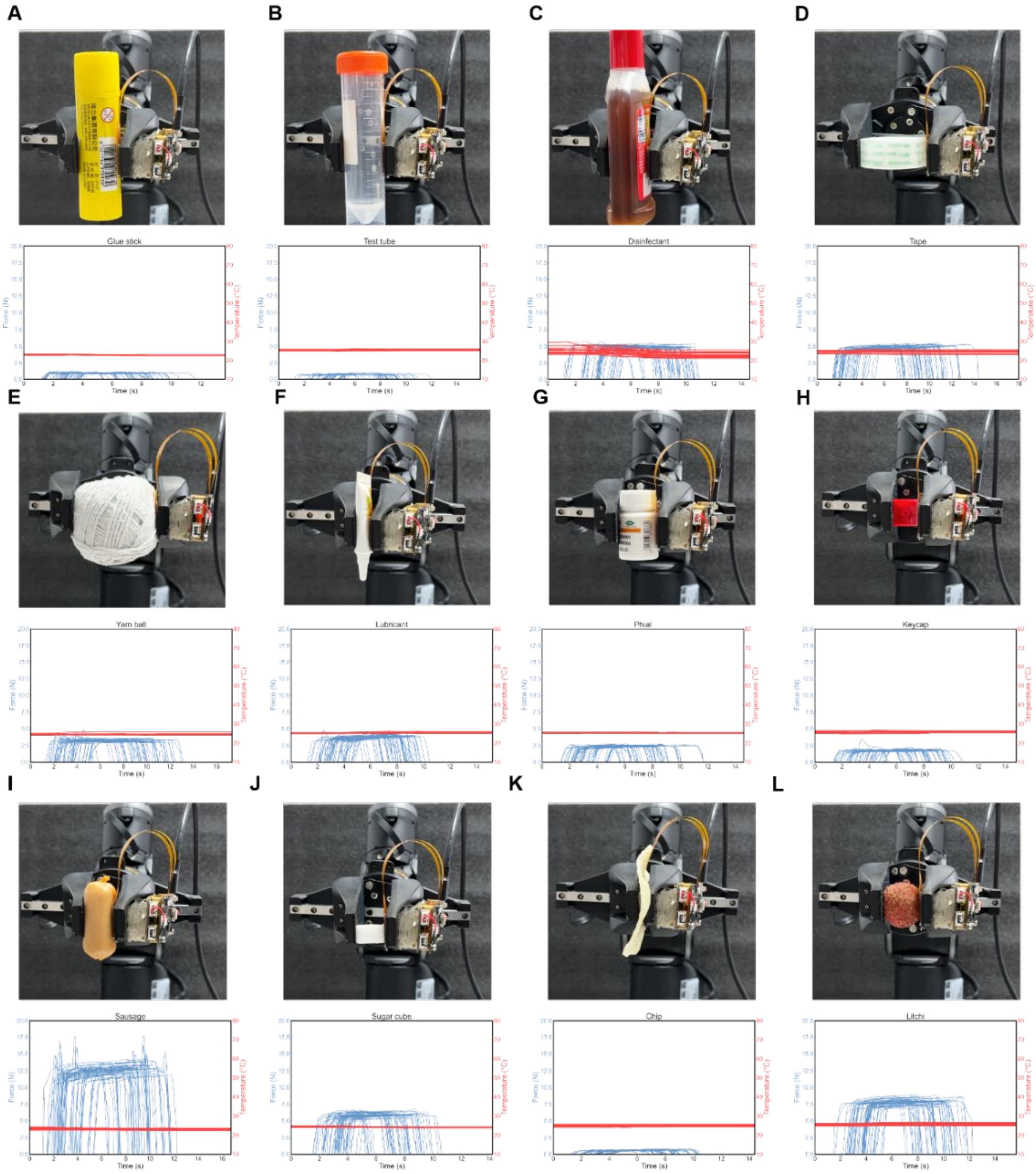

Fig. S19. Dynamic response of RoboTac skin when the robot grasped various objects. These objects include (A) glue stick, (B) test tube, (C) disinfectant, (D) tape, (E) yarn ball, (F) lubricant, (G) phial, (H) keycap, (I) sausage, (J) sugar cube, (K) chip, and (L) litchi. Through teleoperation, dual-modal signals were collected for 50 trials from each of the twelve objects. Variations in the force signals were observed due to differences in the objects' volume and stiffness, whereas the temperature signals for all twelve objects remained close to RT since they were tested under ambient conditions. These results further confirm the intrinsically self-decoupled dual-modal sensing capability of RoboTac skin.

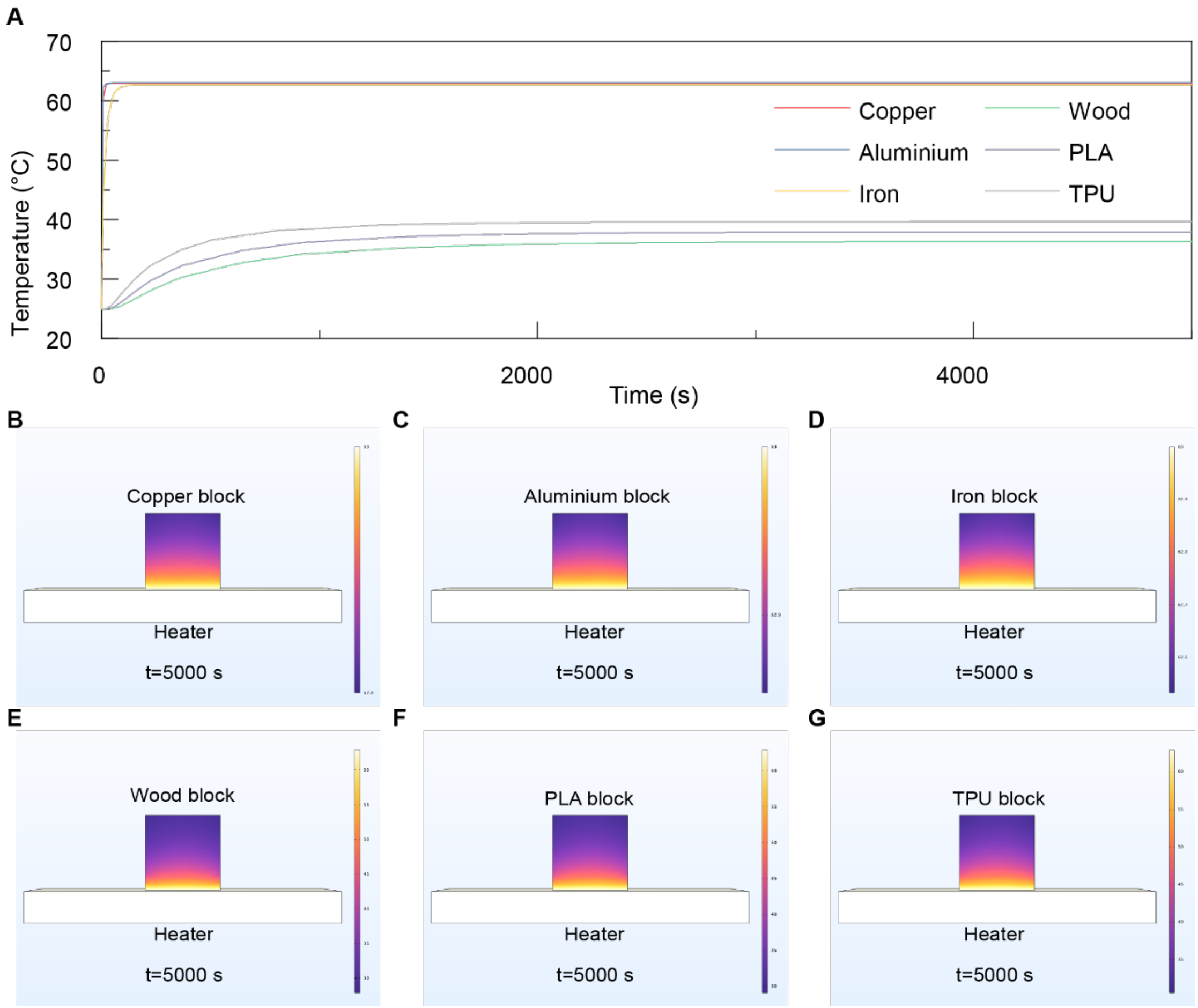

Fig. S20. Simulation results of the steady-state temperature of objects heated by a heat source. (A) Temperature variation curves of different objects reaching steady state. The temperature at the steady state of different objects, including (B) copper, (C) aluminum, (D) iron, (E) wood, (F) PLA, and (G) TPU. Temperature variation curves of different objects reaching steady state. The steady-state temperatures of different materials vary. Copper, aluminum, and iron reach similar steady-state temperatures around 63 °C, while wood, PLA, and TPU stabilizes at a lower temperature of approximately 37 °C. This difference is mainly due to the variations in specific heat capacity at constant pressure. Materials with higher volumetric heat capacity and lower thermal conductivity exhibit slower heating and may reach lower temperatures within a finite heating period under convective heat loss.

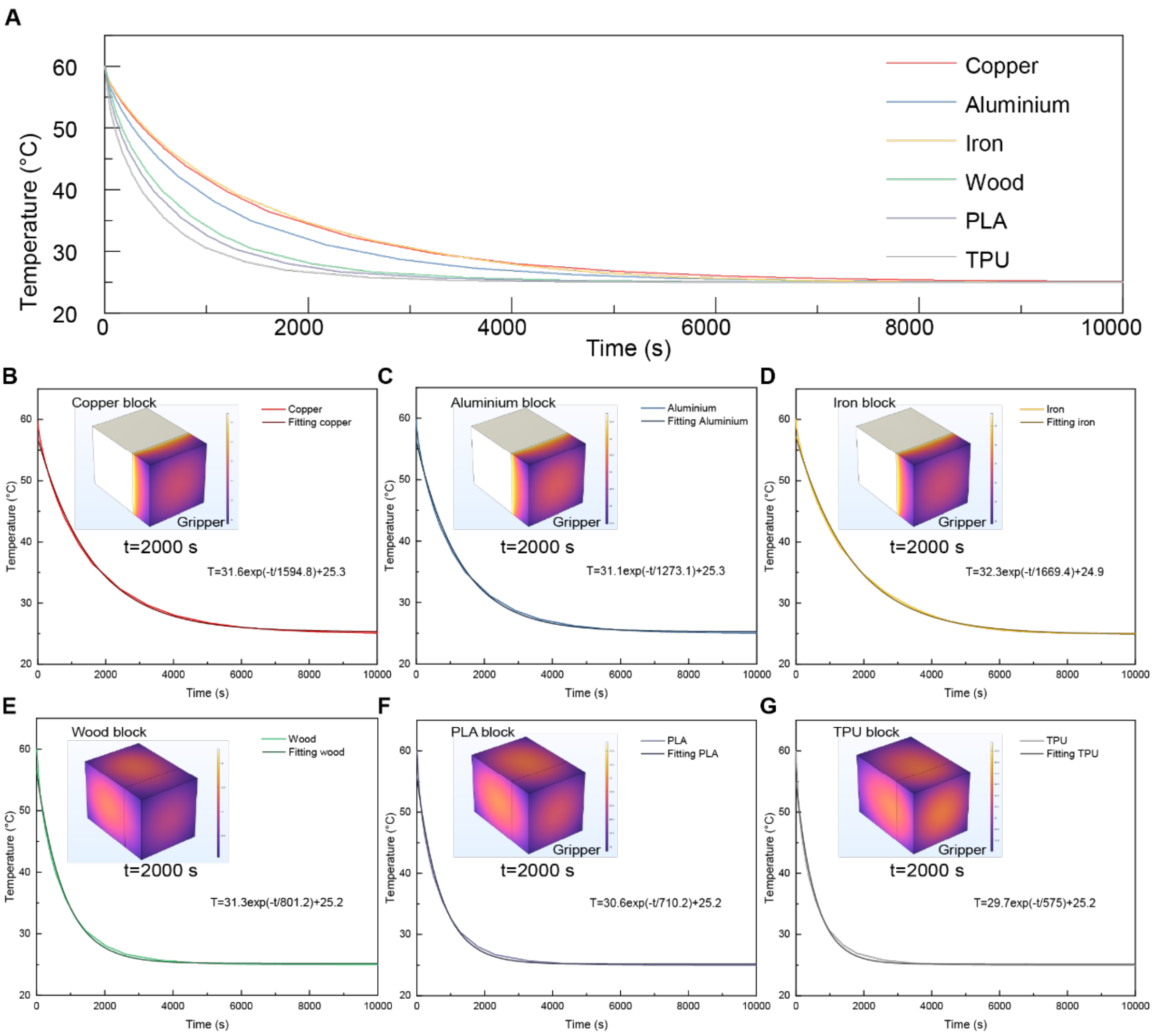

Fig. S21. Simulation results of the transient heat conduction of different objects. (A) The temperature change during the transient heat conduction process of different objects, including (B) copper, (C) aluminum, (D) iron, (E) wood, (F) PLA, and (G) TPU. Simulation results show that during transient heat conduction in air, the cooling rates of TPU, PLA, wood, aluminum, copper, and iron decrease in sequence. Although the thermal conductivities of copper, aluminum, iron, TPU, PLA, and wood decrease in that order, the observed cooling trend is influenced by the heat capacity of the materials, which also plays a critical role in transient heat transfer through air.

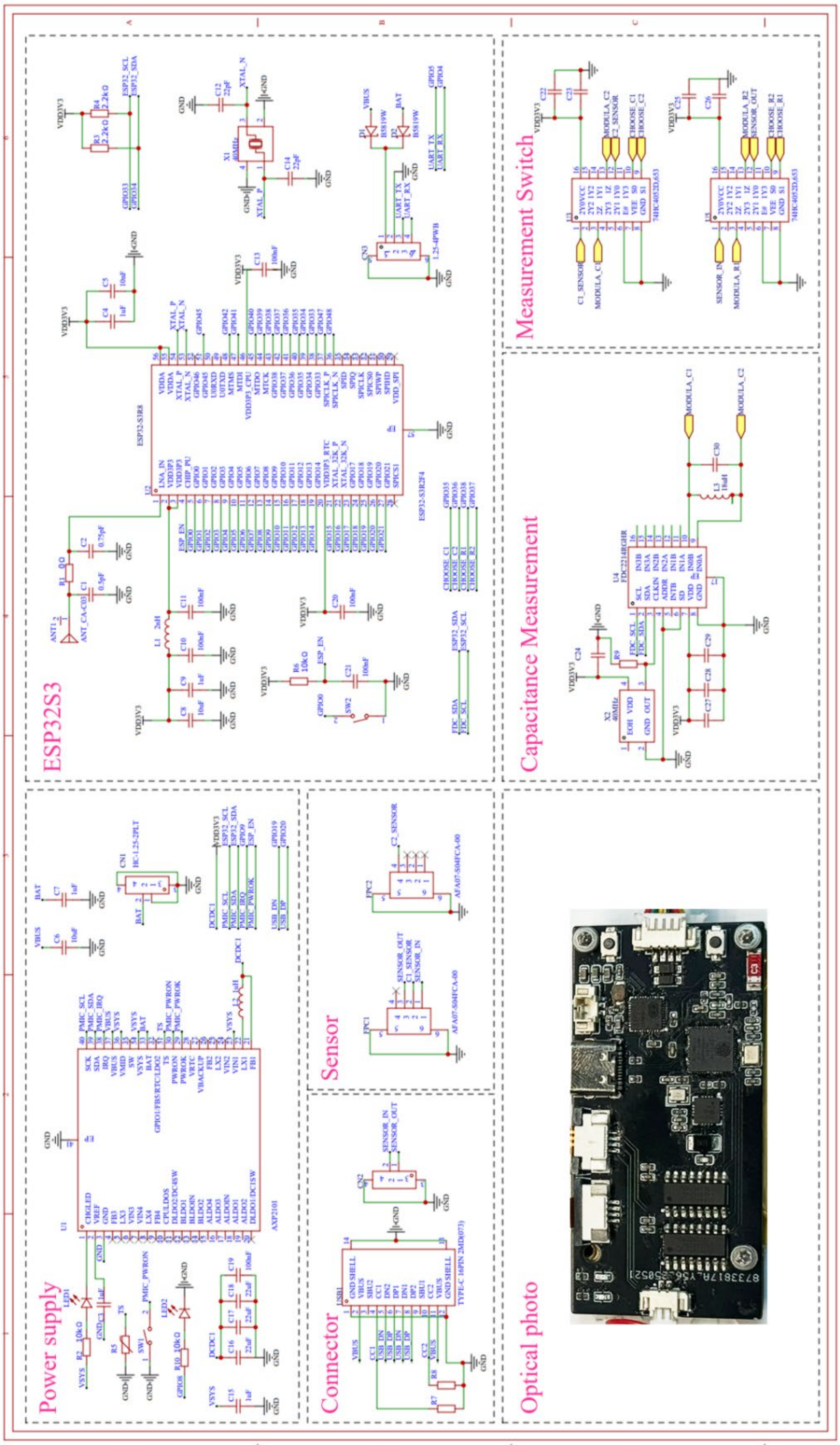

Fig. S22. Circuit diagram of the electronic system of RoboTac skin. The circuit comprises six main components: a power supply unit, a connector interface, sensor electrodes, the ESP32 controller, a capacitive measurement module, a measurement switch, and an external commercial LCR module.

Supplementary References

1. W. Li, C. Lin, A. Rasheed, E. Iranmanesh, Q. Zhou, K. Wang, A Force and Temperature Sensor Array Based on 3-D Field-Coupled Thin-Film Transistors for Tactile Intelligence. *IEEE Trans. Electron Devices* **67**, 2890-2895 (2020).

2. W. Yang, M. Xie, X. Zhang, X. Sun, C. Zhou, Y. Chang, H. Zhang, X. Duan, Multifunctional Soft Robotic Finger Based on a Nanoscale Flexible Temperature-Pressure Tactile Sensor for Material Recognition. *ACS Appl. Mater. Interfaces* **13**, 55756-55765 (2021).

3. V. A. Cao, V. Q. Phan, N. K. Nguyen, M. Kim, P. C. Van, H. N. Minh, S. Y. Kim, J. Nah, Multifunctional tactile sensor with multimodal capabilities for pressure, temperature, and surface recognition. *Nano Energy* **136**, 110706 (2025).

4. Y. Liu, J. Wang, T. Liu, Z. Wei, B. Luo, M. Chi, S. Zhang, C. Cai, C. Gao, T. Zhao, S. Wang, S. Nie, Triboelectric tactile sensor for pressure and temperature sensing in high-temperature applications. *Nat Commun* **16** (2025).

5. J. Wade, T. Bhattacharjee, R. D. Williams, C. C. Kemp, A force and thermal sensing skin for robots in human environments. *Robotics and Autonomous Systems* **96**, 1-14 (2017).

6. G. Li, S. Liu, L. Wang, R. Zhu, Skin-inspired quadruple tactile sensors integrated on a robot hand enable object recognition. *Sci. Robot.* **5**, eabc8134 (2020).

7. Y. Liu, C. He, J. Fang, J. Zhou, K. Shang, X. Pu, L. Qian, T. Yang, A tri-modal tactile sensor based on porous ionic hydrogel for decoupled sensing of temperature and pressure. *Materials Today Physics* **41**, 101331 (2024).

8. J. Ren, X. Huang, R. Han, Y. Sun, G. Chen, Q. Li, Z. Zhou, Ultrahigh-Resolution Multimodal Tactile Sensors Enabled by Multi-Scale Conductive Network Construction and Band Engineering for Intelligent Perception. *Adv Funct Materials*, e12937 (2025).

9. W. Wu, H. Wang, X. Peng, F. Ni, L. Qiu, X. Wang, Pressure and temperature bimodal tactile sensor based on electrical double-layer effect of ionic liquids. *Sensors and Actuators A: Physical* **387**, 116377 (2025).

10. J. Pu, Y. Zhang, H. Ning, Y. Tian, C. Xiang, H. Zhao, Y. Liu, A. Lee, X. Gong, N. Hu, T. Zhang, S. Wang, Dual-Dielectric-Layer-Based Iontronic Pressure Sensor Coupling Ultrahigh Sensitivity and Wide-Range Detection for Temperature/Pressure Dual-Mode Sensing. *Advanced Materials*, e03926 (2025).

11. J. Ji, H. Luo, J. Su, S. Wang, X. Chen, J. Song, Multisensory electronic skin with decoupled pressure-temperature-sensing capabilities for similar object recognition. *Proc. Natl. Acad. Sci. U.S.A.* **122**, e2519693122 (2025).

12. X. Ma, C. Wang, R. Wei, J. He, J. Li, X. Liu, F. Huang, S. Ge, J. Tao, Z. Yuan, P. Chen, D. Peng, C. Pan, Bimodal Tactile Sensor without Signal Fusion for User-Interactive Applications. *ACS Nano* **16**, 2789-2797 (2022).

13. B. W. An, S. Heo, S. Ji, F. Bien, J.-U. Park, Transparent and flexible fingerprint sensor array with multiplexed detection of tactile pressure and skin temperature. *Nat Commun* **9**, 2458 (2018).

14. C. Han, Z. Cao, Z. An, Z. Zhang, Z. L. Wang, Z. Wu, Multimodal Finger-Shaped Tactile Sensor for Multi-Directional Force and Material Identification. *Advanced Materials* **37**, 2414096 (2025).

15. X. Yang, H. Ren, D. Guo, Z. Ling, T. Zhang, G. Li, Y. Tang, H. Zhao, J. Wang, H. Chang, G. T. Ki, J. Dong, W. Ningxin, Y. Shen, A Soft Tactile Unit with Three-Dimensional Force and Temperature Mathematical Decoupling Ability for Robots. *Engineering*, S2095809925001043 (2025).

16. J. Rao, Z. Chen, D. Zhao, R. Ma, W. Yi, C. Zhang, D. Liu, X. Chen, Y. Yang, X. Wang, J. Wang, Y. Yin, X. Wang, G. Yang, F. Yi, Tactile electronic skin to simultaneously detect and distinguish between temperature and pressure based on a triboelectric nanogenerator. *Nano Energy* **75**, 105073 (2020).

17. Y. Jo, Y. Lee, J. Kwon, S. Kim, G. Ryu, S. Yun, S. Baek, H. Ko, S. Jung, 3D active-matrix multimodal sensor arrays for independent detection of pressure and temperature. *Sci. Adv.* **11**, eads4516 (2025).

18. C. Ma, B. Zhu, Z. Qian, L. Ren, H. Yuan, Y. Meng, 3D-printing of conductive inks based flexible tactile sensor for monitoring of temperature, strain and pressure. *Journal of Manufacturing Processes* **87**, 1-10 (2023).

19. B. Liang, B. Huang, J. He, R. Yang, C. Zhao, B.-R. Yang, A. Cao, Z. Tang, X. Gui, Direct stamping multifunctional tactile sensor for pressure and temperature sensing. *Nano Res.* **15**, 3614-3620 (2022).

20. E.-I. Lee, H.-J. Ko, J. Kim, J.-W. Park, Self-powered single multifunctional tactile sensor for simultaneous detection of dynamic and static pressure and temperature inspired by skin sensory functions. *Nano Energy* **139**, 110984 (2025).

21. H. Zhu, H. Luo, M. Cai, J. Song, A Multifunctional Flexible Tactile Sensor Based on Resistive Effect for Simultaneous Sensing of Pressure and Temperature. *Advanced Science* **11**, 2307693 (2024).

22. C. Yu, L. Lindenroth, J. Hu, J. Back, G. Abrahams, H. Liu, A Vision-Based Soft Somatosensory System for Distributed Pressure and Temperature Sensing. *IEEE Robot. Autom. Lett.* **5**, 3323-3329 (2020).

23. Y. Wang, Y. Lu, D. Mei, L. Zhu, Liquid Metal-Based Wearable Tactile Sensor for Both Temperature and Contact Force Sensing. *IEEE Sensors J.* **21**, 1694-1703 (2021).

24. Y. Cao, Y. Peng, W. Ren, H. Wang, Y. Diao, Z. Liu, X. He, H. Luo, X. Wu, Intrinsically soft and fully recyclable robotic sensors with quadruple sensing functions for reliable human-robot interactions. *Science Bulletin*, S2095927325005249 (2025).

25. N. Yao, X. Wang, S. Ma, X. Song, S. Wang, Z. Shi, J. Pan, S. Wang, J. Xiao, H. Liu, L. Yu, Y. Tang, Z. Zhang, X. Li, W. Fang, L. Zhang, L. Tong, Single optical microfiber enabled tactile sensor for simultaneous temperature and pressure measurement. *Photon. Res.* **10**, 2040 (2022).

26. G. A. Gescheider, S. J. Bolanowski, K. L. Hall, K. E. Hoffman, R. T. Verrillo, The Effects of Aging on Information-Processing Channels in the Sense of Touch: I. Absolute Sensitivity. *Somatosensory & Motor Research* **11**, 345-357 (1994).

27. G. Westling, R. S. Johansson, Responses in glabrous skin mechanoreceptors during precision grip in humans. *Exp Brain Res* **66**, 128-140 (1987).

28. L. A. Jones, Perception of force and weight: Theory and research. *Psychological Bulletin* **100**, 29-42 (1986).

29. H.-N. Ho, Material recognition based on thermal cues: Mechanisms and applications. *Temperature* **5**, 36-55 (2018).

30. J. C. Stevens Kenneth K. Choo, Temperature sensitivity of the body surface over the life span. *Somatosensory & Motor Research* **15**, 13-28 (1998).

31. Y. Y. Shani, S. Lineykin, Thermal Cues Composed of Sequences of Pulses for Transferring Data via a Haptic Thermal Interface. *Bioengineering* **10**, 1156 (2023).

32. R. S. Johansson, A. B. Vallbo, Detection of tactile stimuli. Thresholds of afferent units related to psychophysical thresholds in the human hand. *The Journal of Physiology* **297**, 405-422 (1979).

33. S. A. Seah, M. J. Griffin, Thermotactile thresholds at the fingertip: Effect of contact area and contact location. *Somatosensory & Motor Research* **27**, 82-92 (2010).

34. D. Kumar, A. N. Silva, N. V. Thakor, "Neuromorphic Tactile Sensing and

Encoding" in *Handbook of Neuroengineering*, N. V. Thakor, Ed. (Springer Nature, Singapore, 2023; https://doi.org/10.1007/978-981-16-5540-1_117), pp. 1609-1634.

35. D. Deflorio, M. Di Luca, A. M. Wing, Skin and Mechanoreceptor Contribution to Tactile Input for Perception: A Review of Simulation Models. *Front. Hum. Neurosci.* **16**, 862344 (2022).

36. S. Gao, Y. Dai, A. Nathan, Tactile and Vision Perception for Intelligent Humanoids. *Advanced Intelligent Systems* **4**, 2100074 (2022).

37. E. B. Goldstein, G. W. Humphreys, M. Shiffrar, W. A. Yost, Eds., *Blackwell Handbook of Sensation and Perception* (Blackwell Pub, Oxford, UK ; Malden, MA, 2005)*Blackwell handbooks of experimental psychology*.

38. G. Masci, C. Cametti, Dielectric Properties of Thermo-Reversible Hydrogels: The Case of a Dextran Copolymer Grafted with Poly(*N*-isopropylacrylamide). *J. Phys. Chem. B* **113**, 11421-11428 (2009).

39. A. J. Bosman, E. E. Havinga, Temperature Dependence of Dielectric Constants of Cubic Ionic Compounds. *Phys. Rev.* **129**, 1593-1600 (1963).

40. T. Carvalho, V. Augusto, A. R. Brás, N. M. T. Lourenço, C. A. M. Afonso, S. Barreiros, N. T. Correia, P. Vidinha, E. J. Cabrita, C. J. Dias, M. Dionísio, B. Roling, Understanding the Ion Jelly Conductivity Mechanism. *J. Phys. Chem. B* **116**, 2664-2676 (2012).

41. S. Zhang, K. H. Lee, C. D. Frisbie, T. P. Lodge, Ionic Conductivity, Capacitance, and Viscoelastic Properties of Block Copolymer-Based Ion Gels. *Macromolecules* **44**, 940-949 (2011).

42. T. Carvalho, V. Augusto, Â. Rocha, N. M. T. Lourenço, N. T. Correia, S. Barreiros, P. Vidinha, E. J. Cabrita, M. Dionísio, Ion Jelly Conductive Properties Using Dicyanamide-Based Ionic Liquids. *J. Phys. Chem. B* **118**, 9445-9459 (2014).

43. N. Gao, J. Huang, Z. Chen, Y. Liang, L. Zhang, Z. Peng, C. Pan, Biomimetic Ion Channel Regulation for Temperature-Pressure Decoupled Tactile Perception. *Small* **20** (2024).

44. K. H. Lee, S. Zhang, T. P. Lodge, C. D. Frisbie, Electrical Impedance of Spin-Coatable Ion Gel Films. *J. Phys. Chem. B* **115**, 3315-3321 (2011).
